# Learning in higher dimensions: a strategy for alloy electrocatalyst discovery

**DOI:** 10.1039/d5ey00356c

**Published:** 2026-01-09

**Authors:** Vladislav A. Mints, Jack K. Pedersen, Gustav K. H. Wiberg, Jens Edelvang-Pejrup, Divyansh Gautam, Kirsten M. Ø. Jensen, Jan Rossmeisl, Matthias Arenz

**Affiliations:** a Department for Chemistry, Biochemistry and Pharmaceutical Sciences, University of Bern, Freiestrasse 3 3012 Bern Switzerland matthias.arenz@unibe.ch; b Department of Chemical Engineering, Imperial College London Imperial College Rd, South Kensington London SW7 2AZ UK; c Center for High Entropy Alloy Catalysis (CHEAC), Department of Chemistry, University of Copenhagen, Universitetsparken 5 2100 København Ø Denmark jan.rossmeisl@chem.ku.dk

## Abstract

In this work, we demonstrate the inversion of the classical bottom-up approach to drive the discovery of improved energy conversion electrocatalysts top-down. Starting with complex alloy catalysts of many constituents, we down-select to optimal materials by removing low-performing elements from the alloy. The efficiency of this data-driven approach arises from the fact that when studying many elements together in one material, information is also obtained on the less complex alloys that contain fewer constituents. Therefore, the number of experiments required to study the complex alloy is fewer than those needed for studying all constituent alloys individually. In addition, this top-down approach allows for a new way of comparing activity models constructed from experimental data with theoretical simulations. We introduce the approach by studying the Au–Ir–Os–Pd–Pt–Re–Rh–Ru high entropy alloy (HEA) composition space for the acidic oxygen reduction reaction (ORR). By studying 200 compositions, we created a machine-learned activity model and provide evidence that the model can predict the activity of underlying, less complex compositions that are contained in the Au–Ir–Os–Pd–Pt–Re–Rh–Ru HEA composition space.

## Abstract 2

In this work, we demonstrate the inversion of the classical bottom-up approach to drive the discovery of improved energy conversion electrocatalysts top-down.

## Graphical abstract

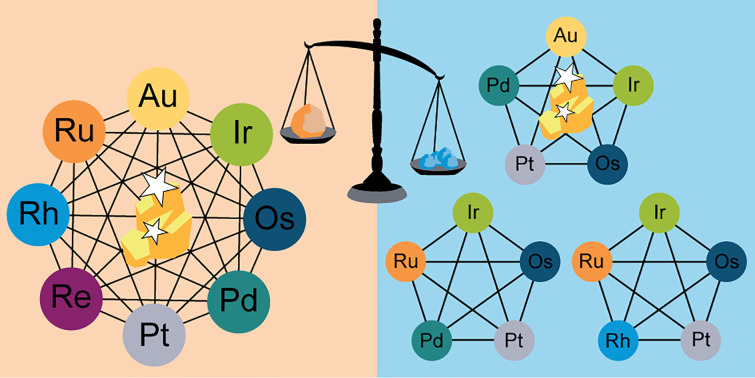

**Broader context**

Achieving carbon-neutrality is one of the most pressing challenges society is facing. A key step towards this goal is decarbonizing the chemical industry, which can be achieved through electrochemical processes leveraging efficient catalysts. High entropy alloys (HEAs), materials composed of at least five different elements, provide a vast and largely unexplored library of potential catalysts. However, this sheer number of possible HEA compositions makes it impossible to test them all, creating the need for smart exploration strategies. Here, we present a top-down approach to accelerate catalyst discovery. It starts with complex, multi-element alloy catalysts, which iteratively are simplified by removing the least active components until the most active composition is identified. The efficiency of this process arises from the fact that studying many elements together also yields information of all the simpler alloys they can form. We demonstrate this concept by studying Au–Ir–Os–Pd–Pt–Re–Rh–Ru alloys for the oxygen reduction reaction and identifying that the most active catalysts are composed of Au–Pd–Pt. This top-down approach offers a powerful new pathway for identifying optimal catalysts for the low-carbon chemical industry.

## Introduction

Design strategies for improved energy conversion electrocatalysts typically follow a bottom-up approach. Starting with simple, monometallic model systems of the most active element, one or several additional elements are added creating bi-metallic or multi-metallic surfaces. Thereby, the number of alloy constituents and the complexity of the catalyst are gradually increased, and theory is used as a means to rationalize the observed catalyst performance.^1^ For example, for the acidic ORR, one of the central electrocatalytic processes for energy conversion, Pt is the element with the highest catalytic performance, and the majority of research to identify improved ORR catalysts is rooted in experimental and theoretical studies of Pt single-crystal surfaces.^2–4^ Well-defined bimetallic Pt-single crystals, polycrystalline Pt-alloys, and Pt-alloy nanoparticles were studied in continuation of the early work demonstrating promising performance.^5–10^ Theoretical studies on these catalysts led to the descriptor approach and the discovery of the scaling relations which put a hard limit on the efficiency of ORR catalysts.^11,12^ The next level of complexity consists of ternary alloys, for which presently studies are gradually appearing.^13^ However, it has been pointed out by Cantor that in this approach to catalysis only the corners and edges of an, in principle, multidimensional composition space of catalytic materials are investigated.^14^ Consequently, in the last few years, the topic of HEA catalysts has gained significant attention.^15–19^ Comprising at least 5 elements, HEAs offer an expansive and largely unexplored array of extremely many compositions.^20^ In addition, based on pioneer work, it was proposed that HEA catalysts hold the key to overcome the limitations of state-of-the-art ORR electrocatalysts and, *e.g.*, break the scaling relations of the ORR.^21^ As such, there is a probability of finding novel ORR catalysts in the multidimensional HEA composition space. However, searching for the most active ORR catalyst in the form of nanoparticles using an experimental approach requires efficient approaches due to the vast number of possible compositions.

The “classical approach” for experimental studies, *i.e.*, starting with well-defined catalysts, and its combination with computational investigations originates from the times when data availability and computational power were severely limited. Hence, in electrocatalysis there was a preference to simplify models to one- or two-dimensional systems that can be visualized and fitted with simple linear models. Nowadays, large data volumes and computational power have become more accessible, which facilitates the use of machine learning tools and DFT-based computational screenings in electrocatalysis.^22–28^ Machine learning tools assist in the construction of multi-dimensional composition-activity models which allows for studying complex HEA composition spaces.^27–30^ Also, machine learning has been demonstrated to assist in the characterization of HEAs and aid the search for the most active catalyst composition.^31–34^ While these approaches are concerned with the fastest route to obtain an optimal catalyst composition through accelerated search, our approach focuses on extrapolating catalytic activities learned in the high dimension to sub-alloy composition spaces, enabling comprehensive exploration of the compositional landscape without investigating the sub-alloy spaces. Nevertheless, HEAs typically are studied first in their least complex shape. As such, much of the HEA research is limited to quinary alloys while only a fraction is exploring more complex HEAs.

**Fig. 1:**
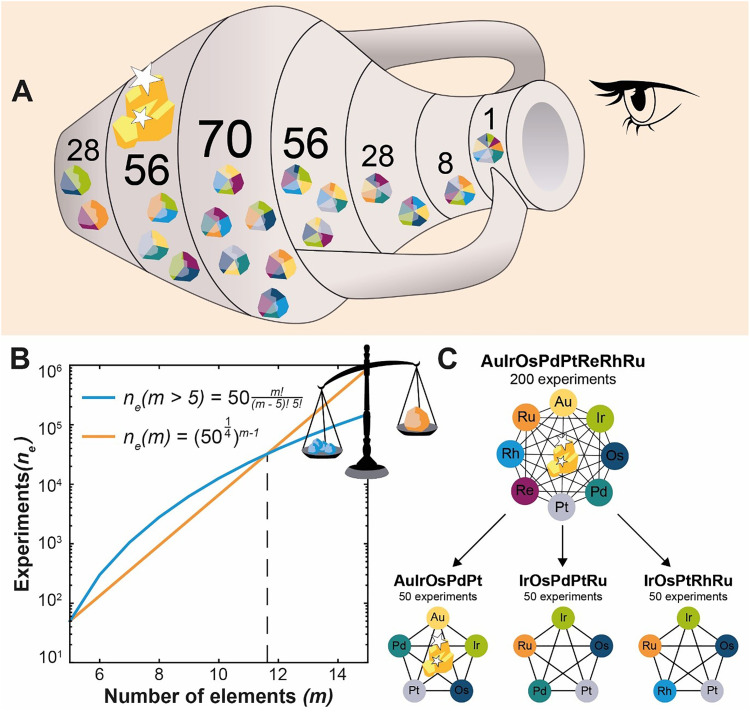
Depiction of the benefit of multi-dimensional modeling. (A) An artistic representation of the concept of multi-dimensional learning applied to this study. The layers in the amphora represent different multi-dimensional spaces that can be constructed from 8 elements, stacked based on their complexity. By looking through the top 8-element layer information on all underlying multi-dimensional spaces is obtained, which allows to directly identify the global most active three-element composition space. (B) Modeled information density in disordered alloys assuming 50 experiments in a 5-element space.^27^ (red) The number of experiments required to achieve the same sample density as with 50 experiments in the 5-element space (eqn (S1)–(S4)). (blue) The number of experiments required to study all possible 5-element combinations with 50 experiments that are part of a more complex alloy space (eqn (S5)). (C) Summary of the alloy compositions that were investigated.

Several studies have demonstrated that the most active HEA composition is not necessarily found at the near-equimolar composition.^35,36^ Therefore, in pursuit of the most active catalyst, it is crucial to study entire composition spaces. Based on our previous computational study, the number of experiments that are required to model a 5-element HEA composition space for the ORR is approximately 50.^27^ According to the “curse of dimensionality”, this number grows exponentially when more elements are included in the space (Fig. 1B, Supplementary Note I and Fig. S1, S2). However, we argue that by starting with a higher dimensional HEA composition space, all underlying 5-element HEA composition spaces are automatically included in the study. Up to 11 elements, the number of experiments required to study the combined high-dimensional HEA composition space is less than when studying all 5-element HEA composition spaces separately. In addition, the latter do not contain any information about HEAs with more than 5 components. This makes it more favorable to study more elements together than limiting studies to fewer components in the HEA.

## Results and discussion

### Predicting 5-element compositions with 8-element experiments

**Fig. 2:**
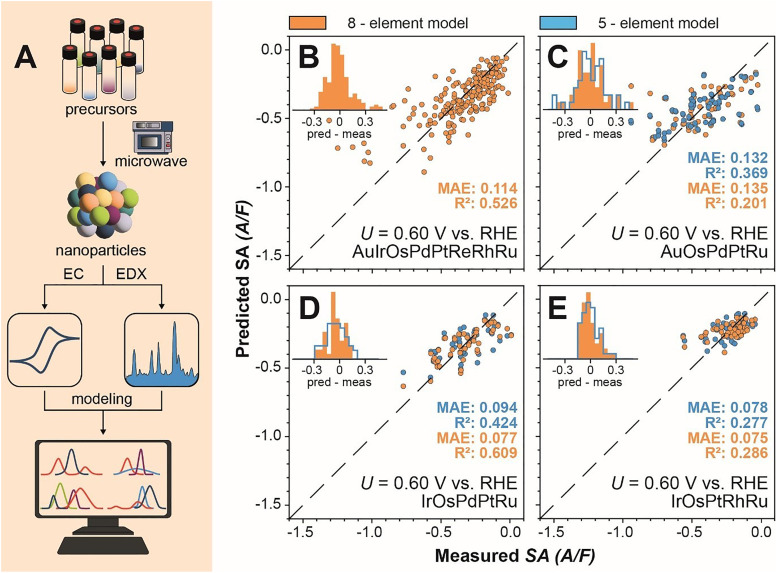
Workflow and predictive performance of models. (A) The workflow for data collection. Starting with the microwave synthesis the obtained nanoparticles were characterized with EDX and evaluated for the electrocatalytic ORR performance. The acquired data was used to train Gaussian process models. (B)–(E) Leave-one-out parity plots showing the measured ORR current density plotted against the prediction of a Gaussian process regressor trained on all samples except the sample being predicted for the Au–Ir–Os–Pd–Pt–Re–Rh–Ru 8-element model (B), as well as the Au–Os–Pd–Pt–Ru (C), Ir–Os–Pd–Pt–Ru (D), and Ir–Os–Pt–Rh–Ru (E) 5-element models. For the 5-element alloys a constant scaling factor was multiplied on the predicted values (Fig. S13–S17). The measured current density is extracted from the polarization curves at a potential of 0.60 V *vs.* RHE and divided by the mean capacitance in the potential region from 0.30 to 0.50 V *vs.* RHE. A/F: amperes per farad. MAE: mean absolute error in A/F.

We tested this hypothesis by constructing an experimental data-driven model of the 8-element Au–Ir–Os–Pd–Pt–Re–Rh–Ru HEA composition space for the acidic ORR. The aim was to demonstrate that this model not only contains the role of each of the eight elements for the catalytic reaction but that it is also possible to approximate the optima of the underlying 56 5-element HEA composition spaces and estimate their activity. An artistic representation of this new concept of “multi-dimensional learning” applied to this study is depicted in Fig. 1A, while the workflow we employed to study the different HEA compositions, is outlined in Fig. 2A.

The ORR electrocatalysts were prepared by adopting a solvothermal synthesis route that previously was shown to produce HEA nanocatalysts.^37^ In this synthesis, chloride-based metal precursor salts were dissolved in ethanol and heated at a pressure of 20 bar (setpoint) for 30 minutes in a microwave reactor. This produced agglomerated nanoparticles with crystallite sizes larger than 5 nm. The precursor mixtures were selected using a grid that could be dynamically extended.^38^ In the end, 200 different nanoparticle compositions in the Au–Ir–Os–Pd–Pt–Re–Rh–Ru space were synthesized, and 50 different compositions in the Au–Os–Pd–Pt–Ru, Ir–Os–Pd–Pt–Ru, Ir–Os–Pt–Rh–Ru spaces each (Fig. 1C).

The compositions of the as-synthesized nanoparticles were evaluated with energy dispersive X-ray spectroscopy (EDX). For selected samples their structure was analyzed with transmission electron microscopy (TEM) and X-ray diffraction (XRD), the latter showing mixed face-centered cubic (fcc) and hexagonal closed packed (hcp) phases in most of the samples (Fig. S3, S4 and Tables S1–S5). The EDX compositions formed the input for the machine-learned Gaussian processes (GP) (Fig. S5 and eqn (S6), (S7)).

Using the Pearson correlation coefficients (Fig. S6) it is demonstrated that the elements do not have any strong correlation with each other. Thus, all composition spaces are sampled randomly. Upon inspecting the compositions, we observed that Re had an average concentration of less than 3 atomic percent (at%) in contrast to the expected 12 at% (Fig. S7). Therefore, Re is mostly absent in the HEA particles and no conclusions on its role could be made.

We evaluated the ORR activity of the nanoparticles using a multi-electrode setup (Fig. S8) in a “three-electrode configuration”. The multi-working electrode allowed the simultaneous study of six catalytic films of the same catalyst. The measurements started with a cyclic voltammogram (CV) between 0 to 0.6 V *vs.* reversible hydrogen electrode (RHE). From these measurements, the capacitance was extracted in the potential window of 0.3 to 0.5 V *vs.* RHE (Fig. S9–S12). This capacitance was used to normalize the measured currents to correct for possible loading and surface area differences. After the CV, the particles were oxidized at 1 V *vs.* RHE for 20 minutes, while the electrolyte was being saturated with O_2_. Following, the potential was stepped down to 0.6 V *vs.* RHE with steps of 10 mV each lasting for 20 seconds.

With the obtained experimental data, we constructed GP models for the ORR activity-composition relationship in each of the four composition spaces. The GP models correlated the EDX compositions of the particles with the specific activity at 0.6 V *vs.* RHE measured at quasi-steady-state conditions. The GPs were fitted with a radial basis function kernel with optimized length scales of 0.22, 0.25, 0.28, and 0.13 as well as a white noise kernel with noise values of 0.41, 0.47, 0.45, and 0.40 for Au–Ir–Os–Pd–Pt–Re–Rh–Ru, Au–Os–Pd–Pt–Ru, Ir–Os–Pd–Pt–Ru, and Ir–Os–Pt–Rh–Ru respectively. These fitted length scales and noise levels indicate that the composition-activity relationships in this work are explained by smooth mathematical functions with relatively strong correlations between the activity of compositions with large Euclidean distances between them. These length scales are comparable to the one observed in our previous study^27^ justifying the assumption that 50 experiments are required to study a single 5-element space. Combined, these observations indicate that our studies of HEA electrocatalysts require only a limited number of experiments to model the activity-composition relationship.

The performance of the GP models was tested with the leave-one-out cross-validation (LOOCV) (Fig. 2B–E). The 8-element model predicts the activities with a mean absolute error (MAE) of 0.11 A F^−1^ and an *R*^2^ of 0.53. Similarly, the mean absolute error can be used to evaluate the prediction of 5-element spaces by the 8-element model. The 8-element model predicts the Au–Os–Pd–Pt–Ru space with a MAE of 0.14 A F^−1^ and an *R*^2^ of 0.20, whereas the LOOCV score of the model itself is 0.13 A F^−1^ with an *R*^2^ of 0.37. In this composition space, as indicated by the histogram, 6 samples have an error larger than l0.3l A F^−1^. These few samples exhibit high leverage on the calculation of the MAE and *R*^2^. Removing these points from the calculation, but not from the fitting, leads to an MAE of 0.1 A F^−1^ and an *R*^2^ of 0.6 for both models (Fig. S13). The Ir–Os–Pd–Pt–Ru space was predicted with a MAE of 0.08 A F^−1^ and an *R*^2^ of 0.61, which is an improvement over the LOOCV score of 0.09 A F^−1^ and an *R*^2^ of 0.42. Likewise, the Ir–Os–Pt–Rh–Ru space is predicted by the 8-element model with a MAE of 0.075 A F^−1^ and *R*^2^ of 0.28 which is similar to the LOOCV score of 0.078 A F^−1^ and *R*^2^ of 0.28. This composition space is mostly inactive leading to a relatively flat activity landscape. As a result, the GPR is struggling to find meaningful correlations. However, since this space is mostly inactive, it is also of lesser interest for future works.

For all the 8-element model predictions a multiplicative bias correction was applied to account for systematic differences with the 5-element models (Fig. S14–S17). As the bias correction is multiplicative, the projections of the 5-element spaces maintain their landscapes. In other words, the absolute activity values change but the correlations that influence the predictions as well as the positions of the optima are not altered. We therefore conclude that the 8-element model indeed learned a very similar landscape for the 5-element spaces as their corresponding 5-element models themselves in agreement to our hypothesis.

### Extrapolating predictions to all 5-element subspaces

**Fig. 3:**
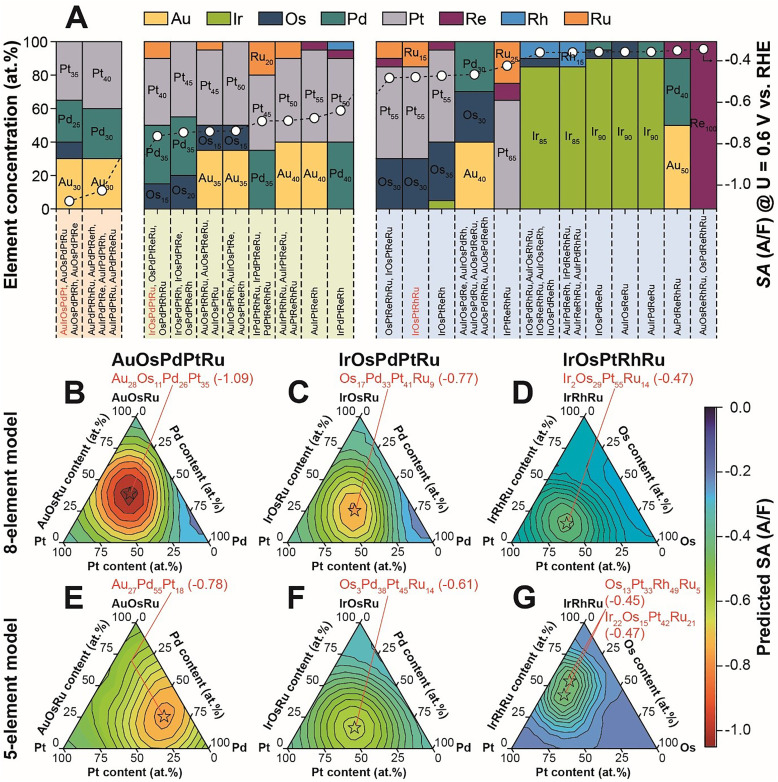
Optima in composition spaces. (A) Optimal compositions for ORR in all the 56 five-element composition spaces contained in the 8-element composition space as predicted by the 8-element model in a 5 at% grid scan of the composition space. The three investigated 5-element HEAs are highlighted in red. (B)–(G) Most active compositions of 5-element HEAs shown as pseudo-ternary plots with the molar concentrations summed for three of the elements. Predictions by the 8-element model (B)–(D), and the individual 5-element models (E)–(G) for Au–Os–Pd–Pt–Ru (B) and (E), Ir–Os–Pd–Pt–Ru (C) and (F), and Ir–Os–Pt–Rh–Ru (D) and (G). The optimal predicted compositions in each composition are annotated in red. Confidence intervals for B–G are shown in Fig. S18.

Using the 8-element model, we mapped out all possible optima of the 56 5-element HEA composition spaces (Fig. 3A). These can be classified based on their maximum activity into three classes. The first class has optima with an ORR activity below 0.6 A F^−1^, making them ill-suitable for catalytic applications. The second class has optima with activities between 0.6 and 0.8 A F^−1^. Distinctively, these optima contain a combination of Pt with Pd or Au. The third class, composed of optima with the highest ORR activity, is most interesting for catalytic application. Their optima are composed of a combination of Pt, Pd, and Au.

Analyzing the results in more detail, we investigated three 5-element models each representing one of the three separate activity classes (Fig. 3B–G and Fig. S18). Starting with the low activity class, the Ir–Os–Pt–Rh–Ru model shows the existence of two distinct activity optima at Os_13_Pt_33_Rh_49_Ru_5_ and Ir_22_Os_15_Pt_42_Ru_21_ (Fig. 3G). On the other hand, the 8-element model predicts only a single optimum (Fig. 3D). As the ORR activity of the compositions in this space is low, the GPR struggles to identify the major correlations in the landscape. As a result, the optima predicted by both the 5-element and 8-element models might be artificial. The Ir–Os–Pd–Pt–Ru model, which represents the middle activity class, contains an optimum at Os_3_Pd_38_Pt_45_Ru_14_ (Fig. 3F). The 8-element model predicts a similar optimum at Os_17_Pd_33_Pt_41_Ru_9_ (Fig. 3C), which has an Euclidian distance of 11 at% in Cartesian coordinates. Finally, the Au–Os–Pd–Pt–Ru model representing the high active class predicts the optimum at Au_27_Pd_55_Pt_18_ (Fig. 3E), whereas the global optimum of the 8-element model is Au_28_Os_11_Pd_26_Pt_35_ (Fig. 3B). Further analysis, which is discussed below, shows that the Os content has no distinct correlation to the ORR activity in these HEA catalysts. This suggests that the Os in the global optimum of the 8-element model is an artifact.

### Navigating a smooth composition-activity landscape

**Fig. 4:**
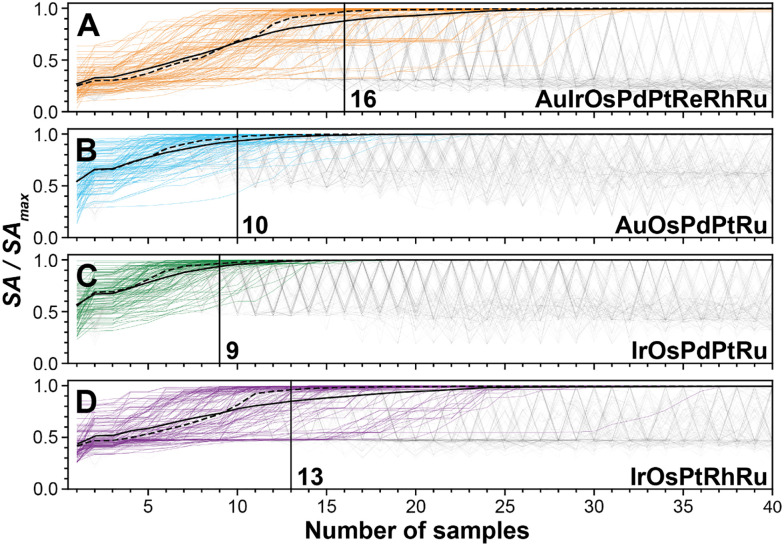
Number of experiments needed to obtain the optimal composition. (A)–(D) Bayesian optimizations of the number of compositions needed to obtain the optimal composition in the 8-element Au–Ir–Os–Pd–Pt–Re–Rh–Ru composition space (A), as well as the 5-element Au–Os–Pd–Pt–Ru (B), Ir–Os–Pd–Pt–Ru (C), and Ir–Os–Pt–Rh–Ru (D) composition spaces. The optimization is performed on the GPR models of each composition space. Each of the 100 faint, grey lines represents an alloy activities identified by an individual Bayesian optimization initialized with two randomly chosen compositions. The solid (dashed), black lines show the expectation (median) value of the highest absolute current density found after a given number of samples. The number of samples needed for the median to reach 95% of the value of the current density of the global optimum (*i.e.*, the number of samples where 50% of the optimizations are sufficiently close to the global optimum) are annotated to estimate the number of experiments needed to find the global optimum.

Using these GPR models, we can evaluate the approximate number of experiment required for Bayesian optimization to optimize these composition spaces, see Fig. 4. On average, 16 experiments are required to find the global optimum of the 8-element model. For the 5-element models, it took 8 experiments for the Au–Os–Pd–Pt–Ru, 9 experiments for the Ir–Os–Pd–Pt–Ru, and 13 experiments for the Ir–Os–Pt–Rh–Ru model. Thus, our optimization experiments indicate that the number of experiments required to optimize a model does not scale exponentially with increasing the complexity of the HEA composition space. The latter would be the case for grid search studies according to the “curse of dimensionality”.

The reason for this “milder” scaling is that the optimization algorithms depend mostly on the complexity of the mathematical landscape. Therefore, we propose that if elements have a minor contribution on the reaction, adding them to an optimization study will marginally increase the complexity of the mathematical landscape and correspondingly marginally affect the experimental demand. Consequently, optimizing HEA composition spaces with as many elements as possible becomes even more beneficial, when in search of the most active catalyst.

### Assessing the activity in ternary Au–Pd–Pt composition space

**Fig. 5:**
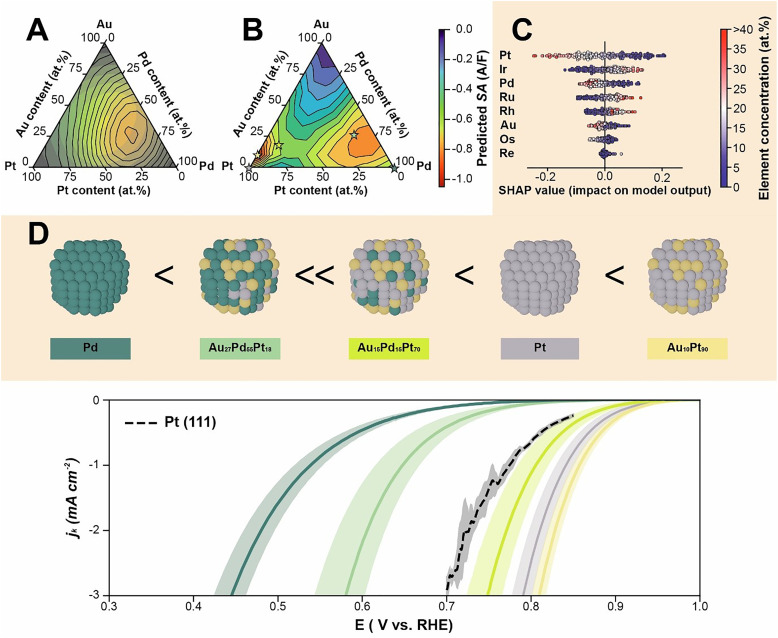
Highest activity found in the Au–Pd–Pt ternary subspace. (A) and (B) ORR catalytic activities in the Au–Pd–Pt composition space as predicted by the 5-element Au–Os–Pd–Pt–Ru model (A), and with DFT simulations (B). The circular overlay in (A) corresponds to the GPR prediction certainty. The coloring of the DFT prediction has been normalized such that the optimum at Au_27_Pd_55_Pt_18_ in (A) is given the same color in (B). The stars depict nanoparticle compositions that have been additionally investigated using the rotating disk electrode (RDE). (C) The influence of the individual element concentrations on the catalytic activity of each experiment in the 8-element model obtained with a SHAP analysis. (D) Supporting experiments investigating promising nanoparticle compositions using RDE experiments (Fig. S19–S33). The measurements were carried out in 0.1 M H_2_SO_4_, at 1600 RPM with a scan rate of 10 mV s^−1^. The kinetic current has been extracted using the Koutecky–Levich equation (eqn (S8)).

In the following, we demonstrate how these highly dimensional, experimental data-driven models can be used to analyze the contributions of the individual elements to the catalytic activity. Using the 8-element model, we evaluated the correlations of the elements to the ORR activity using SHapely Additive explanations (SHAP) (Fig. 5C).^39^ The SHAP values show that the element that has the largest positive influence on the ORR is platinum. This result agrees with the established conclusion that Pt is the most active element for the ORR.^40,41^ The next element that according to the SHAP analysis has a strong positive impact on the ORR is Pd. Pd, similar to Pt, lies close to optimum in established ORR volcano plots and is being investigated as a substitute for Pt.^4,42–44^ The final element that improves the ORR reaction according to our analysis is Au. While Au itself is not very active for the acidic ORR,^45,46^ its alloys with Pt^47,48^ and Pd^49,50^ have been reported previously to improve the ORR activity. Together, these three elements are responsible for the optimum of this 8-element HEA composition space. On the other hand, the SHAP analysis suggests that under the chosen experimental conditions Ir, Ru and Rh are the “least important” elements to promote the ORR. However, this does not concern possible stabilizing effects which were not evaluated here. The analysis of Os shows only a weak correlation to the ORR. Therefore, its contributions to the ORR activity have a large error margin, which explains its artificial presence in the 8-element model optimum, see above. Lastly, Re, which was not fully incorporated in the alloy, did not show a correlation with the models prediction, indicating that it is mathematically a harmless spectator.

The experimental data-driven models derived from HEA studies allow a unique comparison to computational studies. Typically, a close resemblance between the surfaces studied in computational simulations and experimental studies is only achieved with well-defined single-crystal surfaces and individual activities. By contrast, comparisons between data-driven models and computational models reveal activity trends and bring a new quality to testing the predictive power of computational simulations. In our specific case, the 8-element model and the Au–Os–Pd–Pt–Ru model both agree that the most active subspace is Au–Pd–Pt. We visualized the activity of the Au–Pd–Pt space in a ternary plot (Fig. 5A). We then performed DFT calculations of this composition space and constructed an equivalent “theory-based” activity model for the ternary Pt–Pd–Au composition space (Fig. 5B). This provides a visual comparison between “theory-based” and experimental data-driven activity modeling. It is seen that the experimental data-driven model exhibits an optimum at Au_27_Pd_55_Pt_18_, with a soft gradient towards the edges. The DFT model, on the other hand, shows a local optimum at Au_21_Pd_72_Pt_7_, which is remarkably close to the experimentally predicted Au_27_Pd_55_Pt_18_ optimum. However, the DFT model also calculates a global optimum at Au_7_Pt_93_ with even higher ORR activity and a strong minimum at pure Au. Both this optimum and minimum are located near the mono-metallic corners of the composition space. It is therefore important to point out that the experimental data-driven models that are built to span a HEA composition space lack data in the (typically known) corner and edge regions of the composition space. As a result, data-driven models are unable to extrapolate to the vertices and binary regions, which in statistical models is reflected in an increased prediction uncertainty. In the present case, this leads to the observed discrepancy and highlights the power of combining computational and experimental studies.

Based on the DFT calculations and the machine-learned model, we investigated the promising 3-element compositions, Au_10_Pt_90_, Au_15_Pd_15_Pt_70_ and Au_27_Pd_55_Pt_18_ using the rotating disk electrode (RDE) (Fig. 5D and Fig. S19–S33). As a reference, Pt and Pd nanoparticles and Pt(111) were used. In line with the work of Lankiang and co-workers^47^ and the DFT calculations Au_10_Pt_90_ is more active than pure Pt. However, the previously reported global optimum of Au_15_Pd_15_Pt_70_^47^ could not be confirmed, which may be attributed to the use of H_2_SO_4_ instead of HClO_4_ as electrolyte.^3^ The predicted local optimum of Au_27_Pd_55_Pt_18_ did show an activity higher than Pd but not as high as Pt. We attribute this to the difference in surface composition. In the dataset that the machine learning algorithm used, the closest datapoint to the local optimum is Au_13_Os_6_Pd_53_Pt_14_Ru_13_ (Fig. S34). Au_13_Os_6_Pd_53_Pt_14_Ru_13_ has a high ratio of charge transferred to the hydrogen under potential deposition region to the double region compared to the case of Au_27_Pd_55_Pt_18_ nanoparticles (Fig. S35). This indicates that in Au_27_Pd_55_Pt_18_ nanoparticles Au may have segregated to the surface, which is catalytically less active for the ORR than Pt or Pd. Therefore, while the machine-learned models predict that the Au_27_Pd_55_Pt_18_ composition has a high probability of yielding an alternative catalyst, precise morphology engineering is required to produce it. Last but not least, the influence of the capacity normalization was investigated confirming Pt-Pd–Au as the most active combination (Fig. S36 and S37).

## Conclusions and outlook

We propose an inversion of the classical bottom-up approach to studying electrocatalysts. Instead of gradually increasing the complexity of an electrocatalyst, we argue that catalytic information is obtained more efficiently when starting from complex HEA composition spaces. As a complex composition space contains information on all constituent catalysts with fewer components, its optimum will correspond to the global optimum across all its sub-spaces. In addition, the data can be used to produce a map of the optima of its different subspaces and provides argumentation on which element combinations can be ignored in later studies and which are worth investigating further. Furthermore, the experimental data-driven activity models can be compared to “theory-based” activity models both testing the predictability of computational simulations with a new quality as well as offering to accelerate catalyst discovery significantly.

We demonstrate this approach of a HEA discovery platform by studying the 8-elemental Au–Ir–Os–Pd–Pt–Re–Rh–Ru composition space for the ORR using microwave-based nanoparticle synthesis and multi-electrode electrochemical activity experiments. The Au–Ir–Os–Pd–Pt–Re–Rh–Ru model mapped out effectively the optima of HEA spaces with fewer elements and provided an analysis of the contributions of the individual elements to the catalytic activity. As most contributing elements Pt, Pd and Au are identified. The highest activity is obtained for a combination of all three elements and the comparison of the data-driven model and the DFT model points towards highly active ternary Au–Pd–Pt compositions. However, the limitations of the regression models in constructing activity maps are also highlighted. Extensive extrapolations in data-driven models beyond experimentally sampled compositions can lead to large uncertainties. Computational simulations can therefore accelerate the catalyst discovery substantially, but also automated synthesis robots coupled to the demonstrated accelerated electrocatalytic testing will allow experimental sampling of larger areas of interest.

## Conflicts of interest

There are no conflicts of interest to declare.

## Supplementary Material

EY-004-D5EY00356C-s001

## Data Availability

All data and scripts that support the findings of this study have been made freely available at https://www.erda.dk/archives/2e841664fe6566c42ffb258419dcd21a/published-archive.html. Supplementary information contains experimental and computational details, XRD, EDX, TEM, SEM and electrochemical characterizations, as well as supporting figures and discussion. See DOI: https://doi.org/10.1039/d5ey00356c.
