# Supplementary material for: Learning in higher dimensions: a strategy for alloy electrocatalyst discovery
Source: EES Catal. 2026 Jan 9;4(2):397–406. doi: 10.1039/d5ey00356c (PMC12805875; doi:10.1039/d5ey00356c)
Supplement: EY-004-D5EY00356C-s001 [file EY-004-D5EY00356C-s001.pdf]

Supplementary Information (SI) for EES Catalysis.
This journal is © The Royal Society of Chemistry 2026

Supplementary Materials for

Learning in Higher Dimensions: A Strategy for Alloy Electrocatalyst Discovery

Vladislav A. Mints[1,2 #], Jack K. Pedersen[3, #], Gustav K. H. Wiberg[1], Jens Edelvang-Pejrup[3], Divyansh Gautam[1], Kirsten M. Ø. Jensen[3], Jan Rossmeisl[3*], Matthias Arenz[1,3*]

[1] Department for Chemistry, Biochemistry and Pharmaceutical Sciences, University of Bern, Freiestrasse 3, 3012, Bern, Switzerland.

[2] Department of Chemical Engineering, Imperial College London, Imperial College Rd, South Kensington, London SW7 2AZ, United Kingdom

[3] Center for High Entropy Alloy Catalysis (CHEAC), Department of Chemistry, University of Copenhagen, Universitetsparken 5, 2100 København Ø, Denmark.

[#] equally contributing first authors

* Corresponding authors: jan.rossmeisl@chem.ku.dk; matthias.arenz@unibe.ch;

I. Equation for the information density of a composition

The volume of a simplex space with ridge length *a* and *m* different metals is given by Eq. S1. A high-entropy alloy composition space is described by a simplex with vertices at (1,0,0…) (0,1,0,…) etc. and a ridge length of $a = \sqrt{2}$. When this space is uniformly studied, each experimental sample occupies a fraction of the volume equal to $V(m, \sqrt{2}) / n_e$. This fraction of the volume we describe as a simplex with length *a* (Eq. S2). The sample density is inversely proportional to the size of the simplex volume. Therefore, the sample density can be estimated using Eq. S3. This equation is in line with machine-learning literature[1] that states that the sampling density is proportional to $n^{p^{-1}}$ where p is the dimensionality.

$$V(m,a) = \frac{\sqrt{m-1+1}a^{m-1}}{(m-1)!\sqrt{2^{m-1}}} = V(m,1)a^{m-1} \quad \text{(Eq. S1)}$$

$$a(m,n_e) = \left(\frac{V(m,\sqrt{2})}{V(m,1)n_e}\right)^{\frac{1}{m-1}} = \left(\frac{V(m,1)\sqrt{2^{m-1}}}{V(m,1)n_e}\right)^{\frac{1}{m-1}} = \sqrt{2}\left(\frac{1}{n_e}\right)^{\frac{1}{m-1}} \quad \text{(Eq. S2)}$$

$$\rho(m,n_e) \propto \frac{1}{a(m,n_e)} = \frac{1}{\sqrt{2}} n_e^{\frac{1}{m-1}} \quad \text{(Eq. S3)}$$

Not all simplex geometries can be uniformly filled with the same geometry of smaller simplexes. Therefore, an alternative equation to Eq. S3 can be used which describes the median between the origin and the closest data point in a unit cube (Eq. S4), as described by Hastie et al.[1] Since a simplex geometry is a slice of a unit cube, we assume that the relations in this equation also hold true for a simplex space. Independent of whether Eq. S3 or Eq. S4 are used to determine the number of experiments in higher dimensions, both lead to the identical conclusion that a region exists where studying the combined higher-dimensional system is more efficient (Fig. S2).

$$d(m,n_e) = \left(1 - \left(\frac{1}{2}\right)^{\frac{1}{n_e}}\right)^{\frac{1}{m-1}} \quad \text{(Eq. S4)}$$

The number of experiments that are required to study all possible compositions containing m_2 metals from a pool of m_1 metals can be calculated using Eq. S5. In this equation m_1 is the number of metals in the overarching composition space, m_2 the number of elements of a given subspace, and n_{e2} the number of experiments that are necessary to study that given subspace.

$$n_e(m_1,m_2,n_{e2}) = \frac{m_1!}{(m_2-m_1)!m_1} n_{e2} \quad \text{(Eq. S5)}$$

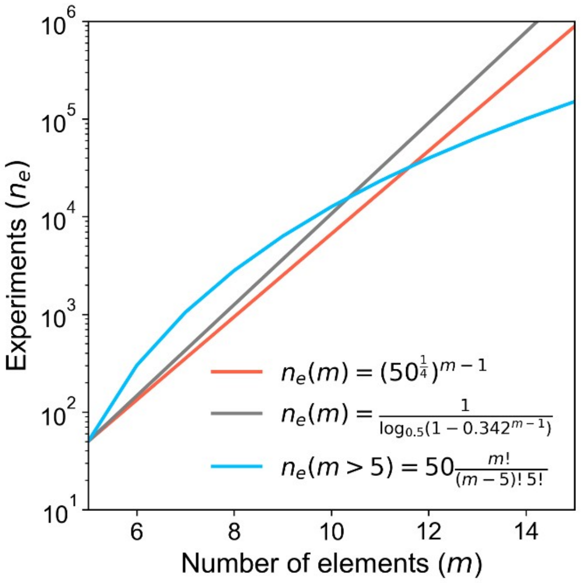

Fig. S1. Modelled information density in disordered alloys. (red, grey) The number of experiments required to achieve the same sample density as with 50 experiments in the 5-element space. (blue) The number of experiments required to study all possible 5-element combinations that are part of a more complex alloy space.

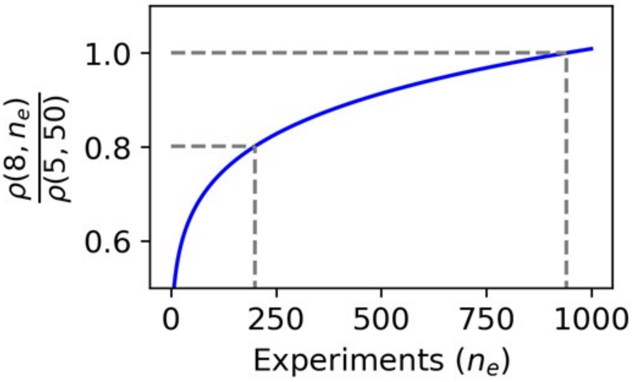

Fig. S2. Sample density for an 8-element composition space with n_e experiments. The dashed lines show the normalized sample density of 0.8 at 200 experiments and 1 at 940 experiments.

According to Eq. S3, 940 experiments in the 8-element space are required to get the same sample density as with 50 experiments in the 5-element space.[2] However, we made the trade-off to do 200 experiments based on Fig. S1. With 200 experiments a normalized sample density of 0.8 is achieved, while a sample density of 1 is desired. To achieve this sample density increase of around 25%, around four times more experiments would be required.

Note, that if the composition space is more complex and more experiments are required to study a 5-element space, e.g., studying a different reaction, Fig. S1 can be recalculated using an updated assumption for the number of experiments required to study the 5-element space. Assuming Eq. S3, only when 1296 or more experiments are required to study the 5-element space, it is no longer beneficial to increase the number of elements. Assuming Eq. S4, this limit becomes 896 experiments in the 5-element space.

II. Complementary XRD measurements

Complementary X-ray diffraction (XRD) experiments on randomly selected samples were measured using a STOE StadiP with a Cu Kα X-ray source in transmission geometry. Transmission electron microscopy (TEM) images were recorded at the Microscopy Imaging Center of the University of Bern by Dr. Jia Du with a FEI Tecnai Spirit transmission electron microscope at 80 kV with an Olympus-SIS Veleta CCD Camera.
The powder X-ray diffraction (PXRD) data was fitted using the Topas Academic 7 software[3]. The instrumental peak shape was obtained by fitting a standard (Si or Er_2O_3) and locking the resulting parameters during refinements. The free parameters for each phase were lattice parameters, crystallite size (with a Gaussian and a Lorentzian contribution) and microstrain (with a Lorentzian contribution). Both fcc and hcp are modelled as single-element Pd, since its electron density falls somewhere in between all the present metals. In all refinements, the B_{iso} values were fixed to 0.24 Å^2.
Each sample is described by 1-4 different phases, of which 1-2 are face-centered cubic (fcc) and 0-2 are hexagonal close-packed (hcp). These are the two crystal structures the component metals are expected to exhibit. Table S1 shows which phases are present in the different samples, and how they contribute to the description of the data. The fitting was carried out by initially fitting one fcc phase to each sample. Depending on the difference curve between calculated and observed data, an additional fcc or hcp phase was added to the fit. A third phase, either fcc or hcp, was sometimes added in the same way based on what best described the difference curve of this fit. In a few cases a fourth phase was added to the fit in the same way. Most samples are best described by fitting multiple phases, yet as shown in the work of Yeh this does not exclude the presence of high entropy alloys[4].
A phase was generally only added when its associated peaks clearly corresponded to peaks in the difference curve, since blindly adding phases would improve the fit even if the phases were not justifiably present. However, this distinction is harder to make as the particles grow smaller and the data quality is limited. We often observed a discrepancy between the model and observed data around 40-45° (between the 111 and 200 reflections), which has also previously been observed for HEA nanoparticles described by simple fcc and hcp phases[5,6]. In the nicer PXRD datasets, an hcp phase gave a clear, meaningful contribution to the fit in this region. However, in datasets with broader, less distinguishable peaks, it was less clear whether an hcp phase was present, since the lattice parameters and size of the hcp were difficult to control during fitting. This is why the presence of an hcp phase is inconclusive in some of the entries of Table S1. This also means that the lattice parameters, size, strain and relative phase contributions of the hcp cannot always be trusted in these datasets.

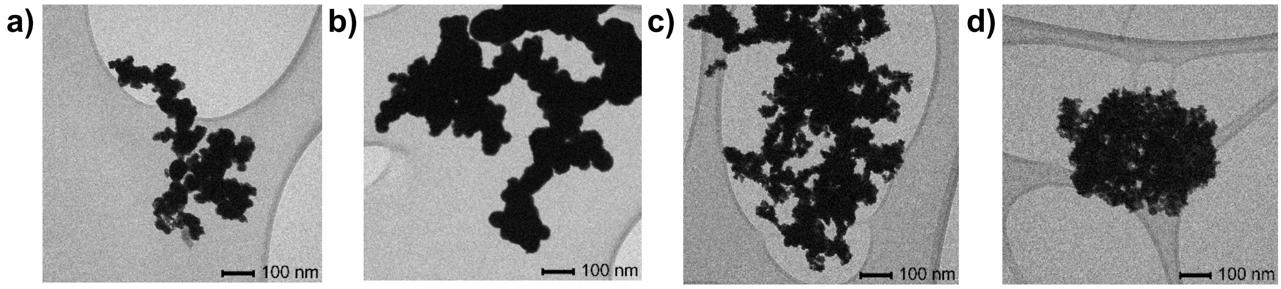

Fig S3. TEM characterization of selected nanoparticles (a) Sample: $Au_{16}Ir_{15}Os_{16}Pd_{16}Pt_{11}Re_1Rh_{11}Ru_{14}$, (b) Sample: $Au_{25}Os_{14}Pd_{21}Pt_{19}Ru_{21}$, (c) Sample: $Ir_{23}Os_{10}Pd_{24}Pt_{25}Ru_{18}$, (d) Sample: $Ir_{13}Os_{34}Pt_{13}Rh_{21}Ru_{18}$.

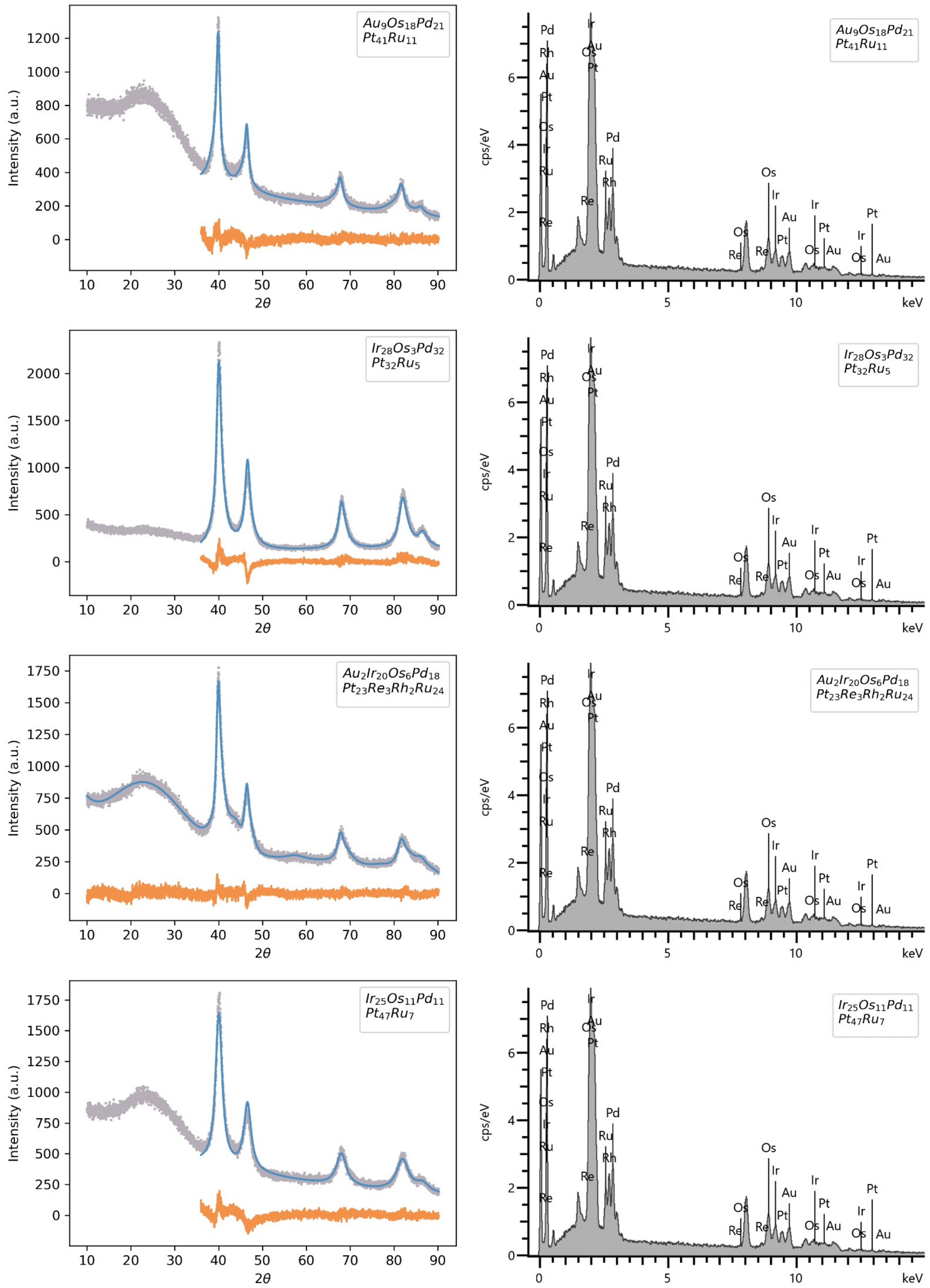

$Au_9Os_{18}Pd_{21}$ $Pt_{41}Ru_{11}$
$Ir_{28}Os_3Pd_{32}$ $Pt_{32}Ru_5$
$Au_2Ir_{20}Os_6Pd_{18}$ $Pt_{23}Re_3Rh_2Ru_{24}$
$Ir_{25}Os_{11}Pd_{11}$ $Pt_{47}Ru_7$
Intensity (a.u.)
2θ
cps/eV
keV
Pd
Rh
Au
Pt
Os
Ir
Ru
Re

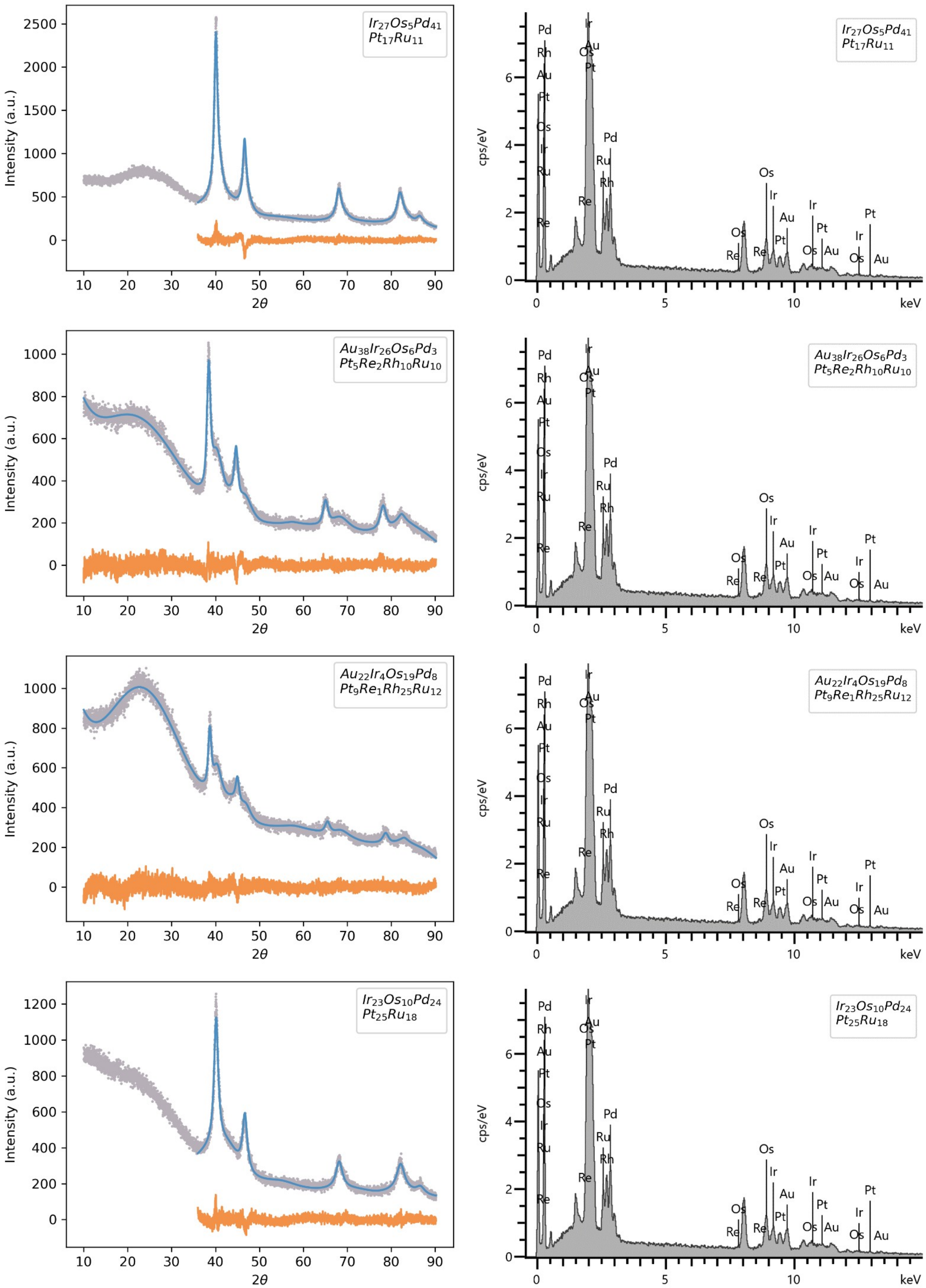

$Ir_{27}Os_5Pd_{41}Pt_{17}Ru_{11}$
$Au_{38}Ir_{26}Os_6Pd_3Pt_5Re_2Rh_{10}Ru_{10}$
$Au_{22}Ir_4Os_{19}Pd_8Pt_9Re_1Rh_{25}Ru_{12}$
$Ir_{23}Os_{10}Pd_{24}Pt_{25}Ru_{18}$
Intensity (a.u.)
2θ
cps/eV
keV
Pd
Rh
Au
Pt
Os
Ir
Ru
Re

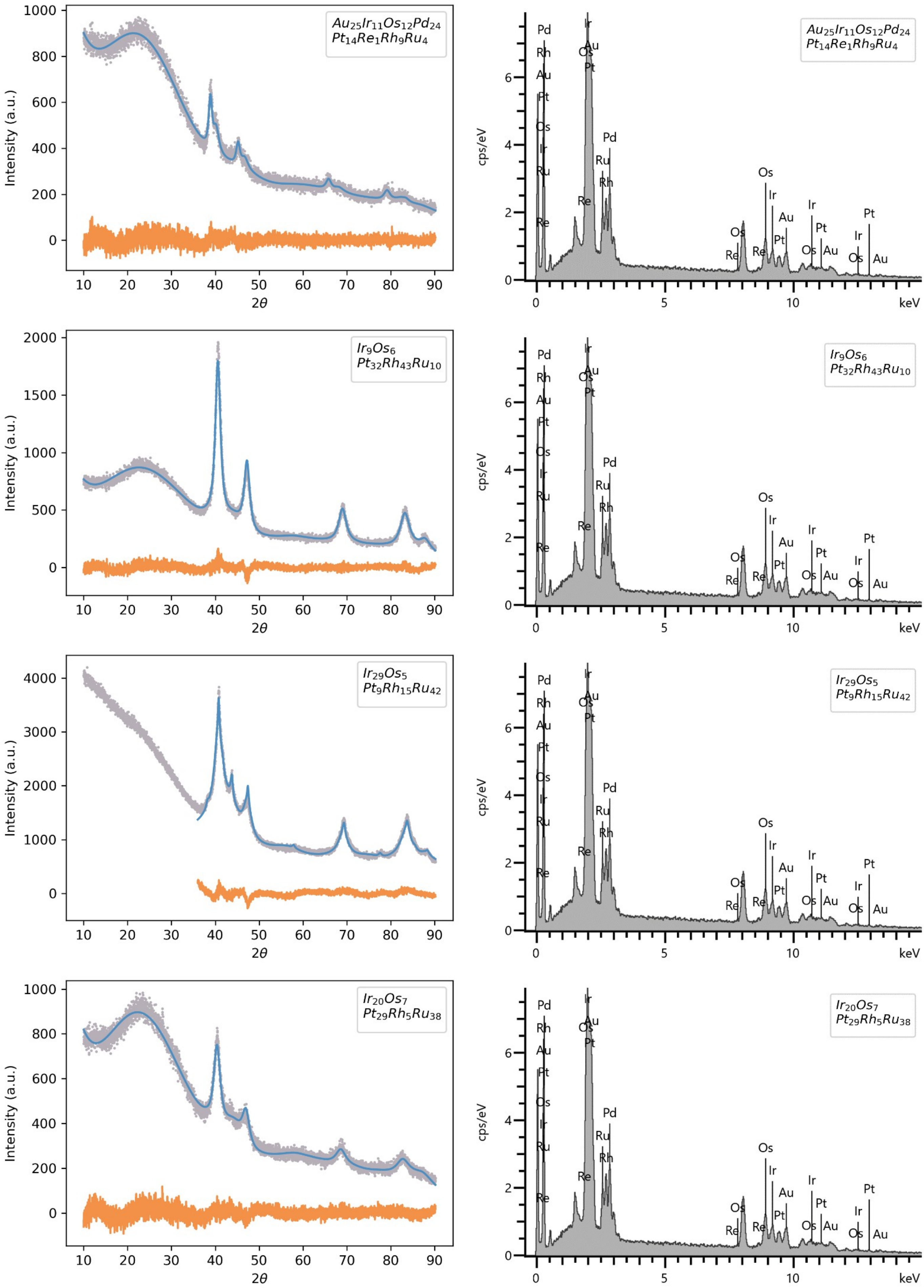

Intensity (a.u.)
2θ
cps/eV
keV
$Au_{25}Ir_{11}Os_{12}Pd_{24}$ $Pt_{14}Re_{1}Rh_{9}Ru_{4}$
$Ir_{9}Os_{6}$ $Pt_{32}Rh_{43}Ru_{10}$
$Ir_{29}Os_{5}$ $Pt_{9}Rh_{15}Ru_{42}$
$Ir_{20}Os_{7}$ $Pt_{29}Rh_{5}Ru_{38}$
Pd
Rh
Au
Pt
Os
Ir
Ru
Re

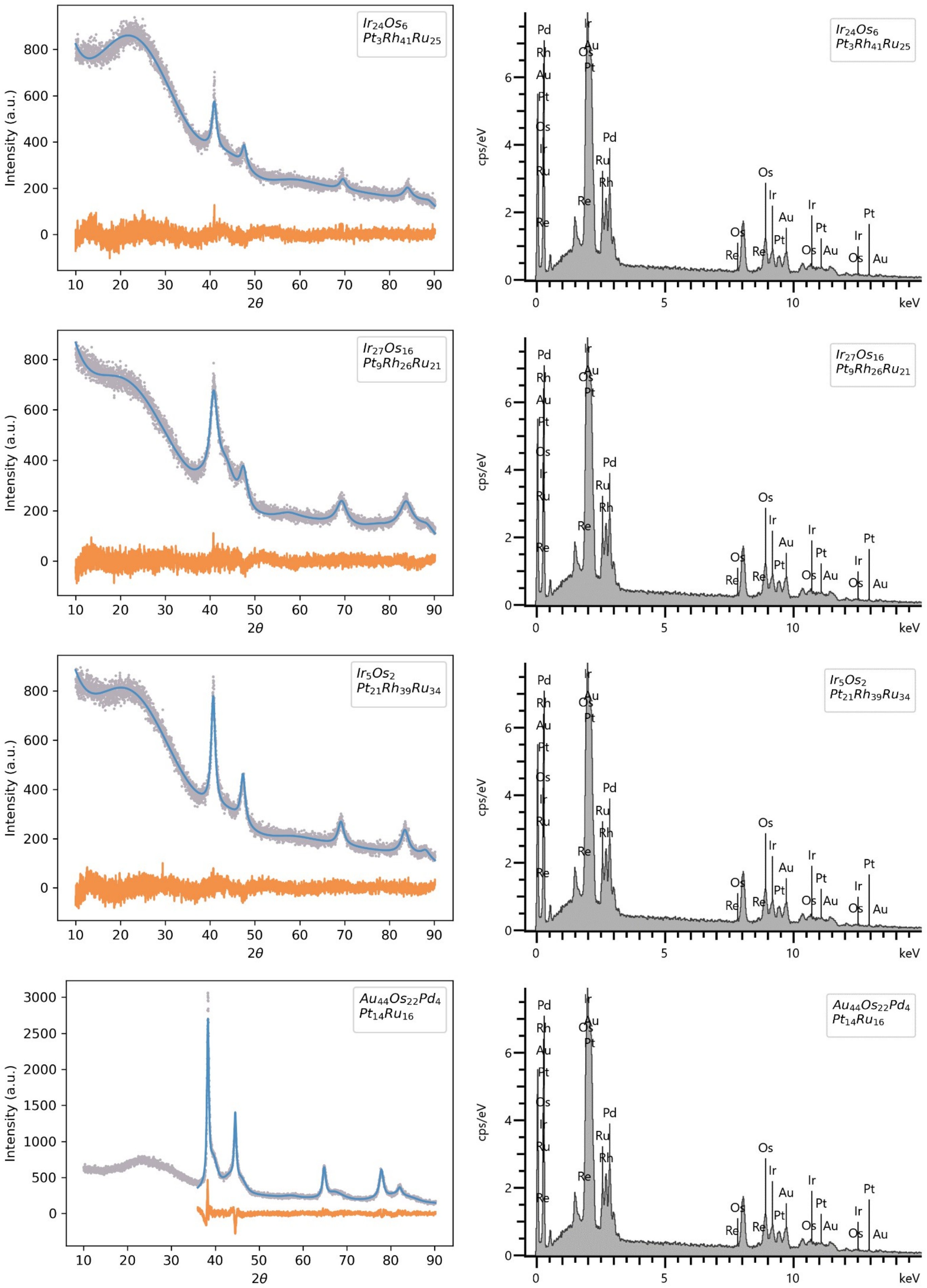
Intensity (a.u.)
2θ
cps/eV
keV
Ir24Os6
Pt3Rh41Ru25
Ir27Os16
Pt9Rh26Ru21
Ir5Os2
Pt21Rh39Ru34
Au44Os22Pd4
Pt14Ru16

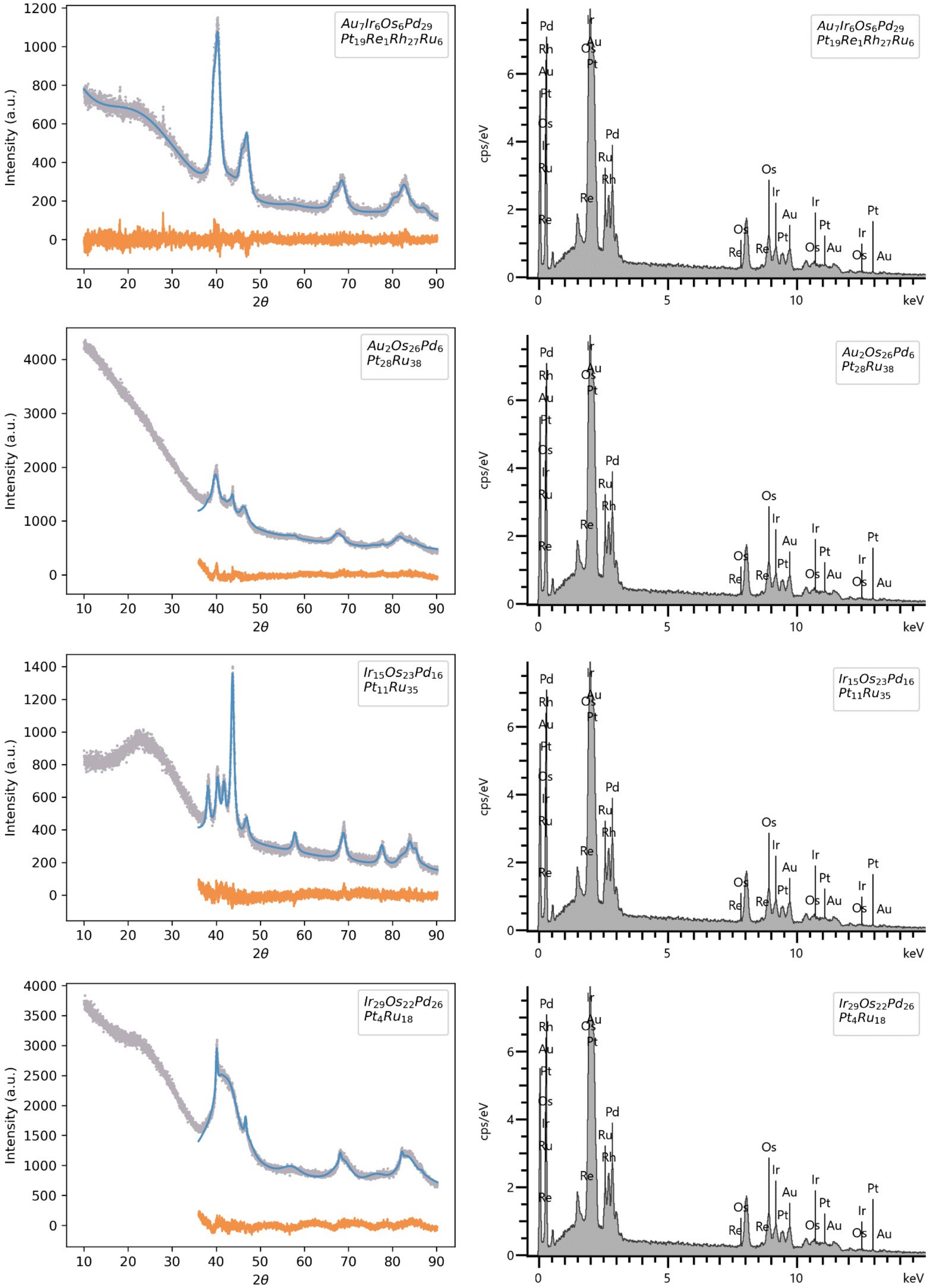

$Au_7Ir_6Os_6Pd_{29}$ $Pt_{19}Re_1Rh_{27}Ru_6$
$Au_2Os_{26}Pd_6$ $Pt_{28}Ru_{38}$
$Ir_{15}Os_{23}Pd_{16}$ $Pt_{11}Ru_{35}$
$Ir_{29}Os_{22}Pd_{26}$ Pt_4Ru_{18}
Intensity (a.u.)
2θ
cps/eV
keV
Pd
Rh
Au
Pt
Os
Ir
Ru
Re

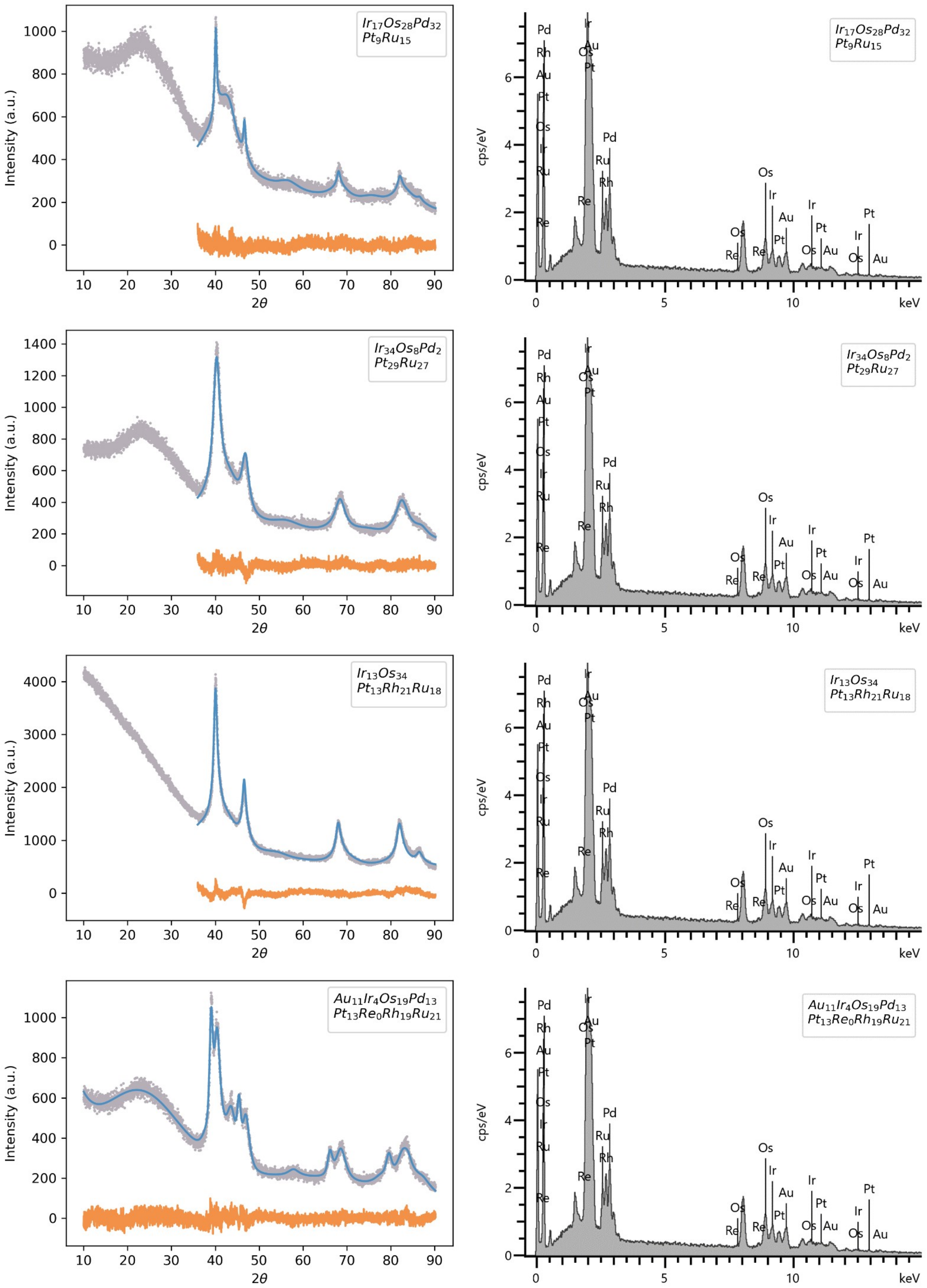

$Ir_{17}Os_{28}Pd_{32}$
$Pt_{9}Ru_{15}$
$Ir_{34}Os_{8}Pd_{2}$
$Pt_{29}Ru_{27}$
$Ir_{13}Os_{34}$
$Pt_{13}Rh_{21}Ru_{18}$
$Au_{11}Ir_{4}Os_{19}Pd_{13}$
$Pt_{13}Re_{0}Rh_{19}Ru_{21}$
Intensity (a.u.)
2θ
cps/eV
keV
Pd
Rh
Au
Pt
Os
Ir
Ru
Re

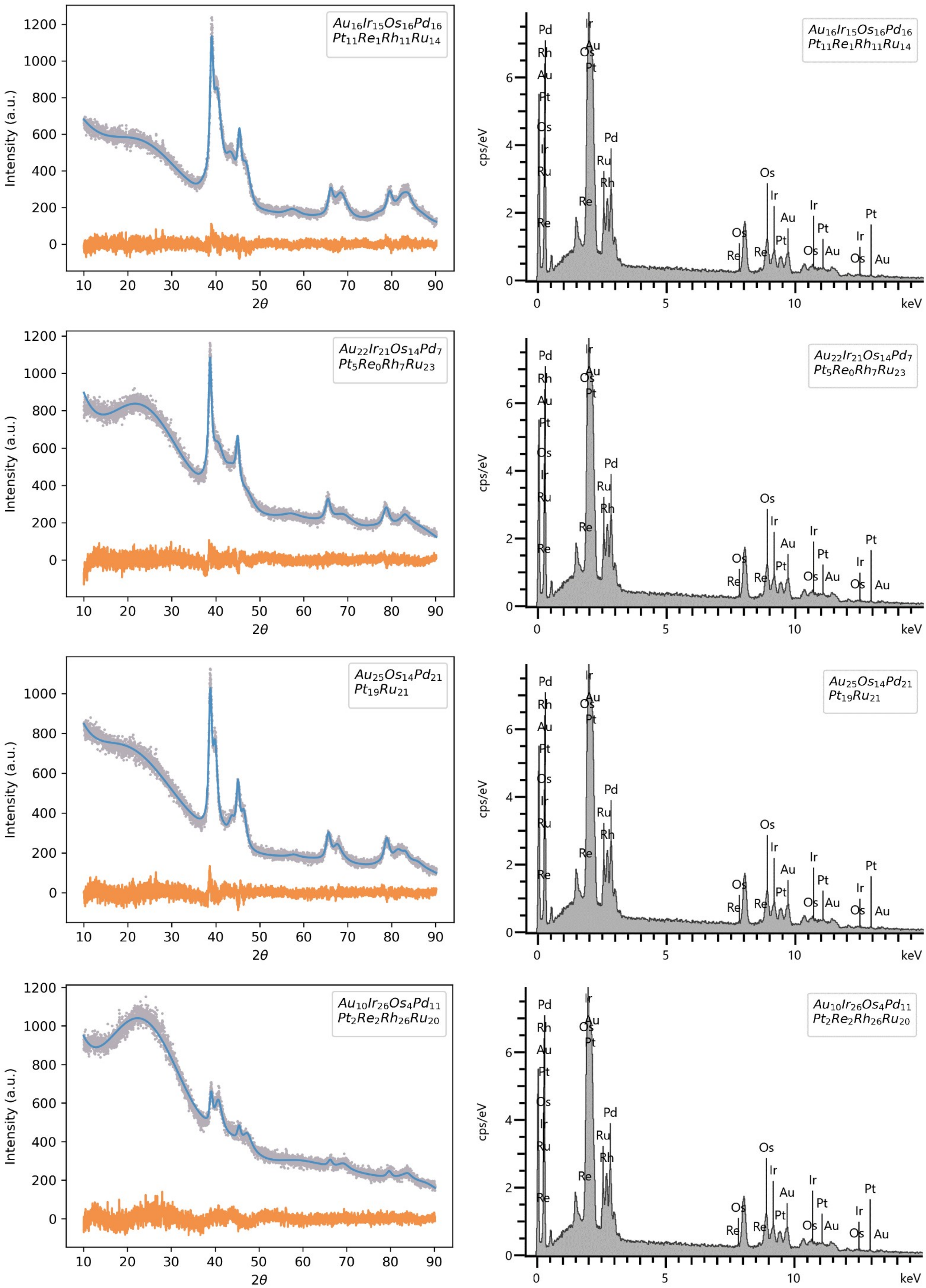
$Au_{16}Ir_{15}Os_{16}Pd_{16}$ $Pt_{11}Re_{1}Rh_{11}Ru_{14}$
$Au_{22}Ir_{21}Os_{14}Pd_{7}$ $Pt_{5}Re_{0}Rh_{7}Ru_{23}$
$Au_{25}Os_{14}Pd_{21}$ $Pt_{19}Ru_{21}$
$Au_{10}Ir_{26}Os_{4}Pd_{11}$ $Pt_{2}Re_{2}Rh_{26}Ru_{20}$
Intensity (a.u.)
2θ
cps/eV
keV
Pd
Rh
Au
Pt
Os
Ir
Ru
Re

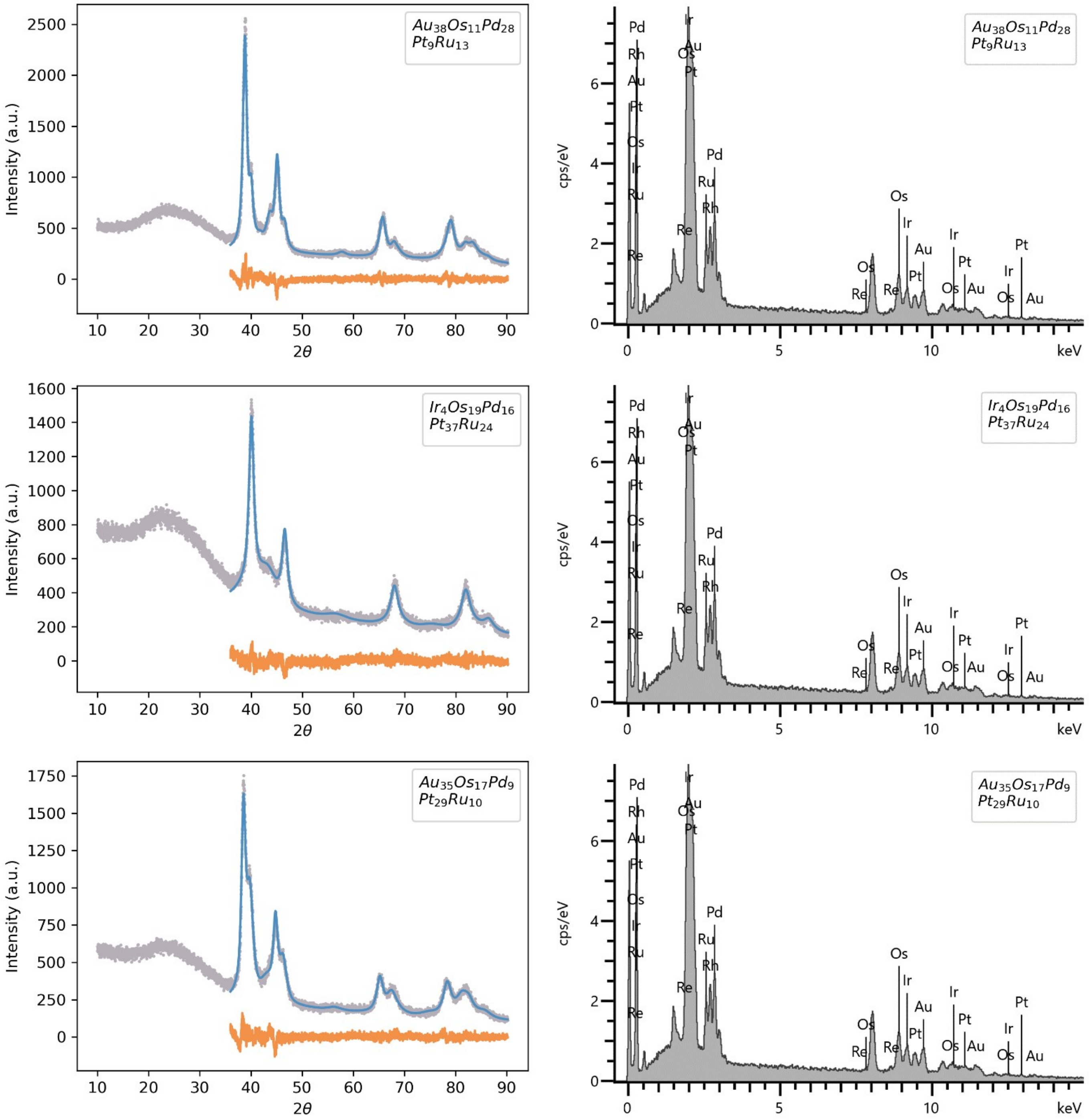

Fig S4. XRD measurements. Complementary XRD measurements of selected nanoparticles (left side) and their EDX spectra (right side). In the XRD figures the grey line corresponds to the measured data, the blue line to the fitted data and the orange to the difference. The EDX spectrum is composed of 4 stacked EDX spectra recorded at 4 different locations. The unaccounted peaks at 1.49 and 8.04 keV correspond to the Al holder and Cu tape respectively.

Table S1. Phase composition of the different samples.

Sample	fcc1	fcc2	hcp1	hcp2	Comments
$Au_9Os_{18}Pd_{21}Pt_{41}Ru_{11}$	y	y	(n)	n	Two fcc: One for broad peaks, one for narrower peaks. Not clear if hcp is present.
$Ir_{28}Os_3Pd_{32}Pt_{32}Ru_5$	y	y	(n)	n	Two fcc : Similar lattice params., different crystallite sizes
$Au_2Ir_{20}Os_6Pd_{18}Pt_{23}Re_3Rh_2Ru_{24}$	y	y	y	n	Two fcc: One for broad peaks, one for narrower peaks. One hcp for broader features.
$Ir_{25}Os_{11}Pd_{11}Pt_{47}Ru_7$	y	n	(n)	n	One fcc to describe peaks. Probably some hcp in there, but hard to fit.
$Ir_{27}Os_5Pd_{41}Pt_{17}Ru_{11}$	y	y	(y)	n	Two fcc describe peaks of equal lattice parameters but different crystallite sizes. Maybe an hcp for broader features.
$Au_{38}Ir_{26}Os_6Pd_3Pt_5Re_2Rh_{10}Ru_{10}$	y	y	y	n	Two fccs: One for broad peaks, one for narrower peaks. One hcp for very broad features.
$Au_{22}Ir_4Os_{19}Pd_8Pt_9Re_1Rh_{25}Ru_{12}$	y	y	y	n	Two fccs: One for broad peaks, one for narrower peaks. One hcp for broader features.
$Ir_{23}Os_{10}Pd_{24}Pt_{25}Ru_{18}$	y	n	y	n	One fcc describes visible peaks. One hcp describes broader features.
$Au_{25}Ir_{11}Os_{12}Pd_{24}Pt_{14}Re_1Rh_9Ru_4$	y	y	(y)	n	Two fcc with similar width but different intensities and position. One hcp for very broad features.
$Ir_9Os_6Pt_{32}Rh_{43}Ru_{10}$	y	n	y	n	One fcc describes visible peaks. One hcp describes broader features.
$Ir_{29}Os_5Pt_9Rh_{15}Ru_{42}$	y	y	y	y	Two fcc: Similar lattice parameters but different widths. Two hcps: one for broad features, one for very sharp peaks.
$Ir_{20}Os_7Pt_{29}Rh_5Ru_{38}$	y	n	y	n	One fcc describes visible peaks. One hcp describes broader features.
$Ir_{24}Os_6Pt_3Rh_{41}Ru_{25}$	y	n	y	n	One fcc describes visible peaks. One hcp describes broader features. Fit w. two fcc rather than fcc+hcp almost identical.
$Ir_{27}Os_{16}Pt_9Rh_{26}Ru_{21}$	y	n	y	n	One fcc describes visible peaks. One hcp describes broader features.
$Ir_5Os_2Pt_{21}Rh_{39}Ru_{34}$	y	n	y	n	One fcc describes visible peaks. One hcp describes broader features.
$Au_{44}Os_{22}Pd_4Pt_{14}Ru_{16}$	y	y	y	n	Two fcc: One for broad peaks, one for narrower peaks. hcp for broader features.
$Au_7Ir_6Os_6Pd_{29}Pt_{19}Re_1Rh_{27}Ru_6$	y	y	y	n	Two fcc with similar width but different intensities and peak positions. One hcp for broad features.
$Au_2Os_{26}Pd_6Pt_{28}Ru_{38}$	y	n	y	y	One fcc and hcp to describe broad features. An additional hcp to describe small, sharp peaks
$Ir_{15}Os_{23}Pd_{16}Pt_{11}Ru_{35}$	y	(n)	y	n	One fcc and one hcp describe peaks of similar width and intensity.
$Ir_{29}Os_{22}Pd_{26}Pt_4Ru_{18}$	y	y	y	n	One fcc and hcp to describe broad features. An additional fcc to describe small, sharp peaks
$Ir_{17}Os_{28}Pd_{32}Pt_9Ru_{15}$	y	y	y	n	Two fcc, one for broader and one for narrower peaks of similar positions. hcp for broader features.
$Ir_{34}Os_8Pd_2Pt_{29}Ru_{27}$	y	n	y	n	One fcc describes visible peaks. One hcp describes broader features.
$Ir_{13}Os_{34}Pt_{13}Rh_{21}Ru_{18}$	y	y	y	n	Two fcc: Similar lattice parameters but different widths. An hcp to describe remaining broad features.
$Au_{11}Ir_4Os_{19}Pd_{13}Pt_{13}Re_0Rh_{19}Ru_{21}$	y	y	y	n	Two fcc with similar width but different intensities and position. One hcp for broader features.
$Au_{16}Ir_{15}Os_{16}Pd_{16}Pt_{11}Re_1Rh_{11}Ru_{14}$	y	y	y	n	Two fcc: One for broad peaks, one for narrower peaks. An hcp for very broad features.
$Au_{22}Ir_{21}Os_{14}Pd_7Pt_5Re_0Rh_7Ru_{23}$	y	y	y	n	Two fcc: One for broad peaks, one for narrower peaks. One hcp for very broad features.
$Au_{25}Os_{14}Pd_{21}Pt_{19}Ru_{21}$	y	y	y	n	Two fccs with similar width and intensities but different positions. One hcp for broader features.
$Au_{10}Ir_{26}Os_4Pd_{11}Pt_2Re_2Rh_{26}Ru_{20}$	y	y	(y)	n	Two fcc with similar width but different intensities and position. One hcp for very broad features.
$Au_{38}Os_{11}Pd_{28}Pt_9Ru_{13}$	y	y	y	n	Two fcc with similar width but different intensity. hcp for less pronounced peaks.
$Ir_4Os_{19}Pd_{16}Pt_{37}Ru_{24}$	y	n	y	n	One fcc describes visible peaks. One hcp describes broader features.
$Au_{35}Os_{17}Pd_9Pt_{29}Ru_{10}$	y	y	y	n	Two fcc: One for broad peaks, one for narrower peaks. hcp for broader features.

Table S2. Refined phase contributions.

Sample	% fcc1	% fcc1 error	% fcc2	% fcc2 error	%3	%3_err	%4	%4_err
$Au_9Os_{18}Pd_{21}Pt_{41}Ru_{11}$	38.4	1.9	61.6	1.9				
$Ir_{28}Os_3Pd_{32}Pt_{32}Ru_5$	77.4	1.0	22.6	1.0				
$Au_2Ir_{20}Os_6Pd_{18}Pt_{23}Re_3Rh_2Ru_{24}$	28.7	2.6	30.4	2.9	40.9	2.7		
$Ir_{25}Os_{11}Pd_{11}Pt_{47}Ru_7$	100	0						
$Ir_{27}Os_5Pd_{41}Pt_{17}Ru_{11}$	27.1	2.1	28.6	2.0	44.3	2.6		
$Au_{38}Ir_{26}Os_6Pd_3Pt_5Re_2Rh_{10}Ru_{10}$	26.6	1.5	45.1	2.4	28.3	3.2		
$Au_{22}Ir_4Os_{19}Pd_8Pt_9Re_1Rh_{25}Ru_{12}$	13.9	2.0	28.4	4.3	57.7	5.8		
$Ir_{23}Os_{10}Pd_{24}Pt_{25}Ru_{18}$	42.8	1.3			57.2	1.3		
$Au_{25}Ir_{11}Os_{12}Pd_{24}Pt_{14}Re_1Rh_9Ru_4$	15.1	4.4	16.9	5.2	68.0	9.4		
$Ir_9Os_6Pt_{32}Rh_{43}Ru_{10}$	65.1	2.9			34.9	2.9		
$Ir_{29}Os_5Pt_9Rh_{15}Ru_{42}$	42.2	1.4	3.1	0.6	48.1	1.6	6.6	0.6
$Ir_{20}Os_7Pt_{29}Rh_5Ru_{38}$	25.9	2.8			74.1	2.8		
$Ir_{24}Os_6Pt_3Rh_{41}Ru_{25}$	18.9	2.5			81.1	2.5		
$Ir_{27}Os_{16}Pt_9Rh_{26}Ru_{21}$	30.9	2.2			69.1	2.2		
$Ir_5Os_2Pt_{21}Rh_{39}Ru_{34}$	21.4	3.4			78.6	3.4		
$Au_{44}Os_{22}Pd_4Pt_{14}Ru_{16}$	32.6	1.1	41.4	1.0	26.3	1.5		
$Au_7Ir_6Os_6Pd_{29}Pt_{19}Re_1Rh_{27}Ru_6$	53.9	2.3	20.0	1.1	26.1	3.0		
$Au_2Os_{26}Pd_6Pt_{28}Ru_{38}$	49.8	4.0			12.8	4.3	37.4	4.4
$Ir_{15}Os_{23}Pd_{16}Pt_{11}Ru_{35}$	18.0	0.4			82.0	0.4		
$Ir_{29}Os_{22}Pd_{26}Pt_4Ru_{18}$	39.7	1.6	2.8	0.3	57.5	1.6		
$Ir_{17}Os_{28}Pd_{32}Pt_9Ru_{15}$	18.4	2.1	8.4	0.9	73.2	2.2		
$Ir_{34}Os_8Pd_2Pt_{29}Ru_{27}$	54.2	1.6			45.8	1.6		
$Ir_{13}Os_{34}Pt_{13}Rh_{21}Ru_{18}$	46.3	2.6	11.2	2.3	42.5	2.6		
$Au_{11}Ir_4Os_{19}Pd_{13}Pt_{13}Re_0Rh_{19}Ru_{21}$	16.6	0.4	39.0	0.8	44.3	1.0		
$Au_{16}Ir_{15}Os_{16}Pd_{16}Pt_{11}Re_1Rh_{11}Ru_{14}$	41.0	1.0	20.4	0.6	38.6	1.2		
$Au_{22}Ir_{21}Os_{14}Pd_7Pt_5Re_0Rh_7Ru_{23}$	18.2	1.2	28.8	2.2	52.9	2.8		
$Au_{25}Os_{14}Pd_{21}Pt_{19}Ru_{21}$	40.2	1.1	30.0	0.8	29.7	1.4		
$Au_{10}Ir_{26}Os_4Pd_{11}Pt_2Re_2Rh_{26}Ru_{20}$	6.7	1.2	24.0	4.2	69.3	5.1		
$Au_{38}Os_{11}Pd_{28}Pt_9Ru_{13}$	54.6	0.6	17.1	0.5	28.3	0.7		
$Ir_4Os_{19}Pd_{16}Pt_{37}Ru_{24}$	53.0	1.4			47.0	1.4		
$Au_{35}Os_{17}Pd_9Pt_{29}Ru_{10}$	37.6	0.8	43.4	0.8	19.0	1.3		

Table S3. Refined lattice parameters

Sample	Rwp / %	a fcc1 / Å	a fcc1 error / Å	a fcc2 / Å	a fcc2 error /Å	a hcp1 / Å	a hcp1 error / Å	c hcp1 / Å	c hcp1 error / Å	a hcp2 / Å	a hcp2 error / Å	c hcp2 / Å	c hcp2 error / Å
$Au_{9}Os_{18}Pd_{21}Pt_{41}Ru_{11}$	6.272	3.9098	0.0004	3.9655	0.0042								
$Ir_{28}Os_{3}Pd_{32}Pt_{32}Ru_{5}$	8.3	3.8836	0	3.898	0								
$Au_{2}Ir_{20}Os_{6}Pd_{18}Pt_{23}Re_{3}Rh_{2}Ru_{24}$	4.486	3.9087	0.0006	3.8476	0.0069	2.7097	0.0024	4.3934	0.0089				
$Ir_{25}Os_{11}Pd_{11}Pt_{47}Ru_{7}$	6.568	3.8972	0.0003										
$Ir_{27}Os_{5}Pd_{41}Pt_{17}Ru_{11}$	5.728	3.8641	0.003	3.8957	0.0004	2.7727	0.0055	4.6394	0.0164				
$Au_{38}Ir_{26}Os_{6}Pd_{3}Pt_{5}Re_{2}Rh_{10}Ru_{10}$	5.088	4.0538	0.0004	3.8824	0.0028	2.7183	0.0045	4.3695	0.021				
$Au_{22}Ir_{4}Os_{19}Pd_{8}Pt_{9}Re_{1}Rh_{25}Ru_{12}$	4.272	4.0291	0.0007	3.8694	0.0032	2.6807	0.0071	4.3866	0.0199				
$Ir_{23}Os_{10}Pd_{24}Pt_{25}Ru_{18}$	6.199	3.8897	0.0003			2.7522	0.0032	4.6439	0.0116				
$Au_{25}Ir_{11}Os_{12}Pd_{24}Pt_{14}Re_{1}Rh_{9}Ru_{4}$	4.474	4.0113	0.0009	3.8884	0.0029	2.5465	0.0654	4.3476	0.2247				
$Ir_{9}Os_{6}Pt_{32}Rh_{43}Ru_{10}$	4.815	3.8485	0.0002			2.6649	0.0044	4.4025	0.0146				
$Ir_{29}Os_{5}Pt_{9}Rh_{15}Ru_{42}$	4.531	3.8364	0	3.8274	0	2.7337	0	4.4964	0	2.7147	0	4.3264	0
$Ir_{20}Os_{7}Pt_{29}Rh_{5}Ru_{38}$	4.815	3.8673	0.0008			2.6651	0.0052	4.3681	0.016				
$Ir_{24}Os_{6}Pt_{3}Rh_{41}Ru_{25}$	4.787	3.8174	0.0008			2.6023	0.0138	4.4121	0.0439				
$Ir_{27}Os_{16}Pt_{9}Rh_{26}Ru_{21}$	4.988	3.8302	0.0006			2.7231	0.0029	4.3594	0.0083				
$Ir_{5}Os_{2}Pt_{21}Rh_{39}Ru_{34}$	4.728	3.8393	0.0004			2.6194	0.0123	4.3802	0.0334				
$Au_{44}Os_{22}Pd_{4}Pt_{14}Ru_{16}$	6.788	3.9449	0.0021	4.0663	0.0001	2.7398	0.0028	4.3672	0.0103				
$Au_{7}Ir_{6}Os_{6}Pd_{29}Pt_{19}Re_{1}Rh_{27}Ru_{6}$	4.967	3.8708	0.0006	3.9646	0.001	2.7165	0.0043	4.4445	0.0149				
$Au_{2}Os_{26}Pd_{6}Pt_{28}Ru_{38}$	5.235	3.9143	0.0008			2.7232	0.0018	4.3127	0.0052	2.7624	0.0085	4.4103	0.0131
$Ir_{15}Os_{23}Pd_{16}Pt_{11}Ru_{35}$	6.243	3.8741	0.0007			2.7235	0.0002	4.3244	0.0007				
$Ir_{29}Os_{22}Pd_{26}Pt_{4}Ru_{18}$	4.107	3.8484	0.0015	3.8879	0.0004	2.7416	0.0011	4.3871	0.0032				
$Ir_{17}Os_{28}Pd_{32}Pt_{9}Ru_{15}$	5.496	3.8667	0.0046	3.8923	0.0005	2.7547	0.0019	4.434	0.0053				
$Ir_{34}Os_{8}Pd_{2}Pt_{29}Ru_{27}$	5.457	3.8752	0.0004			2.7454	0.002	4.5146	0.0065				
$Ir_{13}Os_{34}Pt_{13}Rh_{21}Ru_{18}$	4.67	3.8844	0.0011	3.8941	0.0008	2.715	0.0027	4.893	0.0118				
$Au_{11}Ir_{4}Os_{19}Pd_{13}Pt_{13}Re_{0}Rh_{19}Ru_{21}$	4.863	3.9935	0.0004	3.867	0.0006	2.7323	0.0012	4.31	0.0048				
$Au_{16}Ir_{15}Os_{16}Pd_{16}Pt_{11}Re_{1}Rh_{11}Ru_{14}$	4.937	3.8764	0.0011	3.9946	0.0004	2.7339	0.0012	4.3537	0.0049				
$Au_{22}Ir_{21}Os_{14}Pd_{7}Pt_{5}Re_{0}Rh_{7}Ru_{23}$	4.759	4.0274	0.0004	3.8583	0.0039	2.7083	0.0027	4.4304	0.01				
$Au_{25}Os_{14}Pd_{21}Pt_{19}Ru_{21}$	5.028	3.9158	0.0008	4.0219	0.0004	2.7233	0.0021	4.3316	0.0093				
$Au_{10}Ir_{26}Os_{4}Pd_{11}Pt_{2}Re_{2}Rh_{26}Ru_{20}$	4.44	3.9886	0.0011	3.835	0.002	2.5873	0.0285	4.3323	0.0717				
$Au_{38}Os_{11}Pd_{28}Pt_{9}Ru_{13}$	6.227	4.0163	0.0002	3.8995	0.0006	2.7274	0.0011	4.326	0.0039				
$Ir_{4}Os_{19}Pd_{16}Pt_{37}Ru_{24}$	5.796	3.8984	0.0002			2.7394	0.0019	4.461	0.0069				
$Au_{35}Os_{17}Pd_{9}Pt_{29}Ru_{10}$	6.147	3.9243	0.0007	4.0448	0.0003	2.736	0.0022	4.5084	0.0076				

Table S4. Refined crystallite sizes.

Sample	size, fcc1 / nm	size error, fcc1 / nm	size, fcc2 / nm	size error, fcc2 / nm	size, hcp1 / nm	size error, hcp1 /nm	size, hcp2 / nm	size error, hcp2 /nm
$Au_9Os_{18}Pd_{21}Pt_{41}Ru_{11}$	19.8	3.4	5.3	80.1				
$Ir_{28}Os_3Pd_{32}Pt_{32}Ru_5$	2.3	0.1	12.5	1.6				
$Au_2Ir_{20}Os_6Pd_{18}Pt_{23}Re_3Rh_2Ru_{24}$	19.0	3.0	7.4	1.6	3.7	1.2		
$Ir_{25}Os_{11}Pd_{11}Pt_{47}Ru_7$	7.3	18.6						
$Ir_{27}Os_5Pd_{41}Pt_{17}Ru_{11}$	6.5	1.0	25.1	4.4	1.5	0.3		
$Au_{38}Ir_{26}Os_6Pd_3Pt_5Re_2Rh_{10}Ru_{10}$	16.5	2.0	3.8	0.5	6.0	340.7		
$Au_{22}Ir_4Os_{19}Pd_8Pt_9Re_1Rh_{25}Ru_{12}$	22.0	6.4	7.4	18.8	2.1	43.7		
$Ir_{23}Os_{10}Pd_{24}Pt_{25}Ru_{18}$	12.5	33.6			1.8	0.3		
$Au_{25}Ir_{11}Os_{12}Pd_{24}Pt_{14}Re_1Rh_9Ru_4$	18.1	5.3	33.3	149.0	0.9	1.3		
$Ir_9Os_6Pt_{32}Rh_{43}Ru_{10}$	8.7	0.5			2.2	0.7		
$Ir_{29}Os_5Pt_9Rh_{15}Ru_{42}$	2.9	0.1	22.9	72.8	1.8	0.2	10.9	40.2
$Ir_{20}Os_7Pt_{29}Rh_5Ru_{38}$	10.0	1.8			1.4	25.8		
$Ir_{24}Os_6Pt_3Rh_{41}Ru_{25}$	8.8	52.7			1.2	0.5		
$Ir_{27}Os_{16}Pt_9Rh_{26}Ru_{21}$	3.8	13.1			2.0	0.7		
$Ir_5Os_2Pt_{21}Rh_{39}Ru_{34}$	11.5	32.8			0.8	0.3		
$Au_{44}Os_{22}Pd_4Pt_{14}Ru_{16}$	8.6	2.0	55.9	9.5	3.6	0.9		
$Au_7Ir_6Os_6Pd_{29}Pt_{19}Re_1Rh_{27}Ru_6$	11.2	0.9	23.4	7.2	3.0	1.7		
$Au_2Os_{26}Pd_6Pt_{28}Ru_{38}$	4.6	3.4			9.3	5.0	2.4	7.7
$Ir_{15}Os_{23}Pd_{16}Pt_{11}Ru_{35}$	4483.5	15100767.9			0.9	2.7		
$Ir_{29}Os_{22}Pd_{26}Pt_4Ru_{18}$	1.6	27.9	28.0	9.0	2.2	0.2		
$Ir_{17}Os_{28}Pd_{32}Pt_9Ru_{15}$	1.7	90.1	69.9	79.3	2.8	0.4		
$Ir_{34}Os_8Pd_2Pt_{29}Ru_{27}$	6.0	0.5			1.8	0.2		
$Ir_{13}Os_{34}Pt_{13}Rh_{21}Ru_{18}$	2.8	47.4	15.7	3.6	2.2	0.5		
$Au_{11}Ir_4Os_{19}Pd_{13}Pt_{13}Re_0Rh_{19}Ru_{21}$	15.7	1.6	7.4	0.6	3.5	0.5		
$Au_{16}Ir_{15}Os_{16}Pd_{16}Pt_{11}Re_1Rh_{11}Ru_{14}$	7.0	0.6	20.3	2.5	3.6	0.6		
$Au_{22}Ir_{21}Os_{14}Pd_7Pt_5Re_0Rh_7Ru_{23}$	28.0	6.3	3.3	0.6	3.1	81.2		
$Au_{25}Os_{14}Pd_{21}Pt_{19}Ru_{21}$	25.4	9.4	18.4	1.9	15.1	15.9		
$Au_{10}Ir_{26}Os_4Pd_{11}Pt_2Re_2Rh_{26}Ru_{20}$	16.3	5.8	6.9	3.4	1.2	1.1		
$Au_{38}Os_{11}Pd_{28}Pt_9Ru_{13}$	23.1	1.8	15.3	64.2	4.8	26.4		
$Ir_4Os_{19}Pd_{16}Pt_{37}Ru_{24}$	11.1	21.3			2.8	0.5		
$Au_{35}Os_{17}Pd_9Pt_{29}Ru_{10}$	10.9	1.1	22.8	2.3	3.6	0.8		

Table S5. Refined microstrain, as defined in Topas[3].

Sample	strain, fcc1	Strain error, fcc1	strain, fcc2	strain error, fcc2	strain, hcp1	strain error, hcp1	strain, hcp2	strain error, hcp2
$Au_9Os_{18}Pd_{21}Pt_{41}Ru_{11}$	1.65	0.09	5.00	0.36				
$Ir_{28}Os_3Pd_{32}Pt_{32}Ru_5$	0.00	0.14	1.03	0.10				
$Au_2Ir_{20}Os_6Pd_{18}Pt_{23}Re_3Rh_2Ru_{24}$	2.03	0.11	4.21	0.40	4.98	1.14		
$Ir_{25}Os_{11}Pd_{11}Pt_{47}Ru_7$	2.68	0.09						
$Ir_{27}Os_5Pd_{41}Pt_{17}Ru_{11}$	3.46	0.24	1.50	0.06	5.00	1.30		
$Au_{38}Ir_{26}Os_6Pd_3Pt_5Re_2Rh_{10}Ru_{10}$	1.57	0.10	5.00	0.50	5.00	1.98		
$Au_{22}Ir_4Os_{19}Pd_8Pt_9Re_1Rh_{25}Ru_{12}$	1.73	0.18	4.91	0.65	5.00	2.59		
$Ir_{23}Os_{10}Pd_{24}Pt_{25}Ru_{18}$	1.77	0.08			3.39	0.96		
$Au_{25}Ir_{11}Os_{12}Pd_{24}Pt_{14}Re_1Rh_9Ru_4$	1.67	0.21	4.12	0.70	0.00	29.71		
$Ir_9Os_6Pt_{32}Rh_{43}Ru_{10}$	1.89	0.05			5.00	1.96		
$Ir_{29}Os_5Pt_9Rh_{15}Ru_{42}$	0.00	0.12	0.09	0.13	0.00	0.49	0.00	0.24
$Ir_{20}Os_7Pt_{29}Rh_5Ru_{38}$	3.17	0.21			5.00	2.41		
$Ir_{24}Os_6Pt_3Rh_{41}Ru_{25}$	1.33	0.20			5.00	6.36		
$Ir_{27}Os_{16}Pt_9Rh_{26}Ru_{21}$	1.14	0.16			5.00	1.58		
$Ir_5Os_2Pt_{21}Rh_{39}Ru_{34}$	1.41	0.09			5.00	5.45		
$Au_{44}Os_{22}Pd_4Pt_{14}Ru_{16}$	4.97	0.38	1.23	0.04	2.07	0.58		
$Au_7Ir_6Os_6Pd_{29}Pt_{19}Re_1Rh_{27}Ru_6$	2.42	0.09	2.33	0.18	4.01	1.90		
$Au_2Os_{26}Pd_6Pt_{28}Ru_{38}$	1.85	0.24			0.28	0.41	0.00	1.08
$Ir_{15}Os_{23}Pd_{16}Pt_{11}Ru_{35}$	2.74	0.24			0.71	40.07		
$Ir_{29}Os_{22}Pd_{26}Pt_4Ru_{18}$	0.00	0.35	0.27	0.11	0.00	0.30		
$Ir_{17}Os_{28}Pd_{32}Pt_9Ru_{15}$	0.00	1.01	1.24	0.14	4.54	0.65		
$Ir_{34}Os_8Pd_2Pt_{29}Ru_{27}$	2.25	0.11			0.08	0.58		
$Ir_{13}Os_{34}Pt_{13}Rh_{21}Ru_{18}$	0.00	0.16	0.50	0.09	2.88	0.87		
$Au_{11}Ir_4Os_{19}Pd_{13}Pt_{13}Re_0Rh_{19}Ru_{21}$	1.33	0.09	2.67	0.12	1.79	0.35		
$Au_{16}Ir_{15}Os_{16}Pd_{16}Pt_{11}Re_1Rh_{11}Ru_{14}$	3.63	0.17	1.63	0.09	2.00	0.39		
$Au_{22}Ir_{21}Os_{14}Pd_7Pt_5Re_0Rh_7Ru_{23}$	1.90	0.10	5.00	0.71	5.00	1.38		
$Au_{25}Os_{14}Pd_{21}Pt_{19}Ru_{21}$	3.37	0.18	1.36	0.08	4.52	0.88		
$Au_{10}Ir_{26}Os_4Pd_{11}Pt_2Re_2Rh_{26}Ru_{20}$	1.07	0.26	3.48	0.50	4.91	14.80		
$Au_{38}Os_{11}Pd_{28}Pt_9Ru_{13}$	1.94	0.05	1.57	0.14	0.89	0.32		
$Ir_4Os_{19}Pd_{16}Pt_{37}Ru_{24}$	1.60	0.07			2.93	0.67		
$Au_{35}Os_{17}Pd_9Pt_{29}Ru_{10}$	2.88	0.12	2.06	0.06	1.00	0.00		

III. Materials and Methods

Machine learning

For each set of measurements for each of the four alloy systems (Au-Ir-Os-Pd-Pt-Re-Rh-Ru, Au-Os-Pd-Pt-Ru, Ir-Os-Pd-Pt-Ru, and Ir-Os-Pt-Rh-Ru) a Gaussian process regressor was trained on the Cartesian coordinates of the averaged EDX measured compositions and their corresponding ORR cathodic currents at 0.60 V vs. RHE. These currents were divided by the average capacitance in the region between 0.30 and 0.50 V vs. RHE and corrected for the current offset at 0.9 V vs. RHE. The Cartesian coordinates of the compositions were used to eliminate a redundant feature from the compositions (because compositions are constrained to sum to unity). The transformation of compositions into $m-1$ dimensional Cartesian coordinates in the composition space with m being the number of elements was done by converting the barycentric coordinates of a regular simplex with unit side length using the matrix product between the simplex vertices and the composition,

$$r = Vf$$

Here, **r** is the (m – 1)-dimensional Cartesian coordinates of the m-dimensional molar fractions in **f** (i.e. m = 8 for an eight-metal alloy), **V** is the ([m – 1] × m) matrix whose columns are the Cartesian coordinates of the m vertices of the (m – 1)-dimensional simplex. **V** was obtained by setting the first vertex at the origin (0, 0, ..., 0), the second vertex one unit of distance away at the Cartesian coordinate (1, 0, …, 0) (forming a straight line of unit length), the third vertex one unit away from each of the two first vertices at coordinate (1/2, $3^{½}$/2, 0, …, 0) (forming an equilateral triangle), etc. for m – 1 dimensions.

For the Gaussian process, the target currents per capacitance were shifted to a mean of zero and scaled to unit variance prior to training. For predictions this scaling and shift was reversed. A zero mean prior and a squared exponential kernel with fitted white noise (Eq. S6) was used for the Gaussian process regressors to model the correlation between experimental measurements,

$$k(r_i, r_j) = \exp\left(-\frac{(r_i - r_j)^{\mathrm{T}}(r_i - r_j)}{2l^2}\right) + \delta_{\mathrm{ij}}\alpha^2 \qquad \text{(Eq. S6)}$$

Here k is the kernel function, $\mathbf{r}_i$ and $\mathbf{r}_j$ are the Cartesian coordinates of the composition indexed i and j, ^{T} denotes the transpose vector, l is the fitted correlation length scale hyperparameter, δ_{ij} is the Kronecker delta which is unity when i and j are identical and zero for non-identical i and j. α is the standard deviation of the fitted white noise (independently and identically normally-distributed noise) hyperparameter.

The hyperparameters of the kernel function, l, and α, were chosen so that they would maximize the log marginal likelihood of the Gaussian process regressor[7.] Or put in other words, the hyperparameters were chosen to maximize the probability of the observed data given the Gaussian process regressor with the kernel in Eq. S6. Plots of the log marginal likelihoods as a function of l and α are shown in Fig. S5.

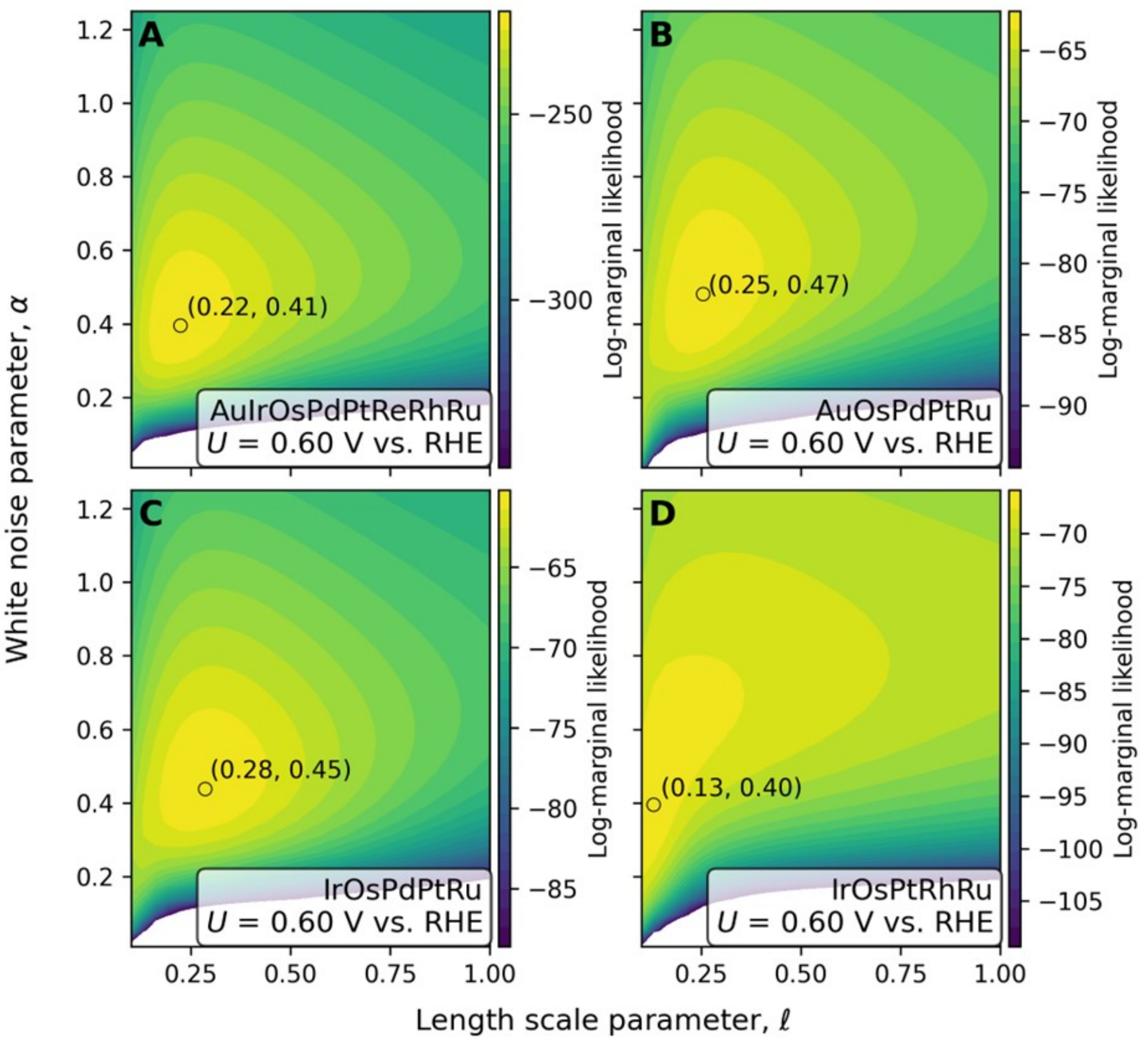

Fig. S5. Gaussian process regressor (GPR) hyperparameter optimization. (**A**-**D**) Log-marginal likelihood contour plots were used to optimize the two hyperparameters for the GPRs of the four alloy systems. Log-marginal likelihoods are shown as a function of the length scale, *l*, and the white noise, α, hyperparameters. The three alloy systems Au-Ir-Os-Pd-Pt-Re-Rh-Ru (A), Au-Os-Pd-Pt-Ru (B), and Ir-Os-Pd-Pt-Ru (C) show similar optimal length scales and white noises of ~0.25 and ~0.45, respectively, whereas Ir-Os-Pt-Rh-Ru (D) has a log-marginal likelihood optimum at a shorter length scale of 0.13. The Ir-Os-Pt-Rh-Ru system (D) shows a rather flat plateau of log-marginal likelihoods when going to length scales comparable to the other alloy systems, however, at the cost of higher white noise levels.

For the Bayesian optimization the maximum of the expected improvement acquisition function in Eq. S7 was used to evaluate the next point to sample.

$$E[I(r)] = \int_{-\infty}^{y_{\min}} (y_{\min} - y) N(y; \mu(r), \sigma(r)) dy$$

$$= (y_{\min} - \mu(r)) \Phi\left(\frac{y_{\min} - \mu(r)}{\sigma(r)}\right) + \sigma(r) \phi\left(\frac{y_{\min} - \mu(r)}{\sigma(r)}\right)$$

(Eq S7)

Here E is the expectation value, $I(\mathbf{r})$ the improvement function at a point $\mathbf{r}$, y_{min} is the lowest current sampled so far in the optimization, y is the current being integrated over, N is the normal distribution function, the mean of which, $\mu(\mathbf{r})$, is the Gaussian process predicted current at the point $\mathbf{r}$, and whose standard deviation $\sigma(\mathbf{r})$ is the uncertainty predicted by the Gaussian process at the same point, Φ is the cumulative distribution function of the standard normal distribution (i.e. with $\mu = 0$ and $\sigma = 1$), and ϕ is the standard normal distribution function.

Synthesis

The particles were synthesized using a solvothermal based synthesis[8]. The precursors, H_2PtCl_6 (Alfa Aesar, 99.999%), $HAuCl_3$ (Alfa Aesar, 99.99%), $RuCl_3$ (Sigma Aldrich, ReagentPlus), $RhCl_3$ (Alfa Aesar and Sigma Aldrich, 99.98%), $IrCl_3$ (Strem Chemicals, 99.9%), $OsCl_3$ (Sigma Aldrich), $ReCl_3$ (Sigma Aldrich), $PdCl_2$ (Sigma Aldrich, 99%) were dissolved in EtOH (VWR, AnalaR NORMAPUR ACS) to form 20 mM precursor solution. Following, a 1 ml stoichiometric mixture of these precursor solutions was added together with 3 ml EtOH to the microwave vessel. The stoichiometric mixtures were selected using a Sobol Sequence generator[9,10]. This produced a 5 mM reaction mixture. This mixture was heated up to reach a pressure of 20 bars for a duration of 30 minutes in the microwave (CEM, Discover SP). The resulting colloidal suspension was centrifuged, washed with EtOH, and air-dried. Following, it was dissolved in 3.25 ml water:iso-propanol (3:1) (Water: deionized and ultrafiltered by a Millipore MilliQ system; iso-propanol: VW) to produce the catalyst ink, with an assumed concentration of 6.15 mM metal. The energy-dispersive X-ray (EDX) spectra of the particles were measured on Cu tape using the EDS Photodetector Ultim max 65 (Oxford instruments) in a GeminiSEM450 (Zeiss). The spectra were taken at 4 different spots, each with a size of 588 μm^2 at a working distance of 8 mm and an accelerating voltage of 25 kV. Subsequently, the acquired spectra were analyzed using AZtec 5.0 (Oxford instruments).

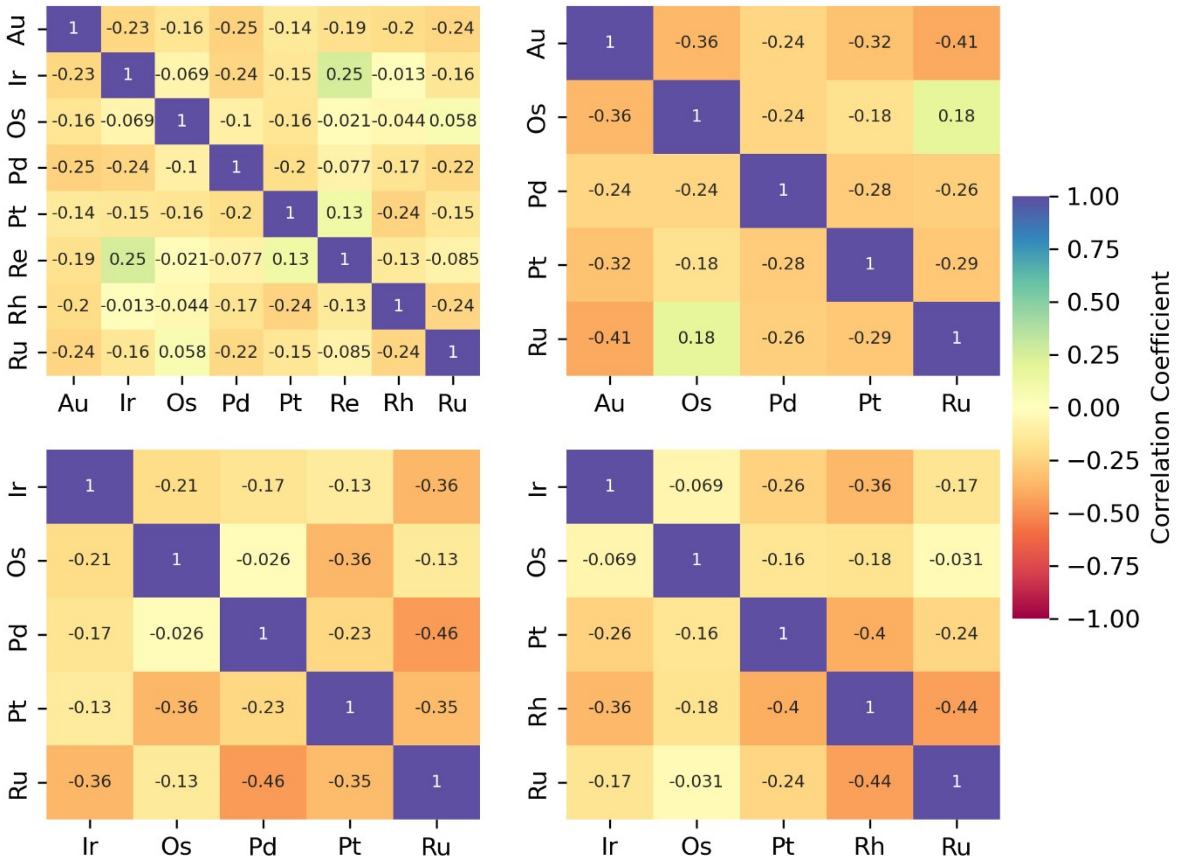

Fig. S6. Correlation matrixes of molar fractions. The correlation matrixes for each of the experimental spaces are composed of the Pearson correlation coefficients.

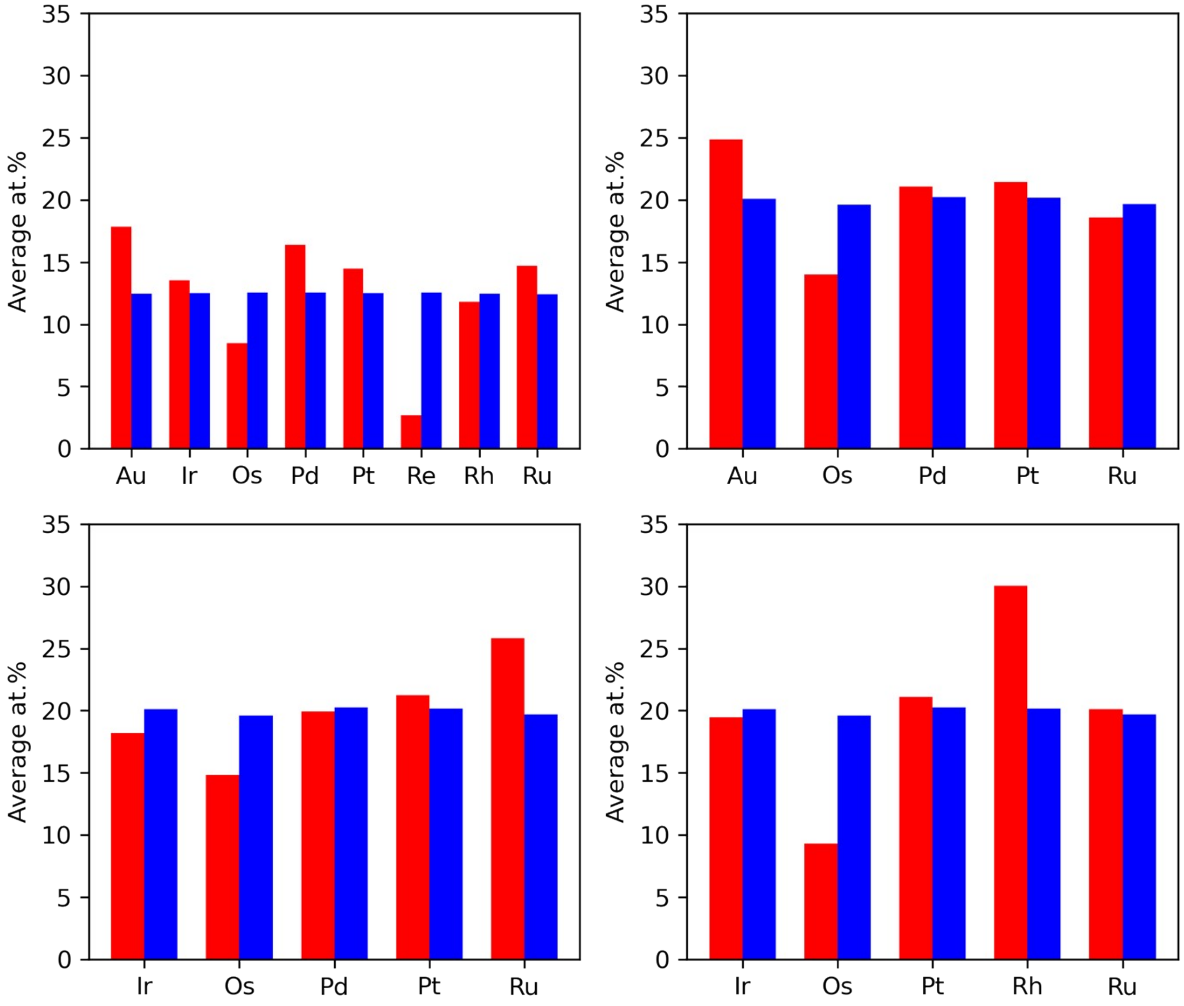

Fig. S7. Averaged location in composition space. (red) The average atomic presence of an element in the experimental samples within a catalytic space. (blue) The expected average atomic presence based on the precursor ratios during the synthesis.

Electrochemistry

Electrochemical experiments were conducted in a three-electrode cell utilizing a multi-working electrode (MWE) (Fig. S8). The MWE contained 6 glassy carbon disks, each of 5 mm in diameter. 8.181 ml of ink was deposited on each of the disk to produce a catalyst loading of 256 nmol cm^{-2}. The reference electrode constituted of a reversible hydrogen electrode. The counter electrode was a platinum wire, which was separated from the main compartment with a frit. The mass transport during the measurements was controlled using a magnetic steering rod, which rotated at 1500 revolutions per minute (RPM). All water used in these experiments was deionized and ultrafiltered by a Millipore MilliQ system (resistivity > 18.2 MΩ cm, TOC ≤ 5 ppb). The electrolyte used in the experiments was 0.1 M H_2SO_4 (Merck, Suprapur) and was renewed after each measurement.

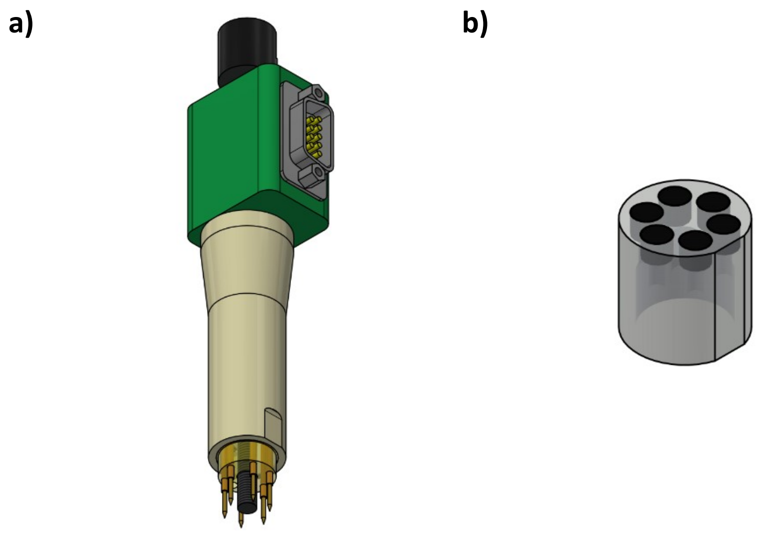

Fig S8. Electrode in the experimental setup. (**a**-**b**) A rendering of the multi-working-electrode shaft (a) and sample holder (b). The shaft fits to standards glass fittings for an air-tight fit to the electrolyte enclosure. The sample holder has 6 glassy carbon disks, onto which the different catalyst samples can be drop casted. The sample holder and the shaft are keyed so that each sample can easily be related to the acquired current signal.

First, 10 cyclic voltammograms were measured between 0.00 and 0.60 V vs. RHE at a scan rate of 100 mV s^{-1}. In the last cycle, the average capacitance was determined in the potential window from 0.3 to 0.5 V vs. RHE. Afterwards, the electrolyte was saturated with O_2 (Alphagaz, 4.5 purity grade) for 20 minutes while the electrode was kept at 1.00 V vs. RHE. Following, the potential was stepped down to 0.60 V vs. RHE in steps of 10 mV, which lasted for 20 seconds. The current was averaged over the last 17 seconds of each time step to produce a single data point.

IV. Capacitance measurements

The capacitance of the nanoparticles was determined using the current difference between the anodic and cathodic sweep in the potential window between 0.3 to 0.5 V vs. RHE. To demonstrate that mostly capacitive contributions are present in this potential window, the CVs of pure metals were measured. These measurements were carried out in 0.1 M H_2SO_4 at 250 mV/s. These CVs show that only Au has no Faradaic contribution in the utilized potential window. Pt, Ir, and PtRu show minor contributions from the hydrogen under potential depositions and the RuOx reduction. On top of that Pd and Rh, show very large Faradaic contributions leading to erroneous errors in capacitance when that window is taken.

To estimate the error introduced by the faradaic contributions, we performed the following analysis. The potential window between 0.3 to 0.5 V vs. RHE was segmented into parts of 250 mV. In each of these smaller windows, the capacitance was evaluated. From these 8 capacitances, the standard deviation and the average was obtained. The average was used to normalize the standard deviation to bring them to the same order of magnitude. Results for the single metals are shown in Figure S9. For Au a normalized standard deviation σC/μC of 0.072 indicating a relatively flat double layer. Ir, Pt, PtRu, Rh and Pd each containing faradaic processes in said window which is reflected in σC/μC values larger than 0.25. In comparison our nanomaterials almost all exhibit σC/μC values < 0.2 with the majority being even bellow 0.1. This indicates that the window between 0.3 to 0.5 V vs. RHE for most nano material samples is flat.

The only two samples with values σC/μC above 0.23 are samples 104, 428, and 384. Sample 104 contains large oscillatory noise waves from the instrument, which did not influence the catalytic measurement. On the other hand, samples 384 and 428 do contain some visible faradaic contributions. However, we expect that the error that arises from these three samples was smoothened out by the Gaussian Process fit.

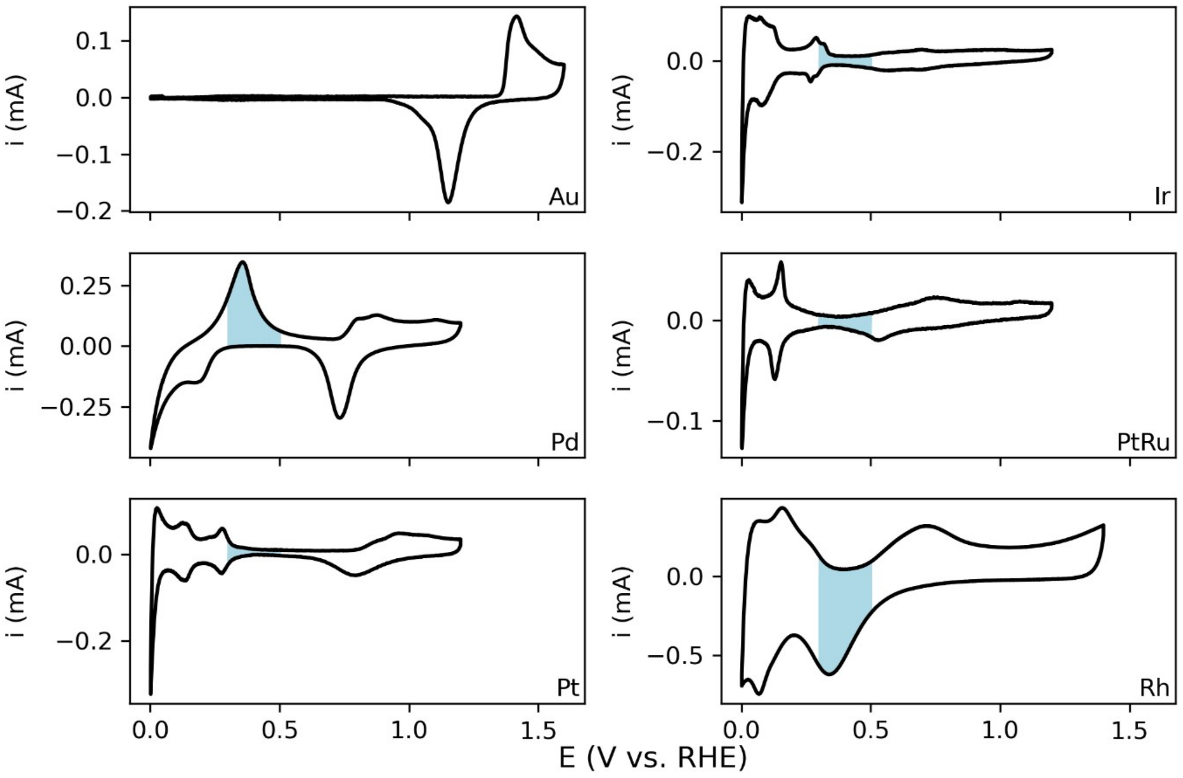

Fig S9: CVs of the individual metals in Ar. The measurements were recorded at 250 mV/s in 0.1 M H_2SO_4. The blue filled area is the area where the capacitance was determined for the nanomaterials.

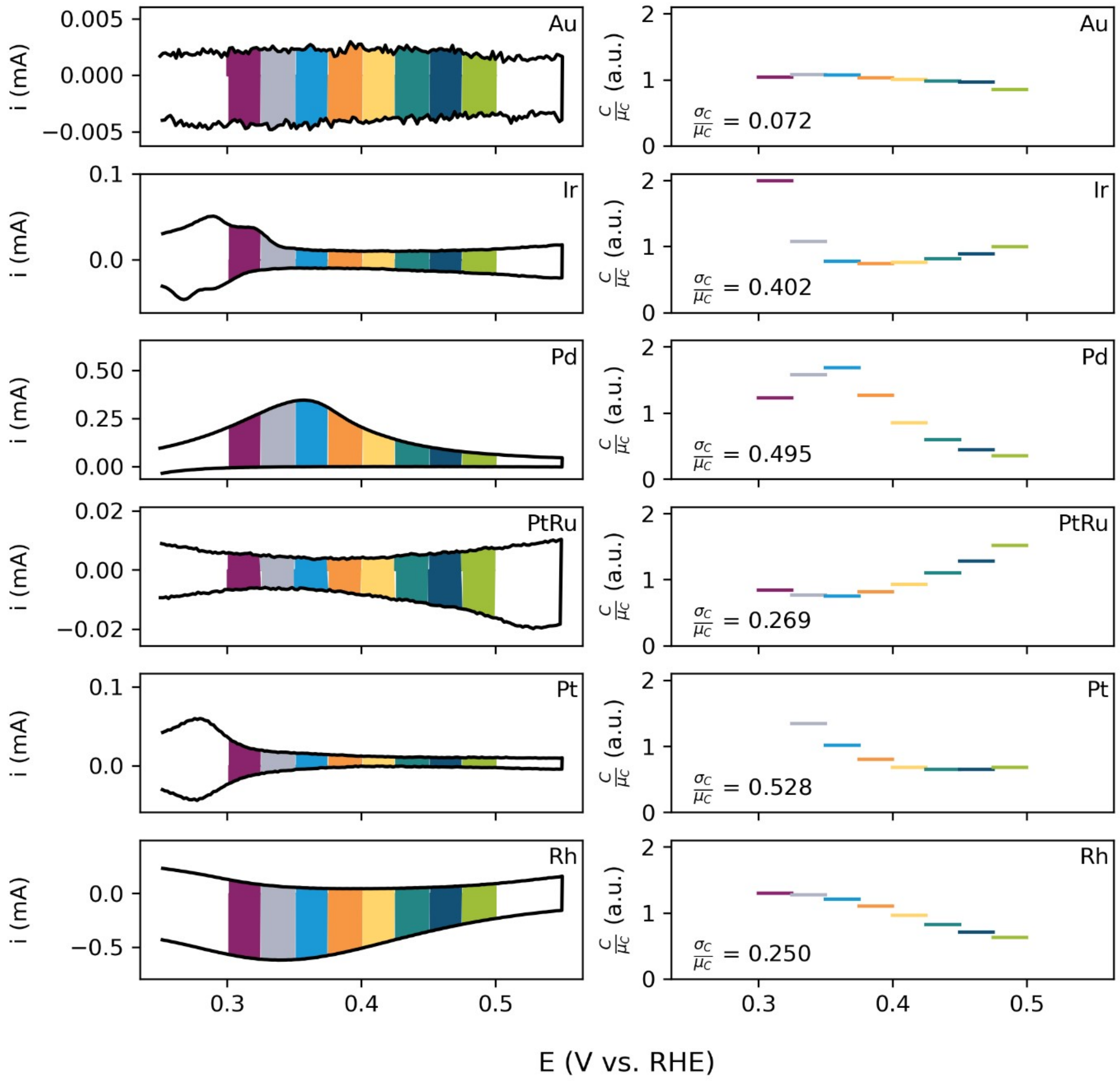

Fig S10. Capacitance measurements for the pure metals. The capacitance of the pure metals segmented over different potential windows.

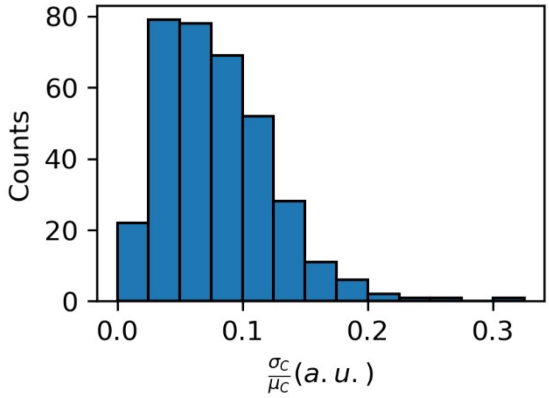

Fig S11. **Standard deviation for the determined capacitance**. The distribution of the standard deviation of the determined capacitance of the investigated nano materials. The standard deviation is normalized by the average capacitance of the investigated particle.

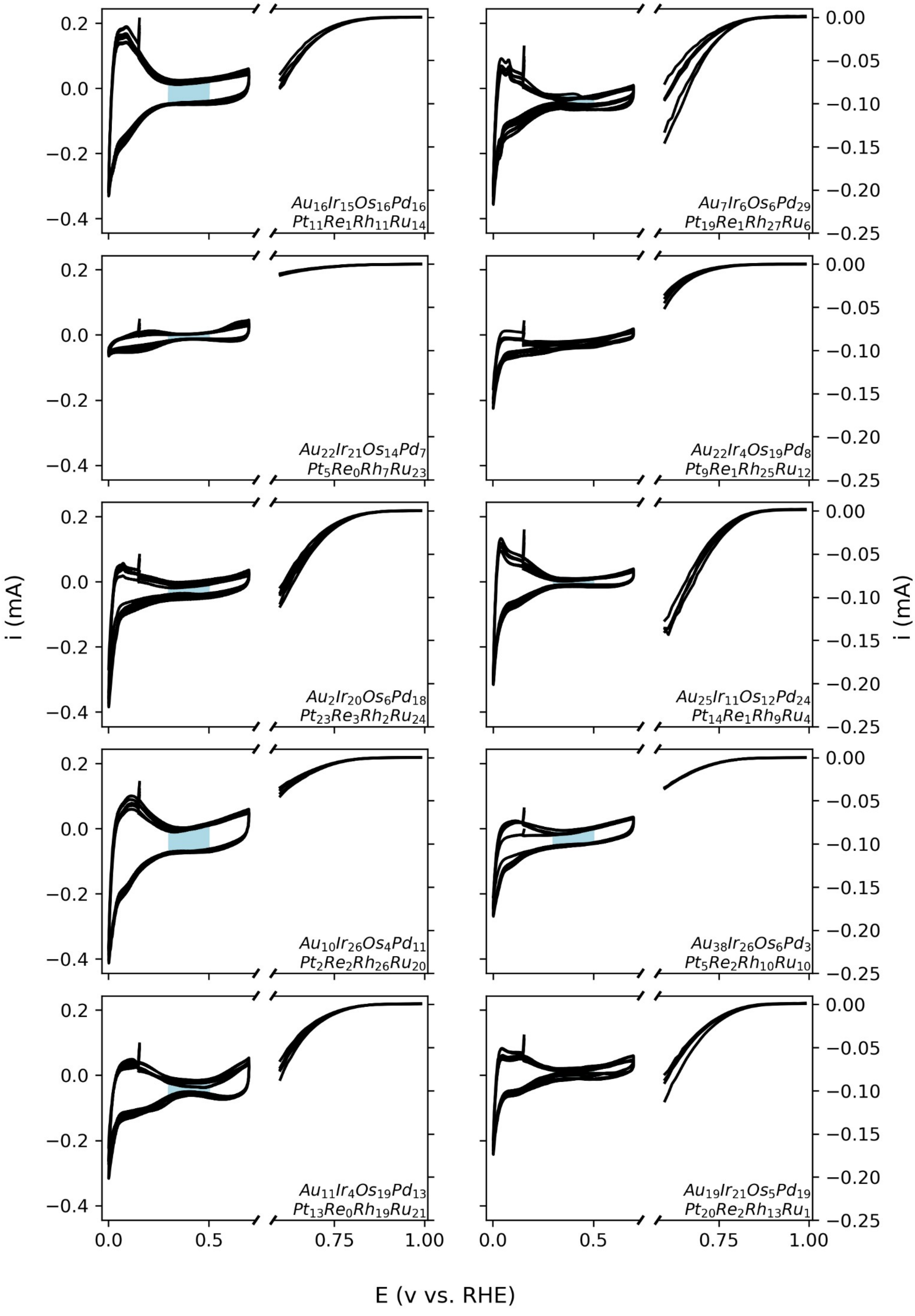
$Au_{16}Ir_{15}Os_{16}Pd_{16}$ $Pt_{11}Re_{1}Rh_{11}Ru_{14}$
$Au_{7}Ir_{6}Os_{6}Pd_{29}$ $Pt_{19}Re_{1}Rh_{27}Ru_{6}$
$Au_{22}Ir_{21}Os_{14}Pd_{7}$ $Pt_{5}Re_{0}Rh_{7}Ru_{23}$
$Au_{22}Ir_{4}Os_{19}Pd_{8}$ $Pt_{9}Re_{1}Rh_{25}Ru_{12}$
$Au_{2}Ir_{20}Os_{6}Pd_{18}$ $Pt_{23}Re_{3}Rh_{2}Ru_{24}$
$Au_{25}Ir_{11}Os_{12}Pd_{24}$ $Pt_{14}Re_{1}Rh_{9}Ru_{4}$
$Au_{10}Ir_{26}Os_{4}Pd_{11}$ $Pt_{2}Re_{2}Rh_{26}Ru_{20}$
$Au_{38}Ir_{26}Os_{6}Pd_{3}$ $Pt_{5}Re_{2}Rh_{10}Ru_{10}$
$Au_{11}Ir_{4}Os_{19}Pd_{13}$ $Pt_{13}Re_{0}Rh_{19}Ru_{21}$
$Au_{19}Ir_{21}Os_{5}Pd_{19}$ $Pt_{20}Re_{2}Rh_{13}Ru_{1}$
i (mA)
i (mA)
E (v vs. RHE)

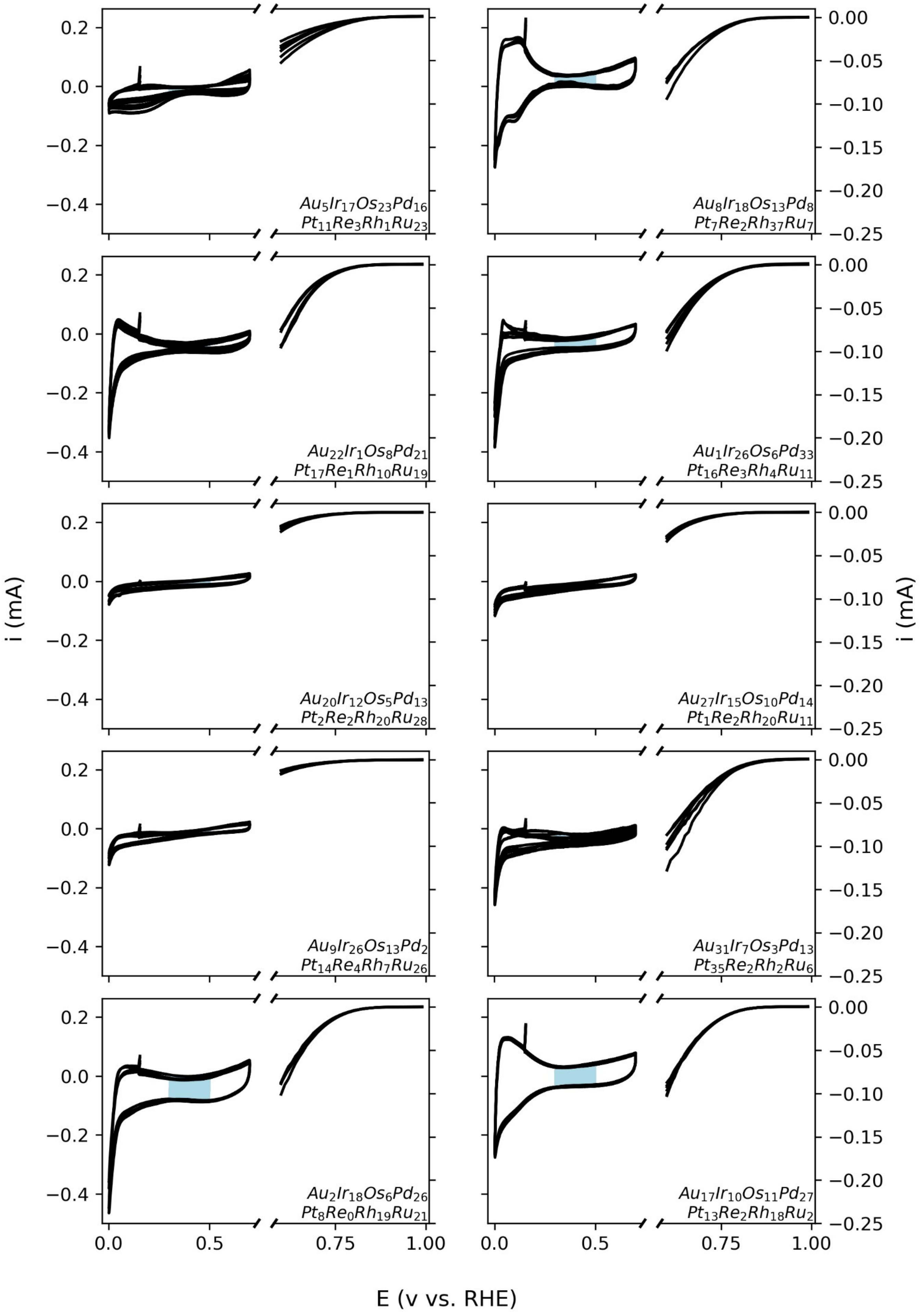
$Au_5Ir_{17}Os_{23}Pd_{16}$
$Pt_{11}Re_3Rh_1Ru_{23}$
$Au_8Ir_{18}Os_{13}Pd_8$
$Pt_7Re_2Rh_{37}Ru_7$
$Au_{22}Ir_1Os_8Pd_{21}$
$Pt_{17}Re_1Rh_{10}Ru_{19}$
$Au_1Ir_{26}Os_6Pd_{33}$
$Pt_{16}Re_3Rh_4Ru_{11}$
$Au_{20}Ir_{12}Os_5Pd_{13}$
$Pt_2Re_2Rh_{20}Ru_{28}$
$Au_{27}Ir_{15}Os_{10}Pd_{14}$
$Pt_1Re_2Rh_{20}Ru_{11}$
$Au_9Ir_{26}Os_{13}Pd_2$
$Pt_{14}Re_4Rh_7Ru_{26}$
$Au_{31}Ir_7Os_3Pd_{13}$
$Pt_{35}Re_2Rh_2Ru_6$
$Au_2Ir_{18}Os_6Pd_{26}$
$Pt_8Re_0Rh_{19}Ru_{21}$
$Au_{17}Ir_{10}Os_{11}Pd_{27}$
$Pt_{13}Re_2Rh_{18}Ru_2$
i (mA)
i (mA)
E (v vs. RHE)

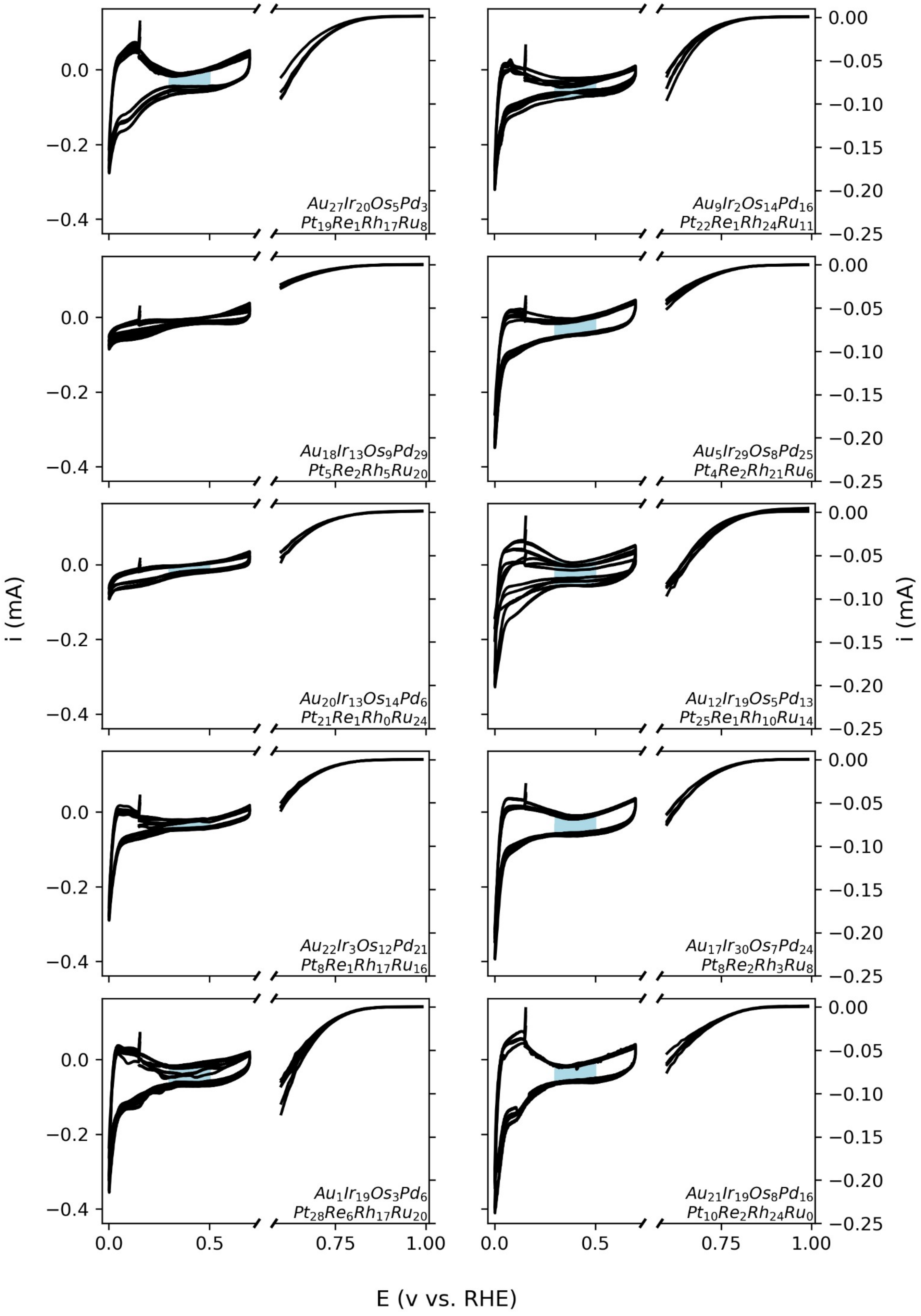

Au$_{27}$Ir$_{20}$Os$_{5}$Pd$_{3}$ Pt$_{19}$Re$_{1}$Rh$_{17}$Ru$_{8}$
Au$_{9}$Ir$_{2}$Os$_{14}$Pd$_{16}$ Pt$_{22}$Re$_{1}$Rh$_{24}$Ru$_{11}$
Au$_{18}$Ir$_{13}$Os$_{9}$Pd$_{29}$ Pt$_{5}$Re$_{2}$Rh$_{5}$Ru$_{20}$
Au$_{5}$Ir$_{29}$Os$_{8}$Pd$_{25}$ Pt$_{4}$Re$_{2}$Rh$_{21}$Ru$_{6}$
Au$_{20}$Ir$_{13}$Os$_{14}$Pd$_{6}$ Pt$_{21}$Re$_{1}$Rh$_{0}$Ru$_{24}$
Au$_{12}$Ir$_{19}$Os$_{5}$Pd$_{13}$ Pt$_{25}$Re$_{1}$Rh$_{10}$Ru$_{14}$
Au$_{22}$Ir$_{3}$Os$_{12}$Pd$_{21}$ Pt$_{8}$Re$_{1}$Rh$_{17}$Ru$_{16}$
Au$_{17}$Ir$_{30}$Os$_{7}$Pd$_{24}$ Pt$_{8}$Re$_{2}$Rh$_{3}$Ru$_{8}$
Au$_{1}$Ir$_{19}$Os$_{3}$Pd$_{6}$ Pt$_{28}$Re$_{6}$Rh$_{17}$Ru$_{20}$
Au$_{21}$Ir$_{19}$Os$_{8}$Pd$_{16}$ Pt$_{10}$Re$_{2}$Rh$_{24}$Ru$_{0}$
i (mA)
i (mA)
E (v vs. RHE)
0.0
0.5
0.75
1.00
0.0
−0.2
−0.4
0.00
−0.05
−0.10
−0.15
−0.20
−0.25

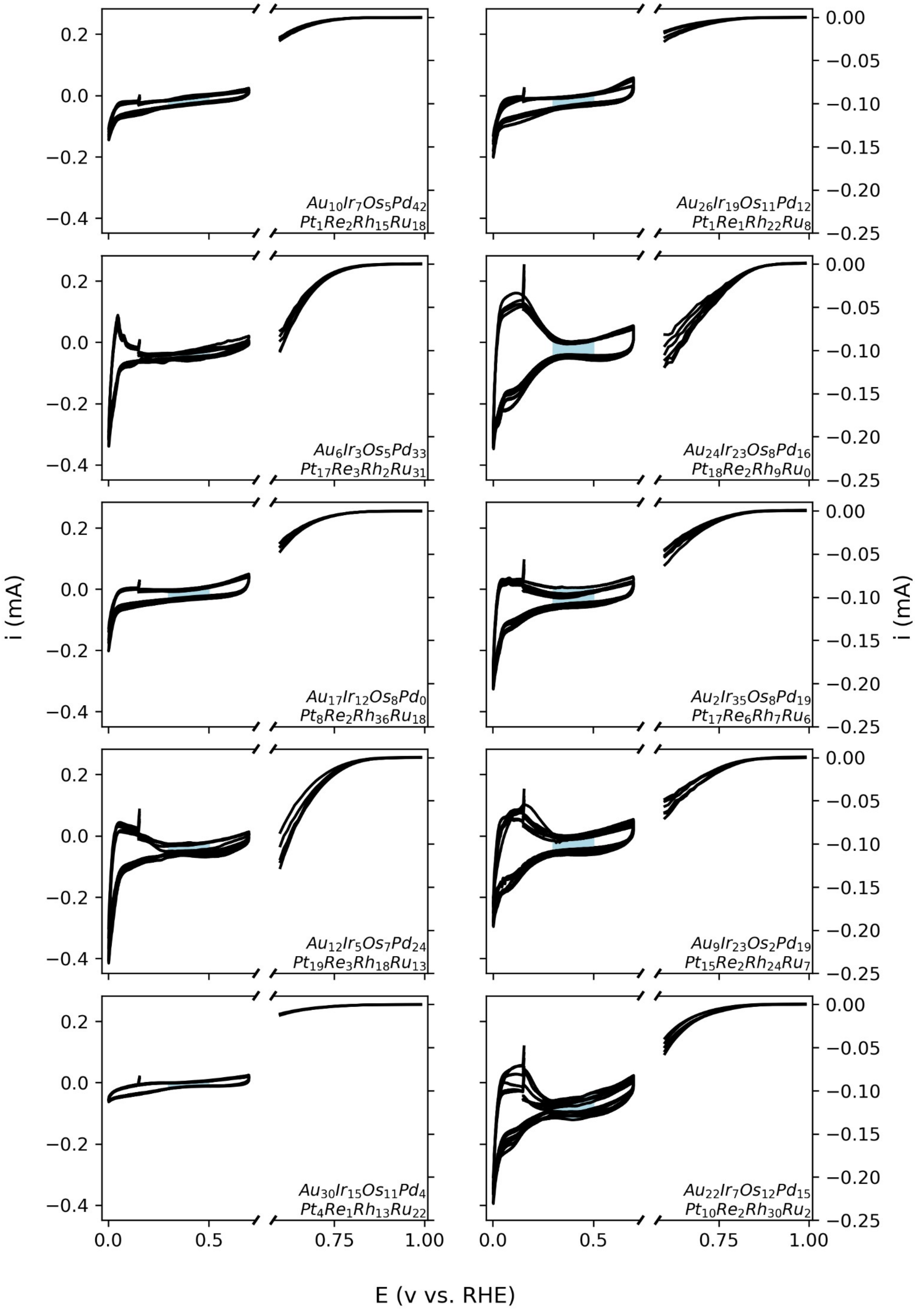

Au10Ir7Os5Pd42 Pt1Re2Rh15Ru18
Au26Ir19Os11Pd12 Pt1Re1Rh22Ru8
Au6Ir3Os5Pd33 Pt17Re3Rh2Ru31
Au24Ir23Os8Pd16 Pt18Re2Rh9Ru0
Au17Ir12Os8Pd0 Pt8Re2Rh36Ru18
Au2Ir35Os8Pd19 Pt17Re6Rh7Ru6
Au12Ir5Os7Pd24 Pt19Re3Rh18Ru13
Au9Ir23Os2Pd19 Pt15Re2Rh24Ru7
Au30Ir15Os11Pd4 Pt4Re1Rh13Ru22
Au22Ir7Os12Pd15 Pt10Re2Rh30Ru2
i (mA)
E (v vs. RHE)

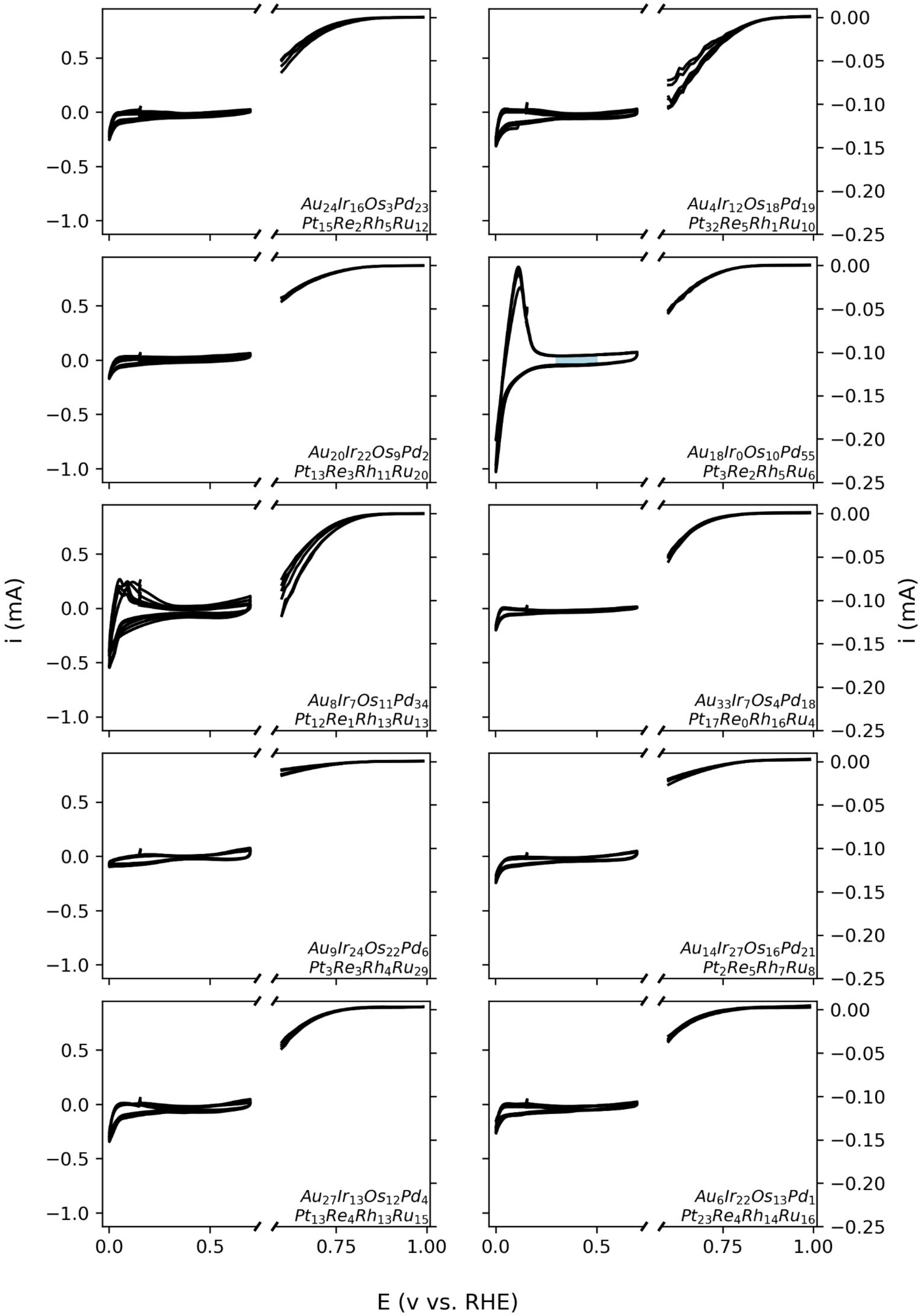

i (mA)
i (mA)
E (v vs. RHE)
$Au_{24}Ir_{16}Os_{3}Pd_{23}$ $Pt_{15}Re_{2}Rh_{5}Ru_{12}$
$Au_{4}Ir_{12}Os_{18}Pd_{19}$ $Pt_{32}Re_{5}Rh_{1}Ru_{10}$
$Au_{20}Ir_{22}Os_{9}Pd_{2}$ $Pt_{13}Re_{3}Rh_{11}Ru_{20}$
$Au_{18}Ir_{0}Os_{10}Pd_{55}$ $Pt_{3}Re_{2}Rh_{5}Ru_{6}$
$Au_{8}Ir_{7}Os_{11}Pd_{34}$ $Pt_{12}Re_{1}Rh_{13}Ru_{13}$
$Au_{33}Ir_{7}Os_{4}Pd_{18}$ $Pt_{17}Re_{0}Rh_{16}Ru_{4}$
$Au_{9}Ir_{24}Os_{22}Pd_{6}$ $Pt_{3}Re_{3}Rh_{4}Ru_{29}$
$Au_{14}Ir_{27}Os_{16}Pd_{21}$ $Pt_{2}Re_{5}Rh_{7}Ru_{8}$
$Au_{27}Ir_{13}Os_{12}Pd_{4}$ $Pt_{13}Re_{4}Rh_{13}Ru_{15}$
$Au_{6}Ir_{22}Os_{13}Pd_{1}$ $Pt_{23}Re_{4}Rh_{14}Ru_{16}$
0.0
0.5
0.75
1.00
0.5
0.0
−0.5
−1.0
0.00
−0.05
−0.10
−0.15
−0.20
−0.25

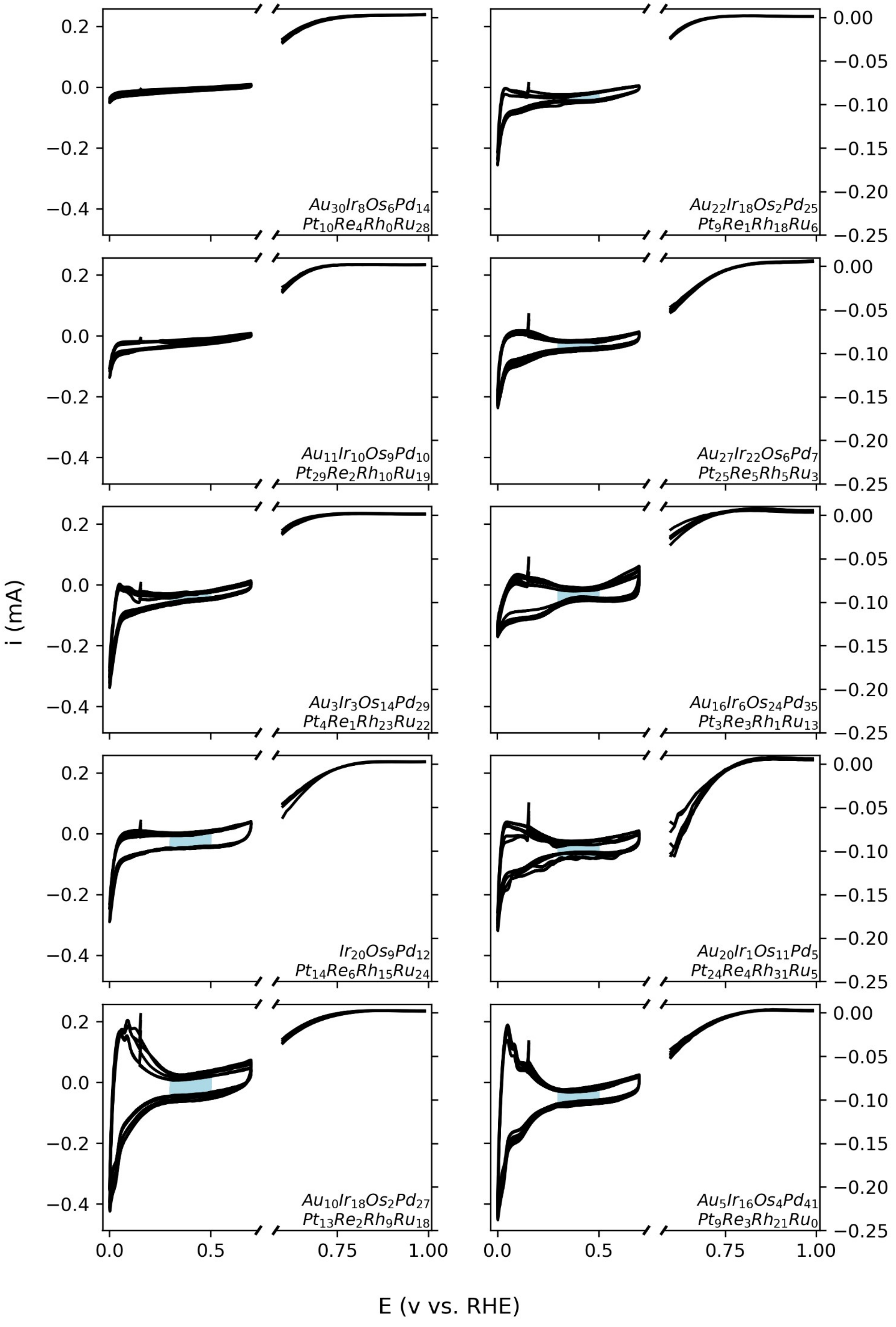

$Au_{30}Ir_{8}Os_{6}Pd_{14}$
$Pt_{10}Re_{4}Rh_{0}Ru_{28}$
$Au_{22}Ir_{18}Os_{2}Pd_{25}$
$Pt_{9}Re_{1}Rh_{18}Ru_{6}$
$Au_{11}Ir_{10}Os_{9}Pd_{10}$
$Pt_{29}Re_{2}Rh_{10}Ru_{19}$
$Au_{27}Ir_{22}Os_{6}Pd_{7}$
$Pt_{25}Re_{5}Rh_{5}Ru_{3}$
$Au_{3}Ir_{3}Os_{14}Pd_{29}$
$Pt_{4}Re_{1}Rh_{23}Ru_{22}$
$Au_{16}Ir_{6}Os_{24}Pd_{35}$
$Pt_{3}Re_{3}Rh_{1}Ru_{13}$
$Ir_{20}Os_{9}Pd_{12}$
$Pt_{14}Re_{6}Rh_{15}Ru_{24}$
$Au_{20}Ir_{1}Os_{11}Pd_{5}$
$Pt_{24}Re_{4}Rh_{31}Ru_{5}$
$Au_{10}Ir_{18}Os_{2}Pd_{27}$
$Pt_{13}Re_{2}Rh_{9}Ru_{18}$
$Au_{5}Ir_{16}Os_{4}Pd_{41}$
$Pt_{9}Re_{3}Rh_{21}Ru_{0}$
i (mA)
i (mA)
E (v vs. RHE)

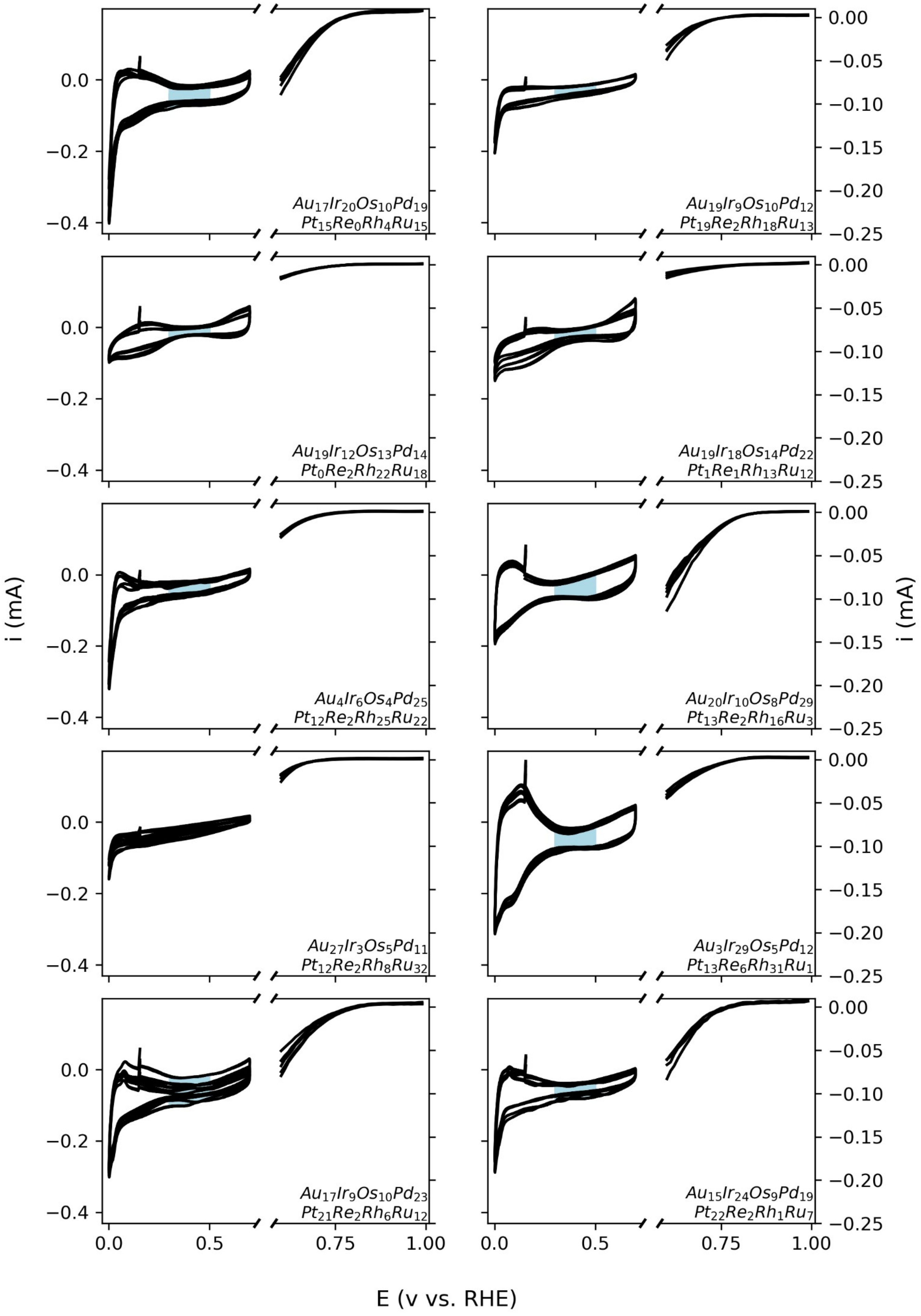

Au17Ir20Os10Pd19 Pt15Re0Rh4Ru15
Au19Ir9Os10Pd12 Pt19Re2Rh18Ru13
Au19Ir12Os13Pd14 Pt0Re2Rh22Ru18
Au19Ir18Os14Pd22 Pt1Re1Rh13Ru12
Au4Ir6Os4Pd25 Pt12Re2Rh25Ru22
Au20Ir10Os8Pd29 Pt13Re2Rh16Ru3
Au27Ir3Os5Pd11 Pt12Re2Rh8Ru32
Au3Ir29Os5Pd12 Pt13Re6Rh31Ru1
Au17Ir9Os10Pd23 Pt21Re2Rh6Ru12
Au15Ir24Os9Pd19 Pt22Re2Rh1Ru7
i (mA)
E (v vs. RHE)

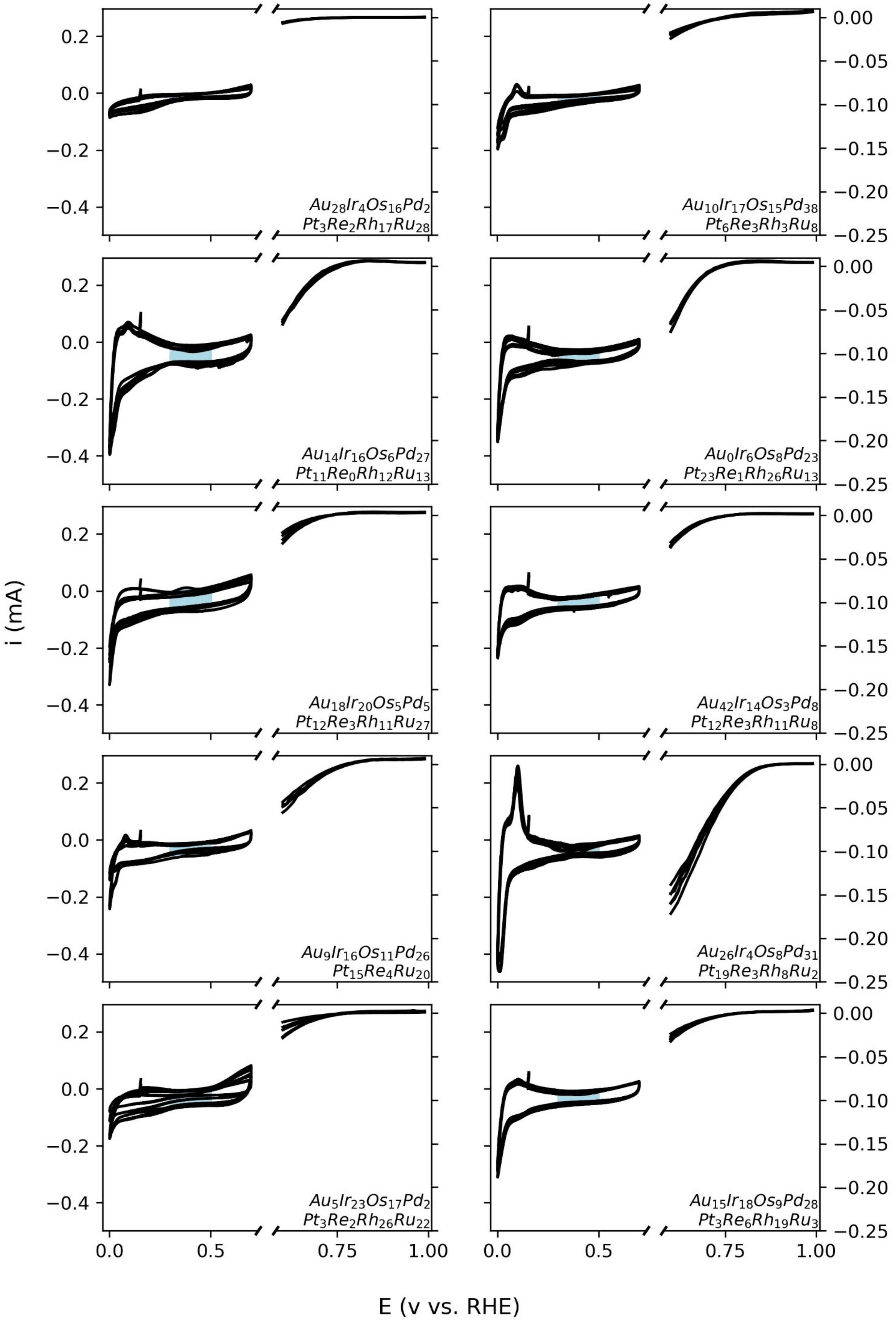

0.2
0.0
−0.2
−0.4
0.00
−0.05
−0.10
−0.15
−0.20
−0.25
$Au_{28}Ir_4Os_{16}Pd_2$
$Pt_3Re_2Rh_{17}Ru_{28}$
$Au_{10}Ir_{17}Os_{15}Pd_{38}$
$Pt_6Re_3Rh_3Ru_8$
$Au_{14}Ir_{16}Os_6Pd_{27}$
$Pt_{11}Re_0Rh_{12}Ru_{13}$
$Au_0Ir_6Os_8Pd_{23}$
$Pt_{23}Re_1Rh_{26}Ru_{13}$
$Au_{18}Ir_{20}Os_5Pd_5$
$Pt_{12}Re_3Rh_{11}Ru_{27}$
$Au_{42}Ir_{14}Os_3Pd_8$
$Pt_{12}Re_3Rh_{11}Ru_8$
$Au_9Ir_{16}Os_{11}Pd_{26}$
$Pt_{15}Re_4Ru_{20}$
$Au_{26}Ir_4Os_8Pd_{31}$
$Pt_{19}Re_3Rh_8Ru_2$
$Au_5Ir_{23}Os_{17}Pd_2$
$Pt_3Re_2Rh_{26}Ru_{22}$
$Au_{15}Ir_{18}Os_9Pd_{28}$
$Pt_3Re_6Rh_{19}Ru_3$
i (mA)
0.0
0.5
0.75
1.00
E (v vs. RHE)

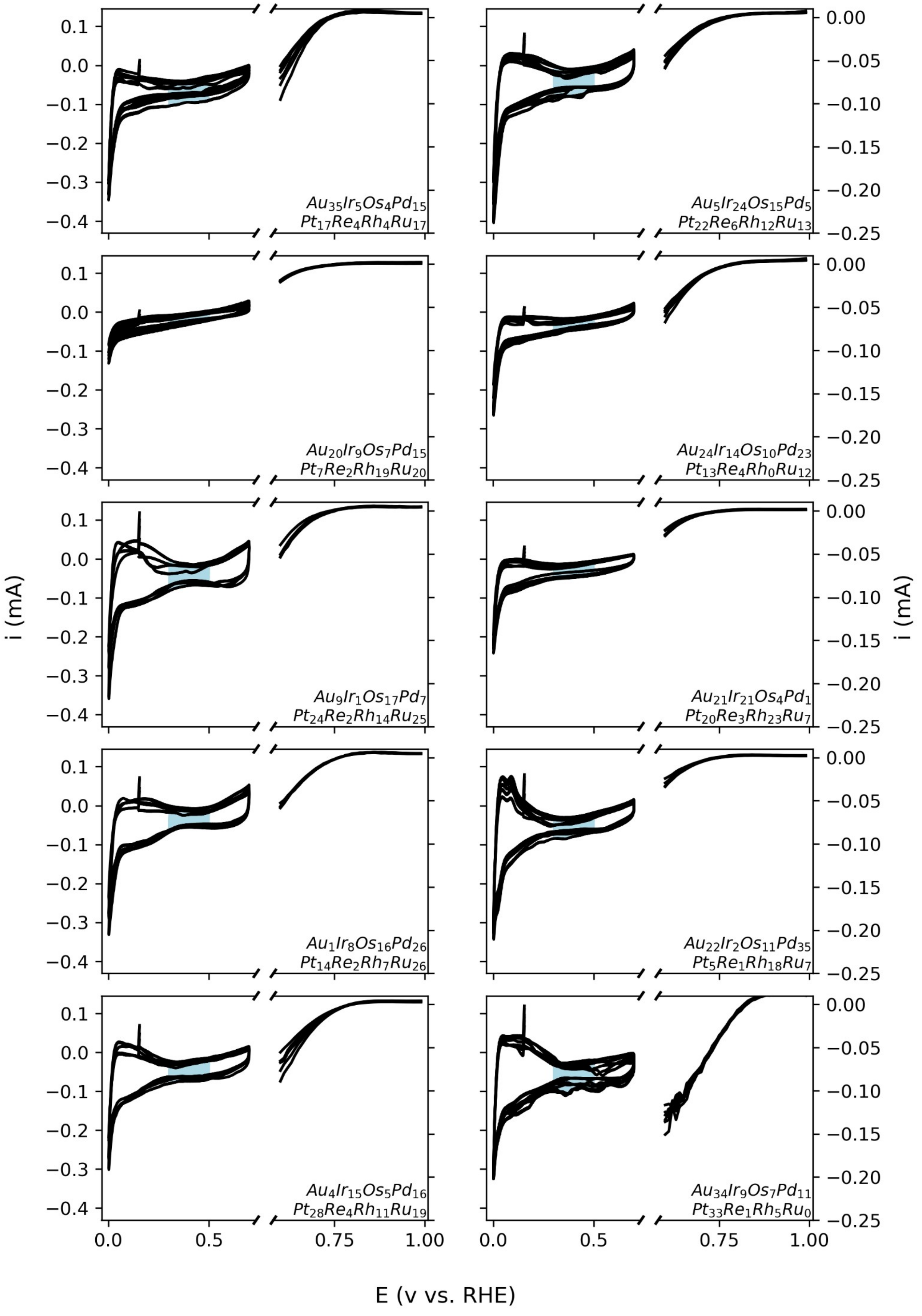
i (mA)
i (mA)
E (v vs. RHE)
$Au_{35}Ir_5Os_4Pd_{15}$
$Pt_{17}Re_4Rh_4Ru_{17}$
$Au_5Ir_{24}Os_{15}Pd_5$
$Pt_{22}Re_6Rh_{12}Ru_{13}$
$Au_{20}Ir_9Os_7Pd_{15}$
$Pt_7Re_2Rh_{19}Ru_{20}$
$Au_{24}Ir_{14}Os_{10}Pd_{23}$
$Pt_{13}Re_4Rh_0Ru_{12}$
$Au_9Ir_1Os_{17}Pd_7$
$Pt_{24}Re_2Rh_{14}Ru_{25}$
$Au_{21}Ir_{21}Os_4Pd_1$
$Pt_{20}Re_3Rh_{23}Ru_7$
$Au_1Ir_8Os_{16}Pd_{26}$
$Pt_{14}Re_2Rh_7Ru_{26}$
$Au_{22}Ir_2Os_{11}Pd_{35}$
$Pt_5Re_1Rh_{18}Ru_7$
$Au_4Ir_{15}Os_5Pd_{16}$
$Pt_{28}Re_4Rh_{11}Ru_{19}$
$Au_{34}Ir_9Os_7Pd_{11}$
$Pt_{33}Re_1Rh_5Ru_0$

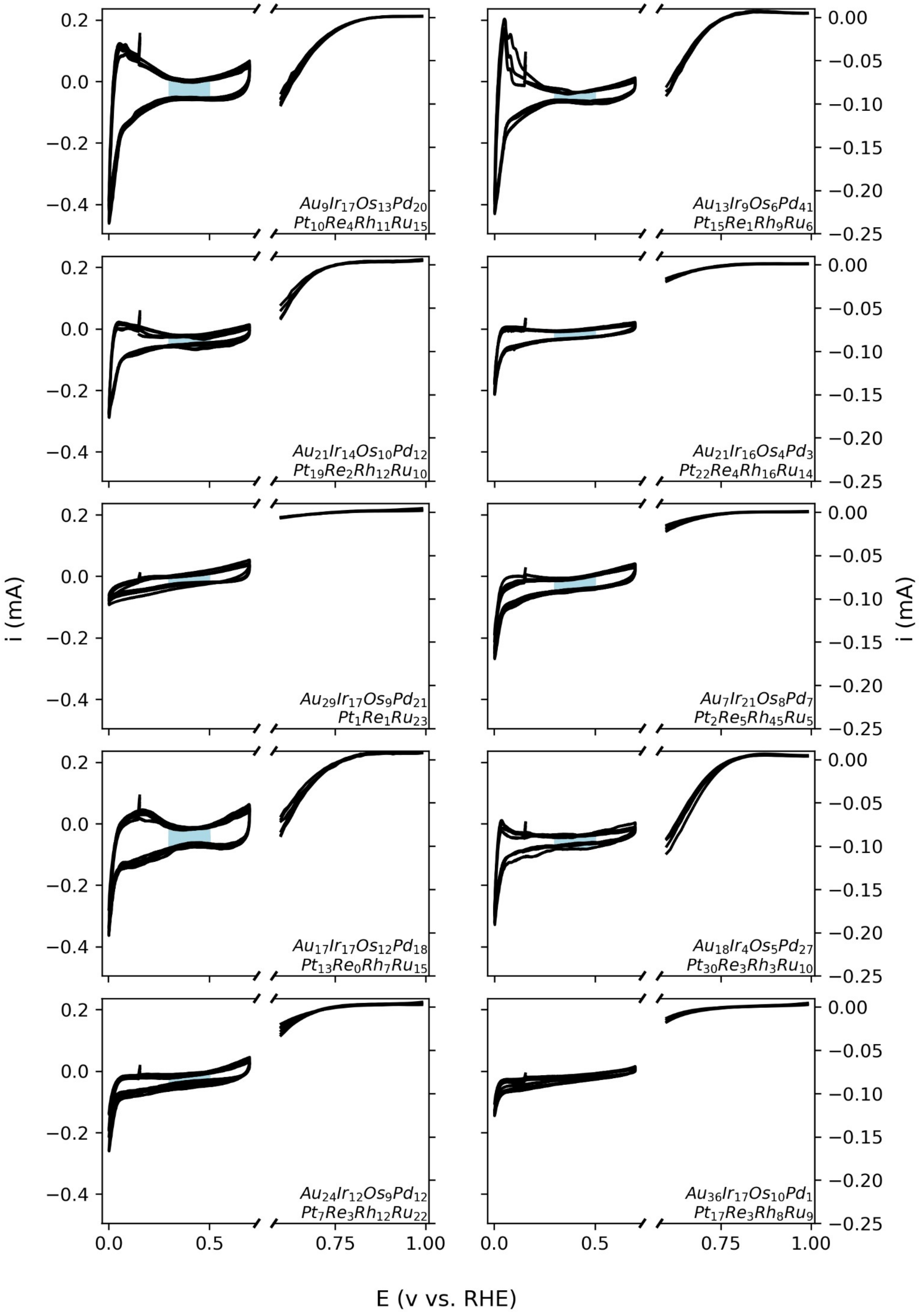

i (mA)
i (mA)
E (v vs. RHE)
$Au_9Ir_{17}Os_{13}Pd_{20}$ $Pt_{10}Re_4Rh_{11}Ru_{15}$
$Au_{13}Ir_9Os_6Pd_{41}$ $Pt_{15}Re_1Rh_9Ru_6$
$Au_{21}Ir_{14}Os_{10}Pd_{12}$ $Pt_{19}Re_2Rh_{12}Ru_{10}$
$Au_{21}Ir_{16}Os_4Pd_3$ $Pt_{22}Re_4Rh_{16}Ru_{14}$
$Au_{29}Ir_{17}Os_9Pd_{21}$ $Pt_1Re_1Ru_{23}$
$Au_7Ir_{21}Os_8Pd_7$ $Pt_2Re_5Rh_{45}Ru_5$
$Au_{17}Ir_{17}Os_{12}Pd_{18}$ $Pt_{13}Re_0Rh_7Ru_{15}$
$Au_{18}Ir_4Os_5Pd_{27}$ $Pt_{30}Re_3Rh_3Ru_{10}$
$Au_{24}Ir_{12}Os_9Pd_{12}$ $Pt_7Re_3Rh_{12}Ru_{22}$
$Au_{36}Ir_{17}Os_{10}Pd_1$ $Pt_{17}Re_3Rh_8Ru_9$

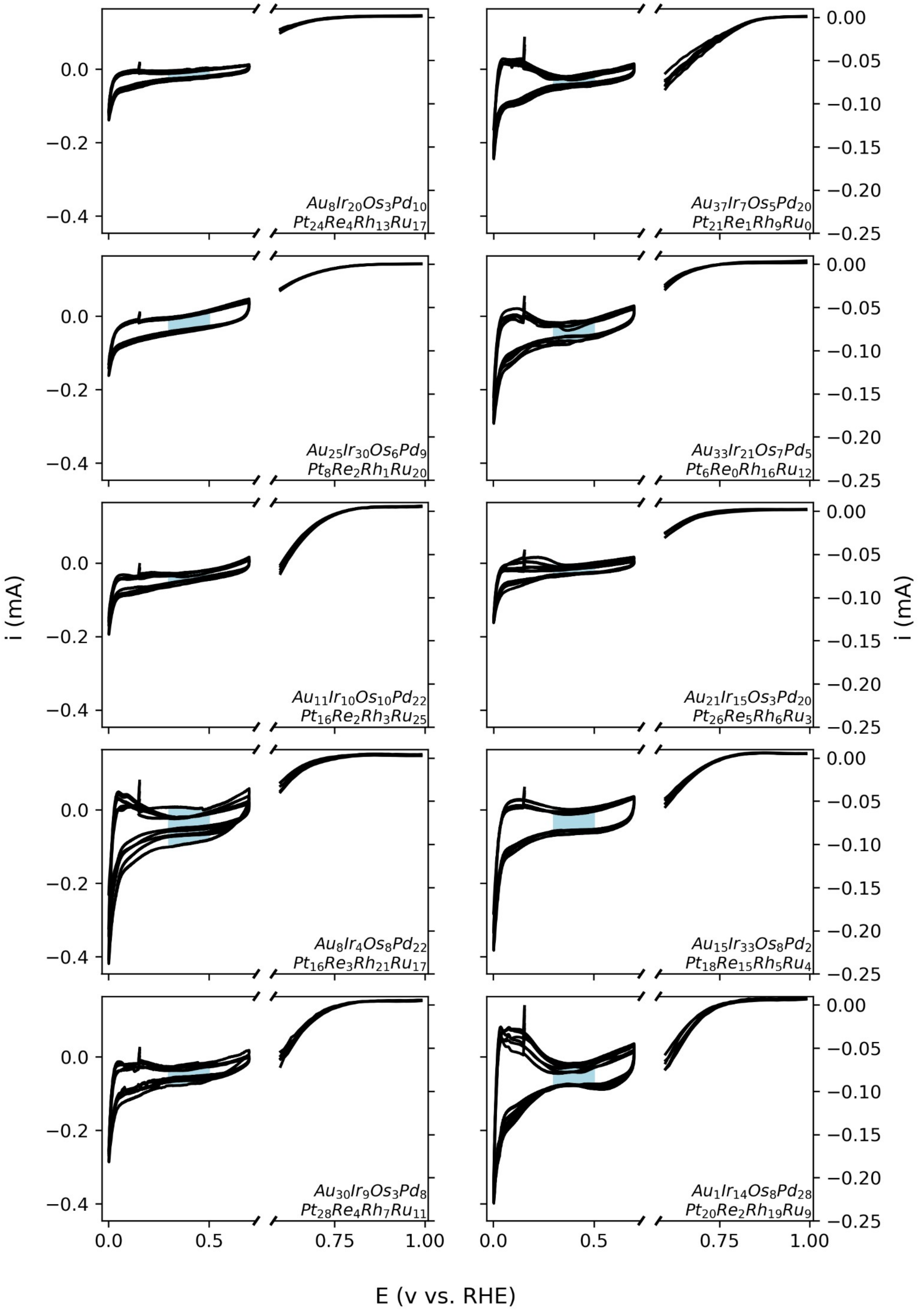

i (mA)
i (mA)
E (v vs. RHE)
Au8Ir20Os3Pd10 Pt24Re4Rh13Ru17
Au37Ir7Os5Pd20 Pt21Re1Rh9Ru0
Au25Ir30Os6Pd9 Pt8Re2Rh1Ru20
Au33Ir21Os7Pd5 Pt6Re0Rh16Ru12
Au11Ir10Os10Pd22 Pt16Re2Rh3Ru25
Au21Ir15Os3Pd20 Pt26Re5Rh6Ru3
Au8Ir4Os8Pd22 Pt16Re3Rh21Ru17
Au15Ir33Os8Pd2 Pt18Re15Rh5Ru4
Au30Ir9Os3Pd8 Pt28Re4Rh7Ru11
Au1Ir14Os8Pd28 Pt20Re2Rh19Ru9

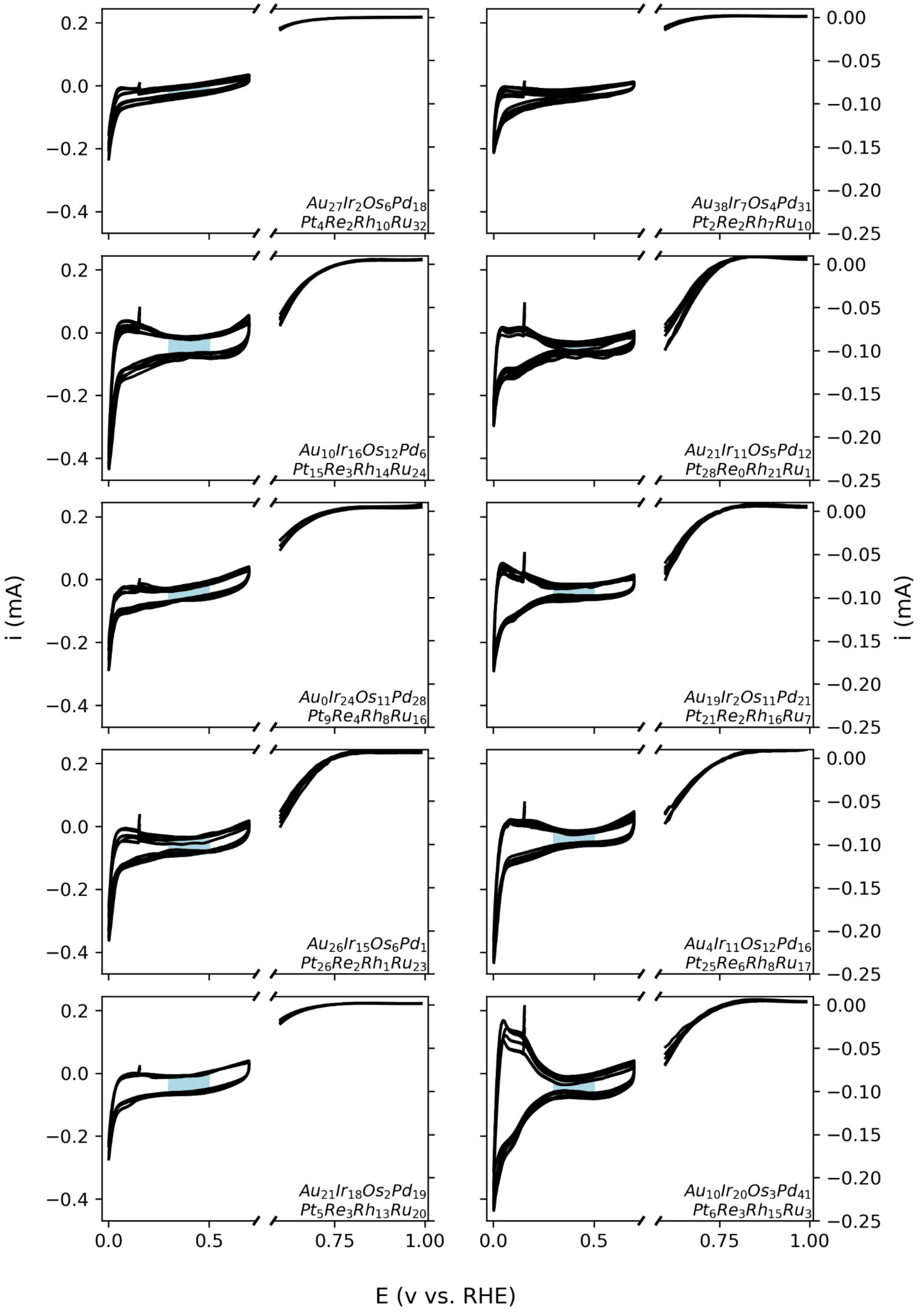

i (mA)
E (v vs. RHE)
$Au_{27}Ir_{2}Os_{6}Pd_{18}$ $Pt_{4}Re_{2}Rh_{10}Ru_{32}$
$Au_{38}Ir_{7}Os_{4}Pd_{31}$ $Pt_{2}Re_{2}Rh_{7}Ru_{10}$
$Au_{10}Ir_{16}Os_{12}Pd_{6}$ $Pt_{15}Re_{3}Rh_{14}Ru_{24}$
$Au_{21}Ir_{11}Os_{5}Pd_{12}$ $Pt_{28}Re_{0}Rh_{21}Ru_{1}$
$Au_{0}Ir_{24}Os_{11}Pd_{28}$ $Pt_{9}Re_{4}Rh_{8}Ru_{16}$
$Au_{19}Ir_{2}Os_{11}Pd_{21}$ $Pt_{21}Re_{2}Rh_{16}Ru_{7}$
$Au_{26}Ir_{15}Os_{6}Pd_{1}$ $Pt_{26}Re_{2}Rh_{1}Ru_{23}$
$Au_{4}Ir_{11}Os_{12}Pd_{16}$ $Pt_{25}Re_{6}Rh_{8}Ru_{17}$
$Au_{21}Ir_{18}Os_{2}Pd_{19}$ $Pt_{5}Re_{3}Rh_{13}Ru_{20}$
$Au_{10}Ir_{20}Os_{3}Pd_{41}$ $Pt_{6}Re_{3}Rh_{15}Ru_{3}$

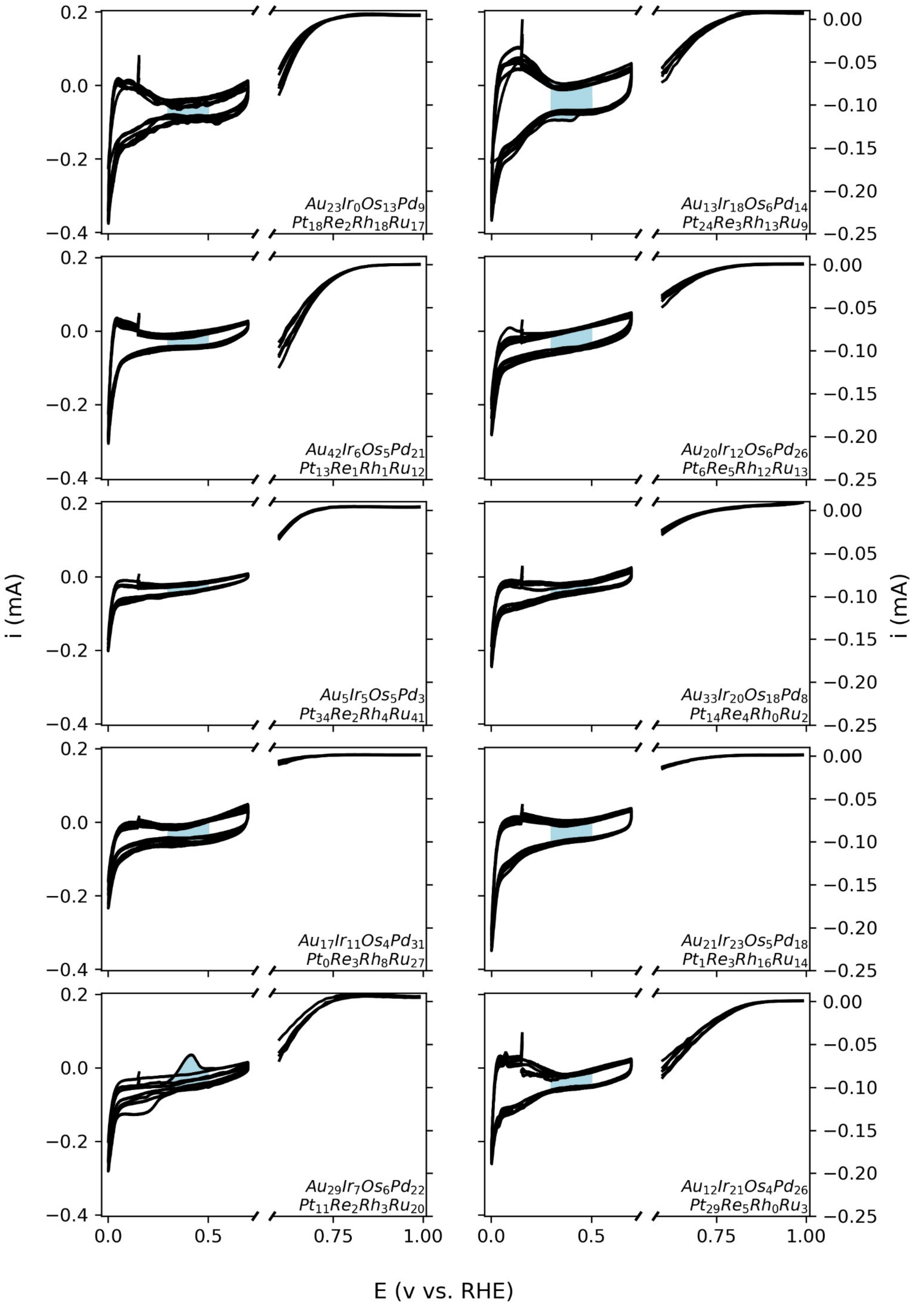

i (mA)
i (mA)
E (v vs. RHE)
Au23Ir0Os13Pd9 Pt18Re2Rh18Ru17
Au13Ir18Os6Pd14 Pt24Re3Rh13Ru9
Au42Ir6Os5Pd21 Pt13Re1Rh1Ru12
Au20Ir12Os6Pd26 Pt6Re5Rh12Ru13
Au5Ir5Os5Pd3 Pt34Re2Rh4Ru41
Au33Ir20Os18Pd8 Pt14Re4Rh0Ru2
Au17Ir11Os4Pd31 Pt0Re3Rh8Ru27
Au21Ir23Os5Pd18 Pt1Re3Rh16Ru14
Au29Ir7Os6Pd22 Pt11Re2Rh3Ru20
Au12Ir21Os4Pd26 Pt29Re5Rh0Ru3

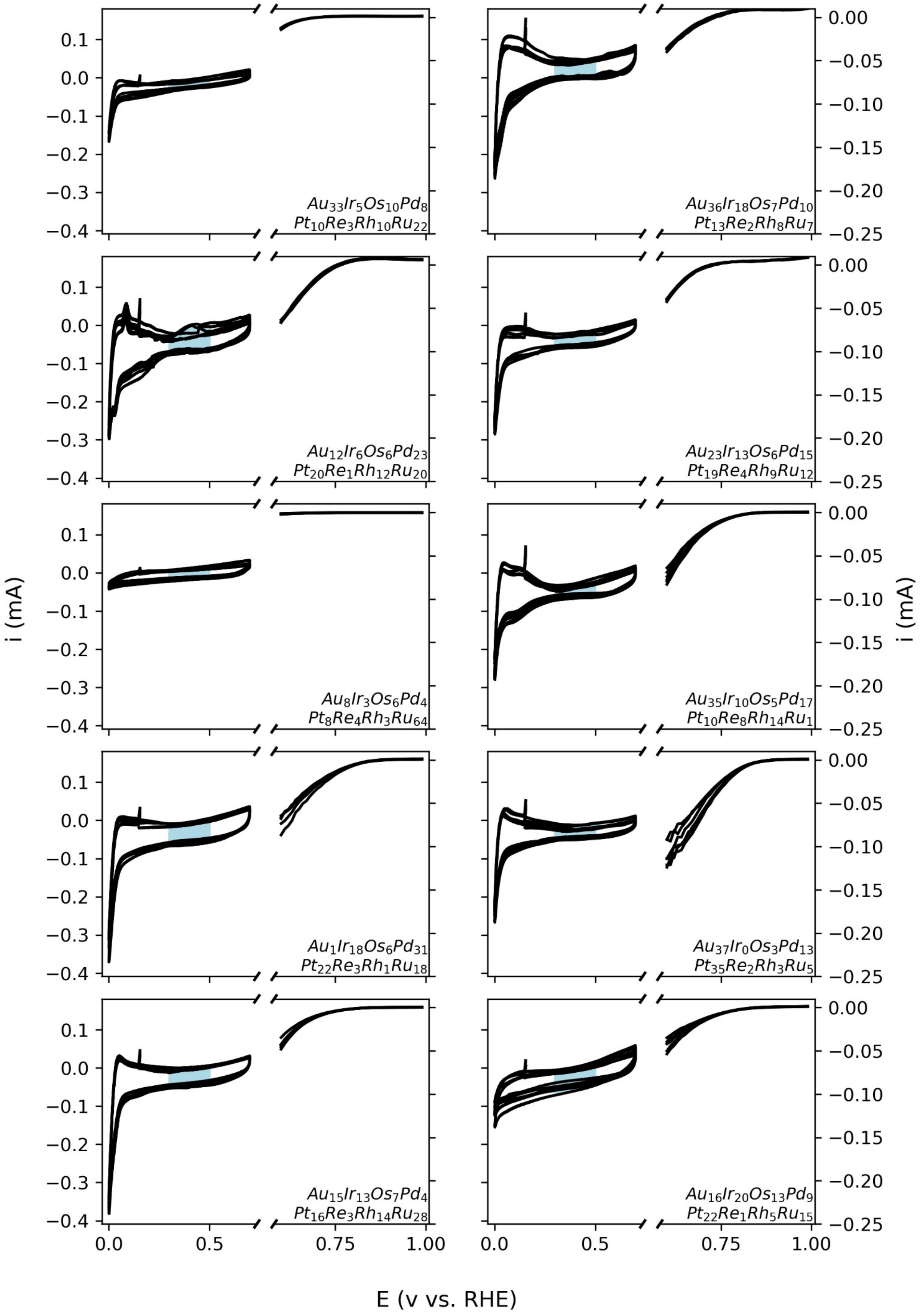

i (mA)
i (mA)
E (v vs. RHE)
$Au_{33}Ir_{5}Os_{10}Pd_{8}$ $Pt_{10}Re_{3}Rh_{10}Ru_{22}$
$Au_{36}Ir_{18}Os_{7}Pd_{10}$ $Pt_{13}Re_{2}Rh_{8}Ru_{7}$
$Au_{12}Ir_{6}Os_{6}Pd_{23}$ $Pt_{20}Re_{1}Rh_{12}Ru_{20}$
$Au_{23}Ir_{13}Os_{6}Pd_{15}$ $Pt_{19}Re_{4}Rh_{9}Ru_{12}$
$Au_{8}Ir_{3}Os_{6}Pd_{4}$ $Pt_{8}Re_{4}Rh_{3}Ru_{64}$
$Au_{35}Ir_{10}Os_{5}Pd_{17}$ $Pt_{10}Re_{8}Rh_{14}Ru_{1}$
$Au_{1}Ir_{18}Os_{6}Pd_{31}$ $Pt_{22}Re_{3}Rh_{1}Ru_{18}$
$Au_{37}Ir_{0}Os_{3}Pd_{13}$ $Pt_{35}Re_{2}Rh_{3}Ru_{5}$
$Au_{15}Ir_{13}Os_{7}Pd_{4}$ $Pt_{16}Re_{3}Rh_{14}Ru_{28}$
$Au_{16}Ir_{20}Os_{13}Pd_{9}$ $Pt_{22}Re_{1}Rh_{5}Ru_{15}$

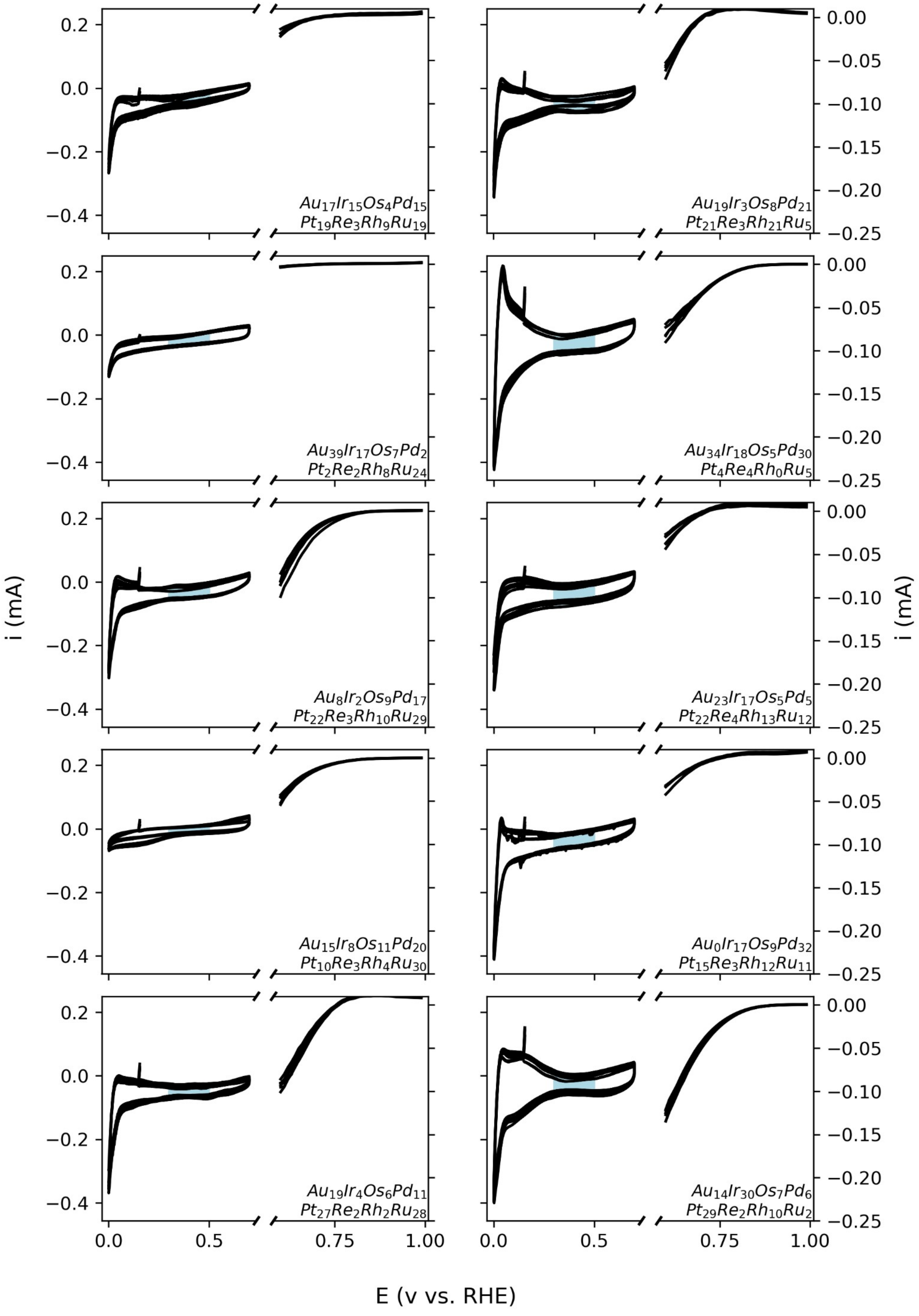

0.2
0.0
−0.2
−0.4
0.00
−0.05
−0.10
−0.15
−0.20
−0.25
i (mA)
i (mA)
$Au_{17}Ir_{15}Os_4Pd_{15}$
$Pt_{19}Re_3Rh_9Ru_{19}$
$Au_{19}Ir_3Os_8Pd_{21}$
$Pt_{21}Re_3Rh_{21}Ru_5$
$Au_{39}Ir_{17}Os_7Pd_2$
$Pt_2Re_2Rh_8Ru_{24}$
$Au_{34}Ir_{18}Os_5Pd_{30}$
$Pt_4Re_4Rh_0Ru_5$
$Au_8Ir_2Os_9Pd_{17}$
$Pt_{22}Re_3Rh_{10}Ru_{29}$
$Au_{23}Ir_{17}Os_5Pd_5$
$Pt_{22}Re_4Rh_{13}Ru_{12}$
$Au_{15}Ir_8Os_{11}Pd_{20}$
$Pt_{10}Re_3Rh_4Ru_{30}$
$Au_0Ir_{17}Os_9Pd_{32}$
$Pt_{15}Re_3Rh_{12}Ru_{11}$
$Au_{19}Ir_4Os_6Pd_{11}$
$Pt_{27}Re_2Rh_2Ru_{28}$
$Au_{14}Ir_{30}Os_7Pd_6$
$Pt_{29}Re_2Rh_{10}Ru_2$
0.0
0.5
0.75
1.00
E (v vs. RHE)

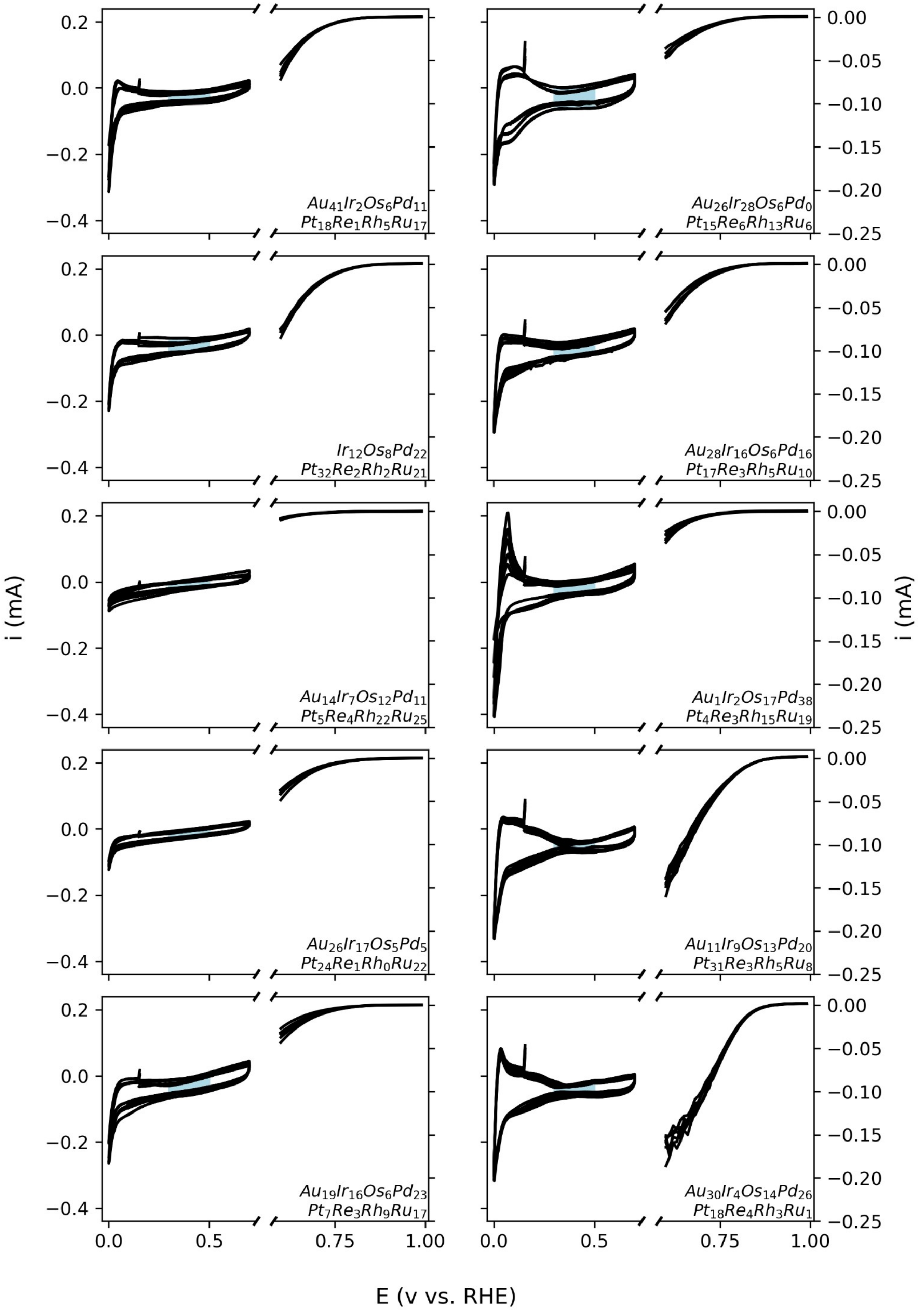

i (mA)
i (mA)
E (v vs. RHE)
Au41Ir2Os6Pd11 Pt18Re1Rh5Ru17
Au26Ir28Os6Pd0 Pt15Re6Rh13Ru6
Ir12Os8Pd22 Pt32Re2Rh2Ru21
Au28Ir16Os6Pd16 Pt17Re3Rh5Ru10
Au14Ir7Os12Pd11 Pt5Re4Rh22Ru25
Au1Ir2Os17Pd38 Pt4Re3Rh15Ru19
Au26Ir17Os5Pd5 Pt24Re1Rh0Ru22
Au11Ir9Os13Pd20 Pt31Re3Rh5Ru8
Au19Ir16Os6Pd23 Pt7Re3Rh9Ru17
Au30Ir4Os14Pd26 Pt18Re4Rh3Ru1

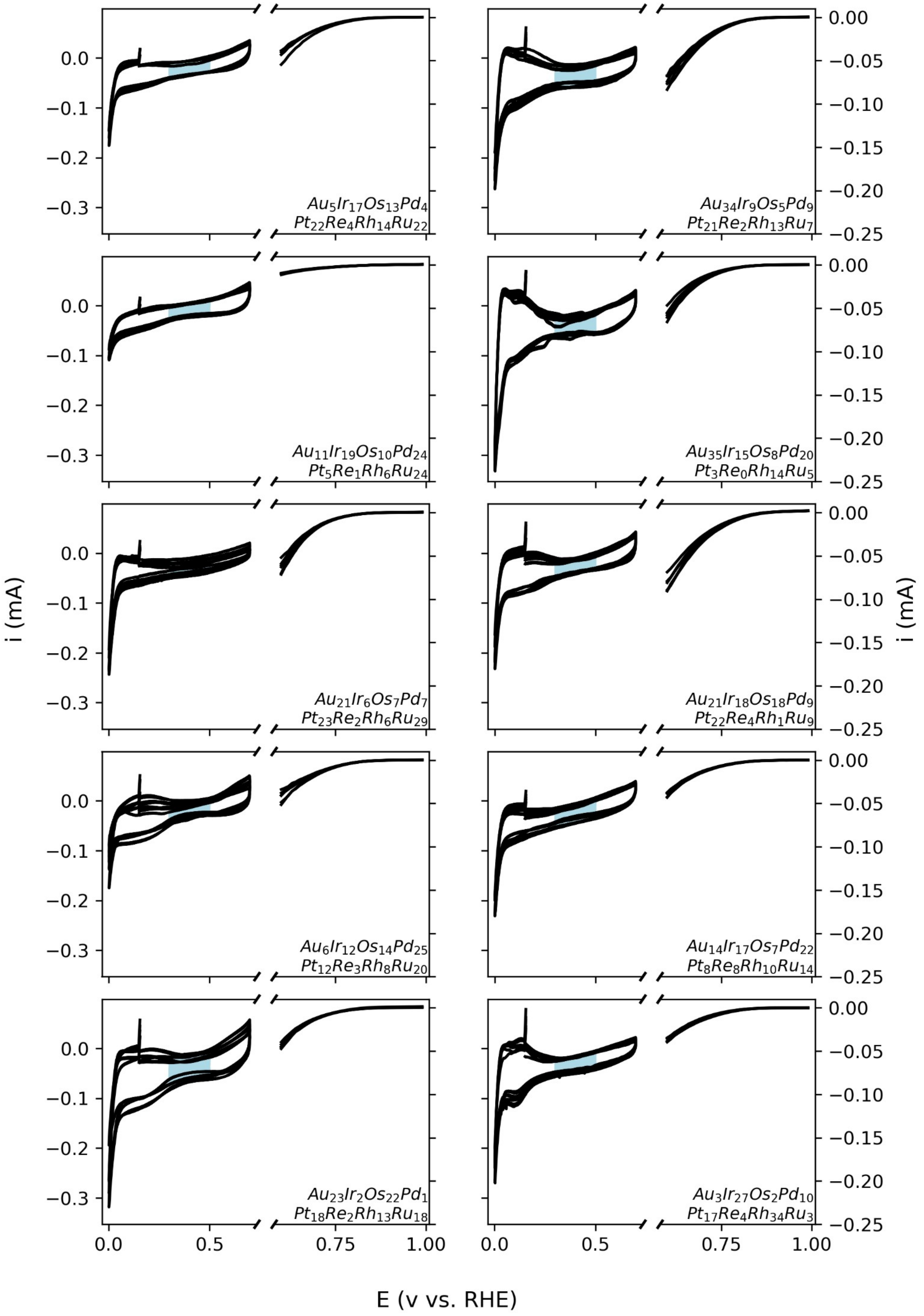
i (mA)
i (mA)
E (v vs. RHE)
0.0
−0.1
−0.2
−0.3
0.00
−0.05
−0.10
−0.15
−0.20
−0.25
0.0
0.5
0.75
1.00
$Au_5Ir_{17}Os_{13}Pd_4$
$Pt_{22}Re_4Rh_{14}Ru_{22}$
$Au_{34}Ir_9Os_5Pd_9$
$Pt_{21}Re_2Rh_{13}Ru_7$
$Au_{11}Ir_{19}Os_{10}Pd_{24}$
$Pt_5Re_1Rh_6Ru_{24}$
$Au_{35}Ir_{15}Os_8Pd_{20}$
$Pt_3Re_0Rh_{14}Ru_5$
$Au_{21}Ir_6Os_7Pd_7$
$Pt_{23}Re_2Rh_6Ru_{29}$
$Au_{21}Ir_{18}Os_{18}Pd_9$
$Pt_{22}Re_4Rh_1Ru_9$
$Au_6Ir_{12}Os_{14}Pd_{25}$
$Pt_{12}Re_3Rh_8Ru_{20}$
$Au_{14}Ir_{17}Os_7Pd_{22}$
$Pt_8Re_8Rh_{10}Ru_{14}$
$Au_{23}Ir_2Os_{22}Pd_1$
$Pt_{18}Re_2Rh_{13}Ru_{18}$
$Au_3Ir_{27}Os_2Pd_{10}$
$Pt_{17}Re_4Rh_{34}Ru_3$

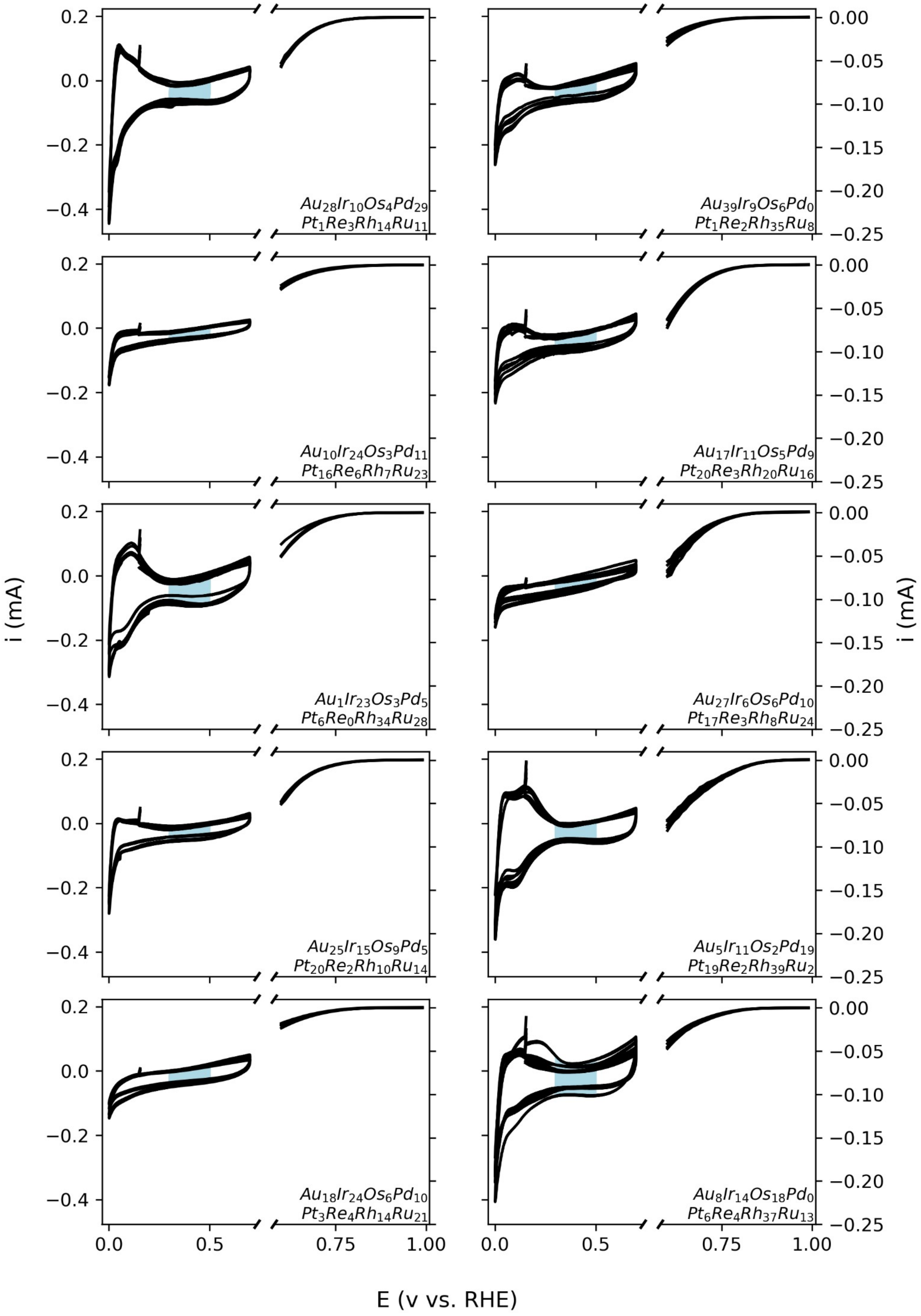

i (mA)
i (mA)
E (v vs. RHE)
Au28Ir10Os4Pd29 Pt1Re3Rh14Ru11
Au39Ir9Os6Pd0 Pt1Re2Rh35Ru8
Au10Ir24Os3Pd11 Pt16Re6Rh7Ru23
Au17Ir11Os5Pd9 Pt20Re3Rh20Ru16
Au1Ir23Os3Pd5 Pt6Re0Rh34Ru28
Au27Ir6Os6Pd10 Pt17Re3Rh8Ru24
Au25Ir15Os9Pd5 Pt20Re2Rh10Ru14
Au5Ir11Os2Pd19 Pt19Re2Rh39Ru2
Au18Ir24Os6Pd10 Pt3Re4Rh14Ru21
Au8Ir14Os18Pd0 Pt6Re4Rh37Ru13

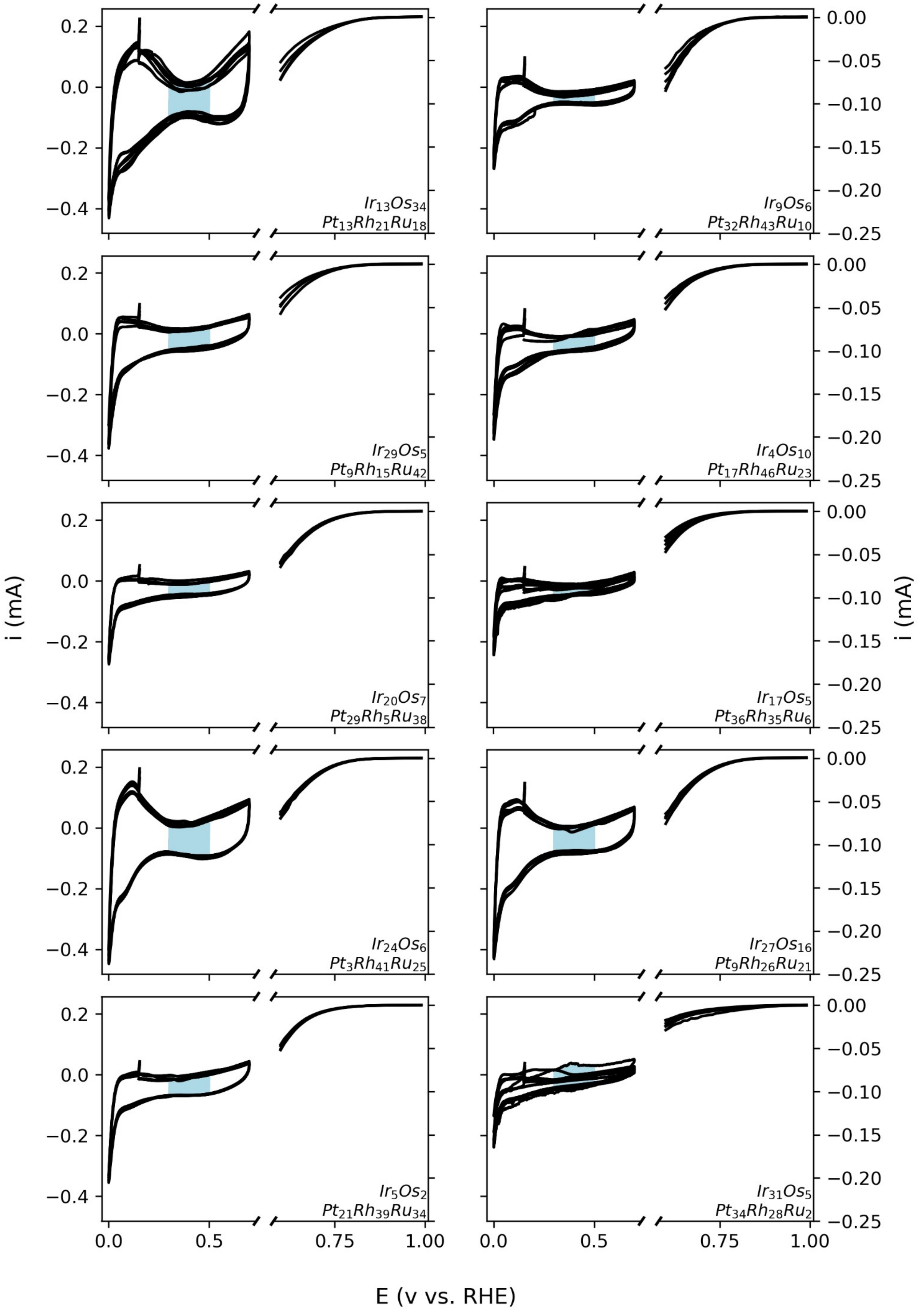

$Ir_{13}Os_{34}$ $Pt_{13}Rh_{21}Ru_{18}$
$Ir_{9}Os_{6}$ $Pt_{32}Rh_{43}Ru_{10}$
$Ir_{29}Os_{5}$ $Pt_{9}Rh_{15}Ru_{42}$
$Ir_{4}Os_{10}$ $Pt_{17}Rh_{46}Ru_{23}$
$Ir_{20}Os_{7}$ $Pt_{29}Rh_{5}Ru_{38}$
$Ir_{17}Os_{5}$ $Pt_{36}Rh_{35}Ru_{6}$
$Ir_{24}Os_{6}$ $Pt_{3}Rh_{41}Ru_{25}$
$Ir_{27}Os_{16}$ $Pt_{9}Rh_{26}Ru_{21}$
$Ir_{5}Os_{2}$ $Pt_{21}Rh_{39}Ru_{34}$
$Ir_{31}Os_{5}$ $Pt_{34}Rh_{28}Ru_{2}$
i (mA)
i (mA)
E (v vs. RHE)

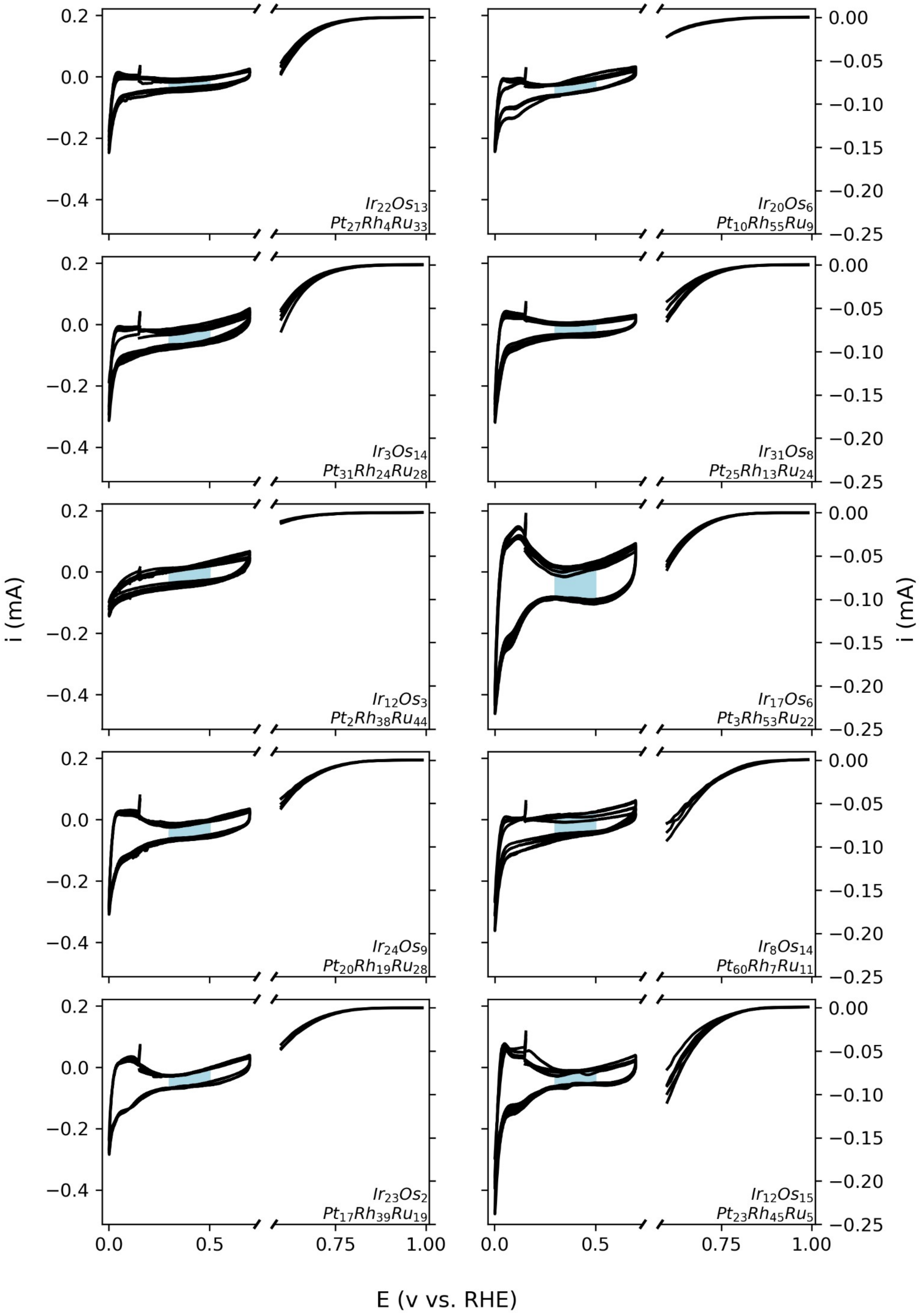

Ir22Os13
Pt27Rh4Ru33
Ir20Os6
Pt10Rh55Ru9
Ir3Os14
Pt31Rh24Ru28
Ir31Os8
Pt25Rh13Ru24
Ir12Os3
Pt2Rh38Ru44
Ir17Os6
Pt3Rh53Ru22
Ir24Os9
Pt20Rh19Ru28
Ir8Os14
Pt60Rh7Ru11
Ir23Os2
Pt17Rh39Ru19
Ir12Os15
Pt23Rh45Ru5
i (mA)
i (mA)
E (v vs. RHE)

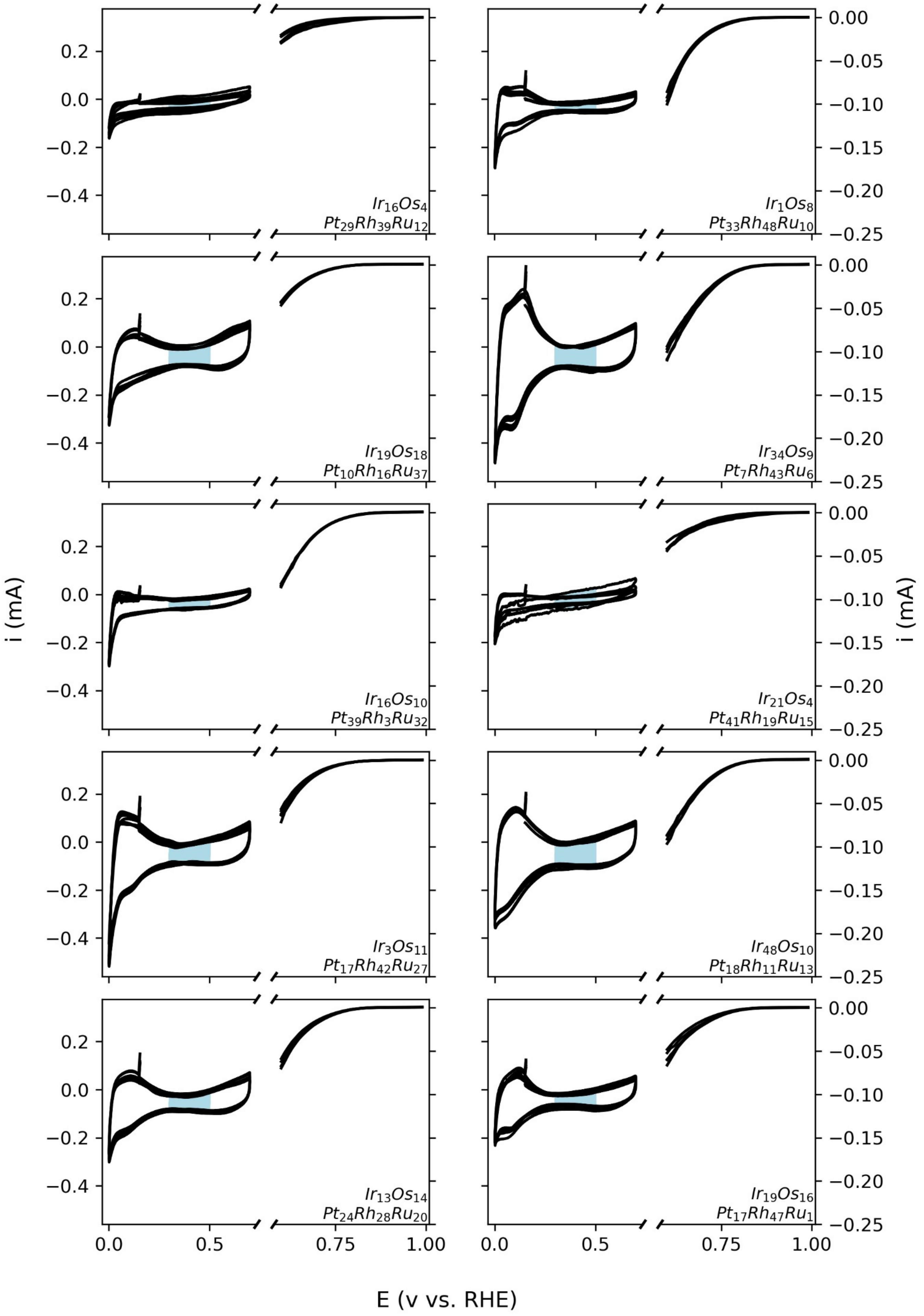

$Ir_{16}Os_4$ $Pt_{29}Rh_{39}Ru_{12}$
Ir_1Os_8 $Pt_{33}Rh_{48}Ru_{10}$
$Ir_{19}Os_{18}$ $Pt_{10}Rh_{16}Ru_{37}$
$Ir_{34}Os_9$ $Pt_7Rh_{43}Ru_6$
$Ir_{16}Os_{10}$ $Pt_{39}Rh_3Ru_{32}$
$Ir_{21}Os_4$ $Pt_{41}Rh_{19}Ru_{15}$
Ir_3Os_{11} $Pt_{17}Rh_{42}Ru_{27}$
$Ir_{48}Os_{10}$ $Pt_{18}Rh_{11}Ru_{13}$
$Ir_{13}Os_{14}$ $Pt_{24}Rh_{28}Ru_{20}$
$Ir_{19}Os_{16}$ $Pt_{17}Rh_{47}Ru_1$
i (mA)
i (mA)
E (v vs. RHE)

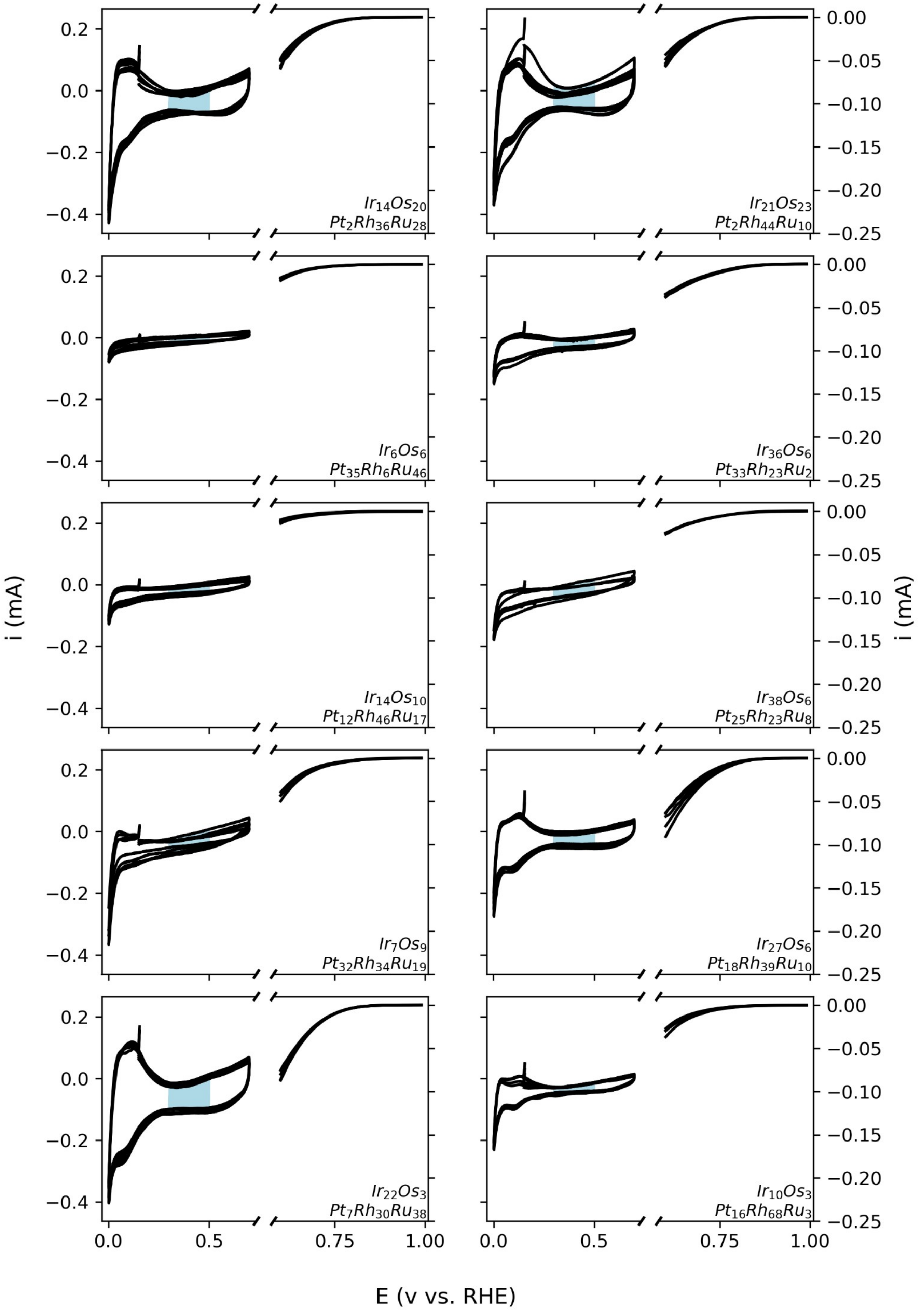
$Ir_{14}Os_{20}$ $Pt_2Rh_{36}Ru_{28}$
$Ir_{21}Os_{23}$ $Pt_2Rh_{44}Ru_{10}$
Ir_6Os_6 $Pt_{35}Rh_6Ru_{46}$
$Ir_{36}Os_6$ $Pt_{33}Rh_{23}Ru_2$
$Ir_{14}Os_{10}$ $Pt_{12}Rh_{46}Ru_{17}$
$Ir_{38}Os_6$ $Pt_{25}Rh_{23}Ru_8$
Ir_7Os_9 $Pt_{32}Rh_{34}Ru_{19}$
$Ir_{27}Os_6$ $Pt_{18}Rh_{39}Ru_{10}$
$Ir_{22}Os_3$ $Pt_7Rh_{30}Ru_{38}$
$Ir_{10}Os_3$ $Pt_{16}Rh_{68}Ru_3$
i (mA)
i (mA)
E (v vs. RHE)
0.2
0.0
−0.2
−0.4
0.00
−0.05
−0.10
−0.15
−0.20
−0.25
0.0
0.5
0.75
1.00

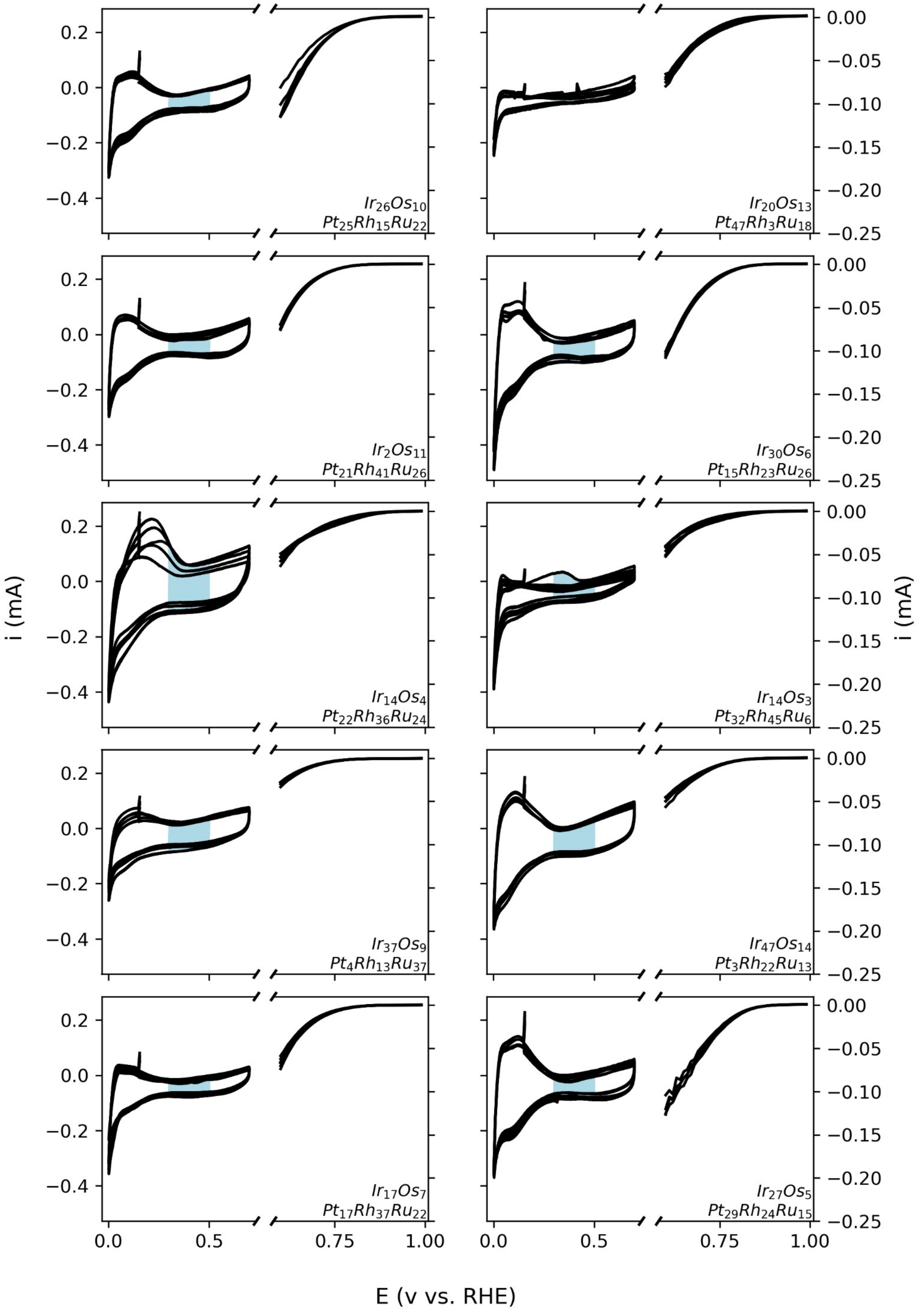
i (mA)
i (mA)
E (v vs. RHE)
$Ir_{26}Os_{10}$ $Pt_{25}Rh_{15}Ru_{22}$
$Ir_{20}Os_{13}$ $Pt_{47}Rh_{3}Ru_{18}$
$Ir_{2}Os_{11}$ $Pt_{21}Rh_{41}Ru_{26}$
$Ir_{30}Os_{6}$ $Pt_{15}Rh_{23}Ru_{26}$
$Ir_{14}Os_{4}$ $Pt_{22}Rh_{36}Ru_{24}$
$Ir_{14}Os_{3}$ $Pt_{32}Rh_{45}Ru_{6}$
$Ir_{37}Os_{9}$ $Pt_{4}Rh_{13}Ru_{37}$
$Ir_{47}Os_{14}$ $Pt_{3}Rh_{22}Ru_{13}$
$Ir_{17}Os_{7}$ $Pt_{17}Rh_{37}Ru_{22}$
$Ir_{27}Os_{5}$ $Pt_{29}Rh_{24}Ru_{15}$

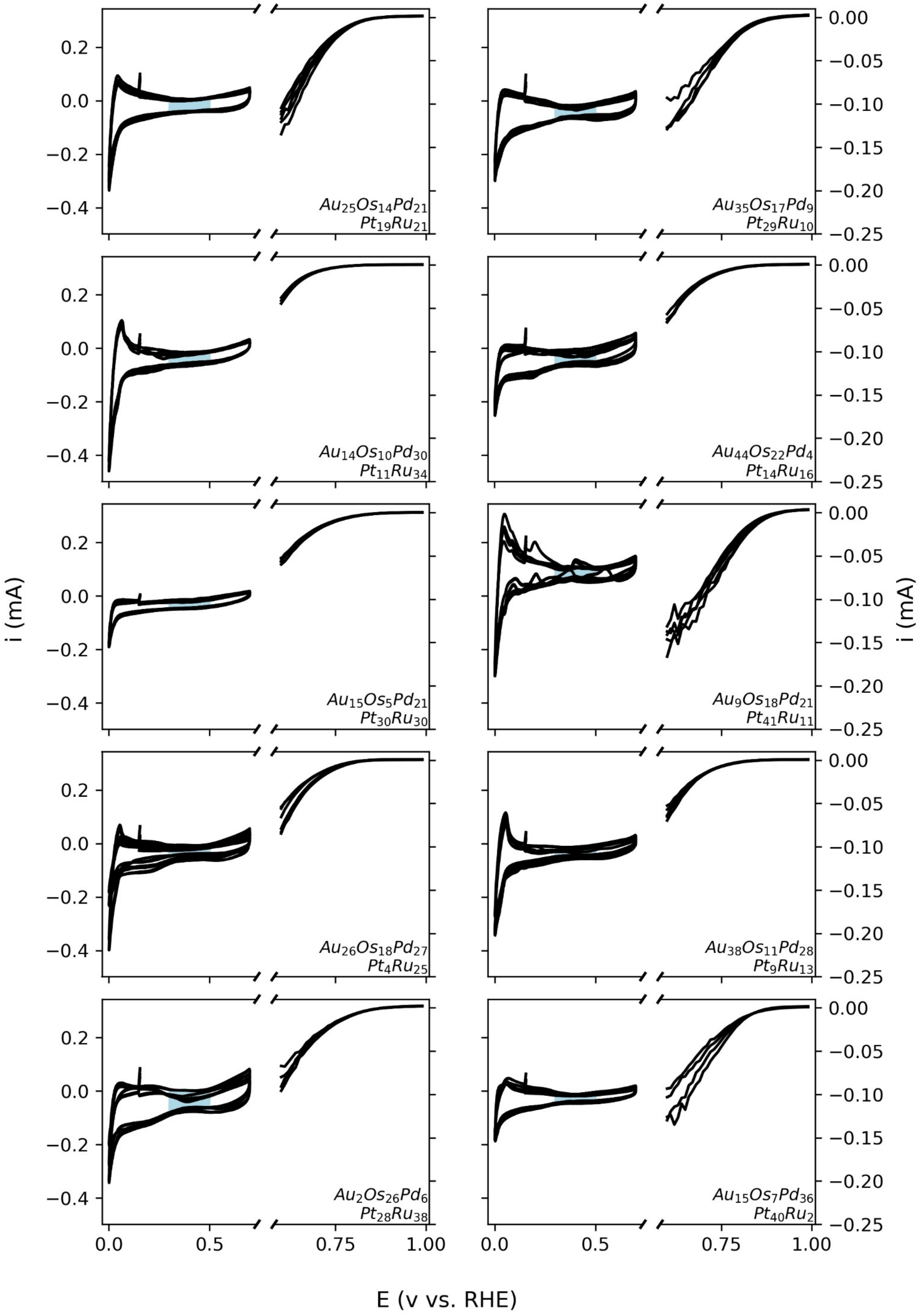

$Au_{25}Os_{14}Pd_{21}$
$Pt_{19}Ru_{21}$
$Au_{35}Os_{17}Pd_{9}$
$Pt_{29}Ru_{10}$
$Au_{14}Os_{10}Pd_{30}$
$Pt_{11}Ru_{34}$
$Au_{44}Os_{22}Pd_{4}$
$Pt_{14}Ru_{16}$
$Au_{15}Os_{5}Pd_{21}$
$Pt_{30}Ru_{30}$
$Au_{9}Os_{18}Pd_{21}$
$Pt_{41}Ru_{11}$
$Au_{26}Os_{18}Pd_{27}$
$Pt_{4}Ru_{25}$
$Au_{38}Os_{11}Pd_{28}$
$Pt_{9}Ru_{13}$
$Au_{2}Os_{26}Pd_{6}$
$Pt_{28}Ru_{38}$
$Au_{15}Os_{7}Pd_{36}$
$Pt_{40}Ru_{2}$
i (mA)
i (mA)
E (v vs. RHE)

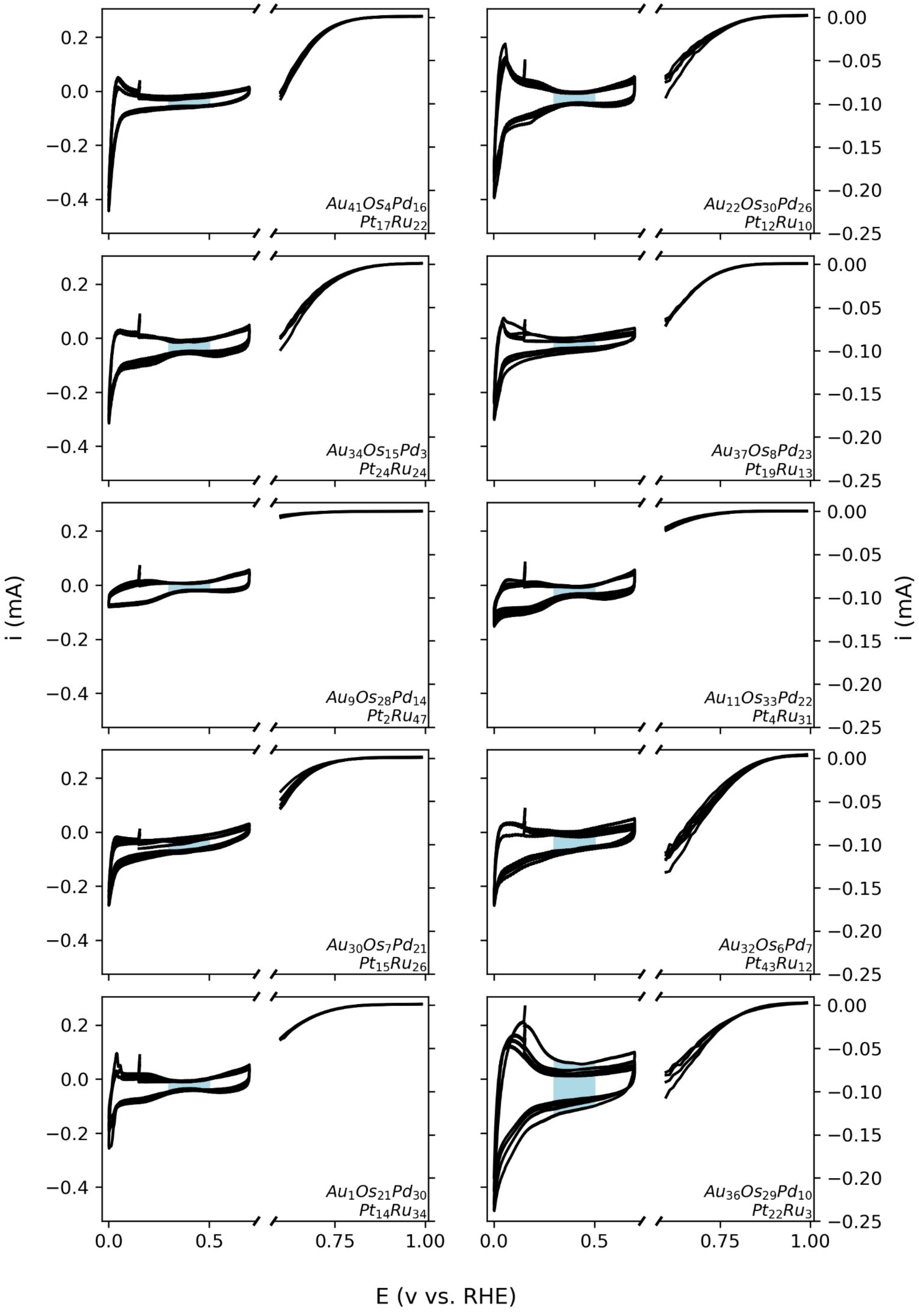

0.2
0.0
−0.2
−0.4
0.00
−0.05
−0.10
−0.15
−0.20
−0.25
i (mA)
i (mA)
$Au_{41}Os_4Pd_{16}Pt_{17}Ru_{22}$
$Au_{22}Os_{30}Pd_{26}Pt_{12}Ru_{10}$
$Au_{34}Os_{15}Pd_3Pt_{24}Ru_{24}$
$Au_{37}Os_8Pd_{23}Pt_{19}Ru_{13}$
$Au_9Os_{28}Pd_{14}Pt_2Ru_{47}$
$Au_{11}Os_{33}Pd_{22}Pt_4Ru_{31}$
$Au_{30}Os_7Pd_{21}Pt_{15}Ru_{26}$
$Au_{32}Os_6Pd_7Pt_{43}Ru_{12}$
$Au_1Os_{21}Pd_{30}Pt_{14}Ru_{34}$
$Au_{36}Os_{29}Pd_{10}Pt_{22}Ru_3$
0.0
0.5
0.75
1.00
E (v vs. RHE)

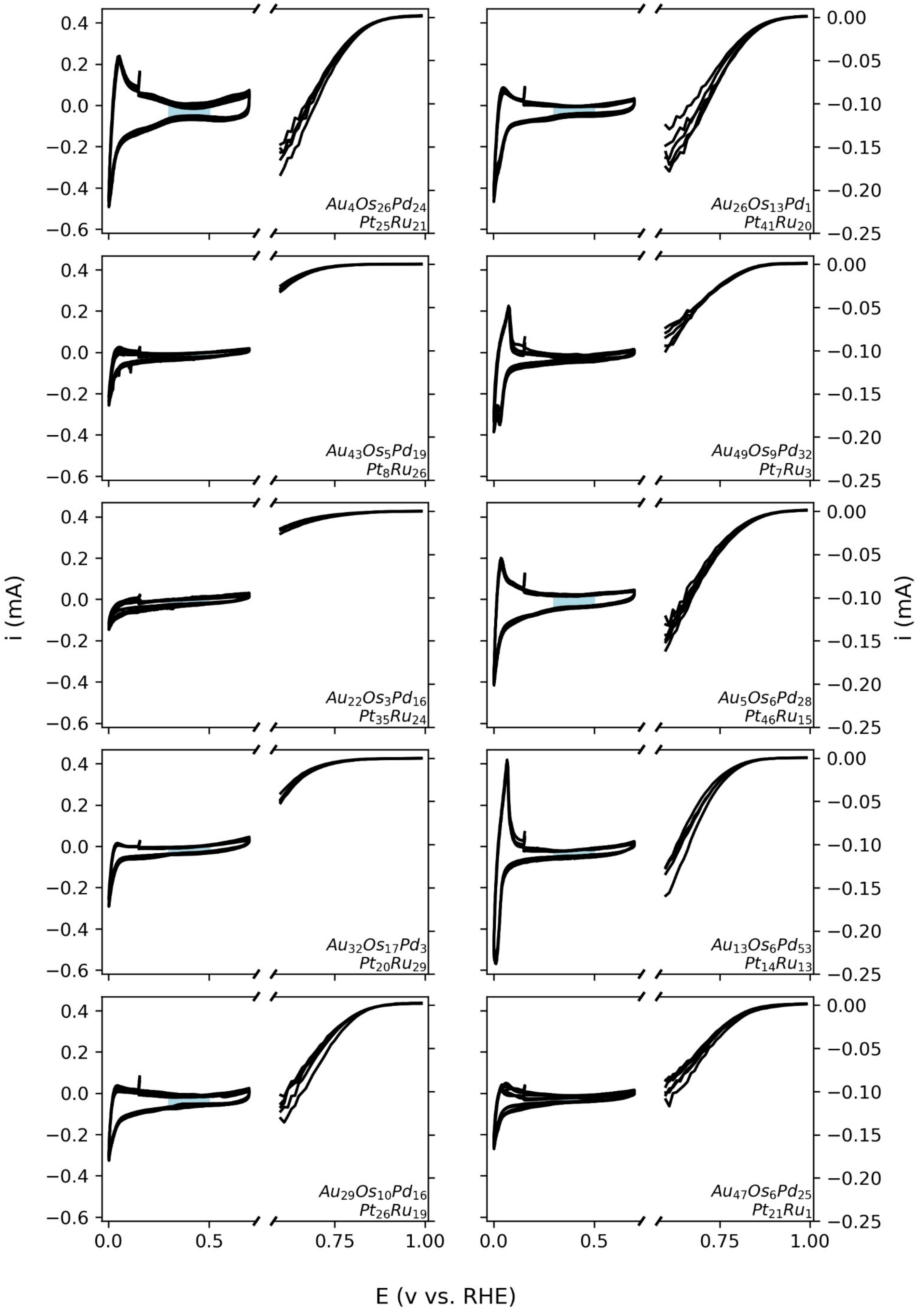
i (mA)
E (v vs. RHE)
$Au_4Os_{26}Pd_{24}$ $Pt_{25}Ru_{21}$
$Au_{26}Os_{13}Pd_1$ $Pt_{41}Ru_{20}$
$Au_{43}Os_5Pd_{19}$ Pt_8Ru_{26}
$Au_{49}Os_9Pd_{32}$ Pt_7Ru_3
$Au_{22}Os_3Pd_{16}$ $Pt_{35}Ru_{24}$
$Au_5Os_6Pd_{28}$ $Pt_{46}Ru_{15}$
$Au_{32}Os_{17}Pd_3$ $Pt_{20}Ru_{29}$
$Au_{13}Os_6Pd_{53}$ $Pt_{14}Ru_{13}$
$Au_{29}Os_{10}Pd_{16}$ $Pt_{26}Ru_{19}$
$Au_{47}Os_6Pd_{25}$ $Pt_{21}Ru_1$

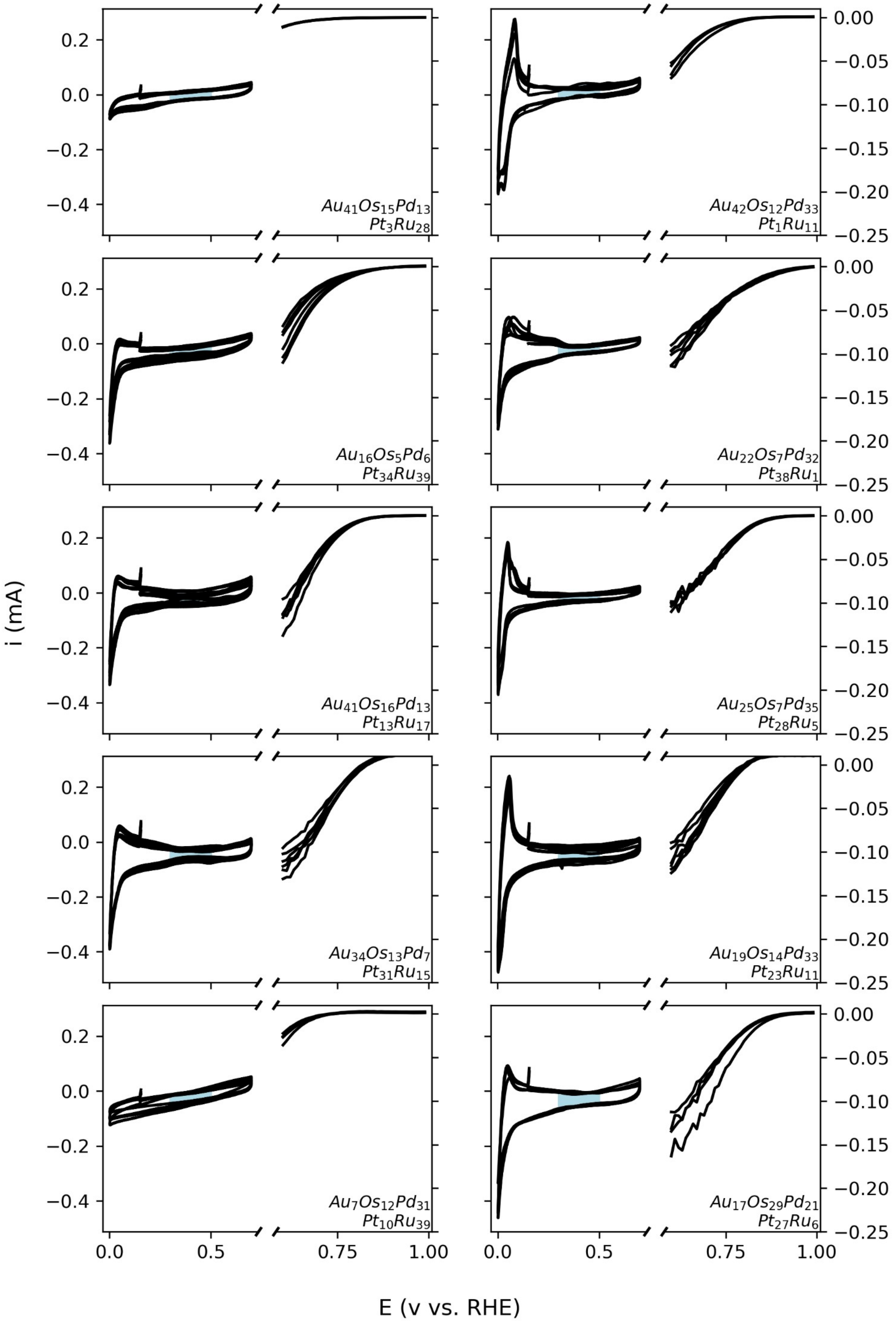

i (mA)
i (mA)
E (v vs. RHE)
$Au_{41}Os_{15}Pd_{13}$ $Pt_{3}Ru_{28}$
$Au_{42}Os_{12}Pd_{33}$ $Pt_{1}Ru_{11}$
$Au_{16}Os_{5}Pd_{6}$ $Pt_{34}Ru_{39}$
$Au_{22}Os_{7}Pd_{32}$ $Pt_{38}Ru_{1}$
$Au_{41}Os_{16}Pd_{13}$ $Pt_{13}Ru_{17}$
$Au_{25}Os_{7}Pd_{35}$ $Pt_{28}Ru_{5}$
$Au_{34}Os_{13}Pd_{7}$ $Pt_{31}Ru_{15}$
$Au_{19}Os_{14}Pd_{33}$ $Pt_{23}Ru_{11}$
$Au_{7}Os_{12}Pd_{31}$ $Pt_{10}Ru_{39}$
$Au_{17}Os_{29}Pd_{21}$ $Pt_{27}Ru_{6}$
0.2
0.0
−0.2
−0.4
0.00
−0.05
−0.10
−0.15
−0.20
−0.25
0.0
0.5
0.75
1.00

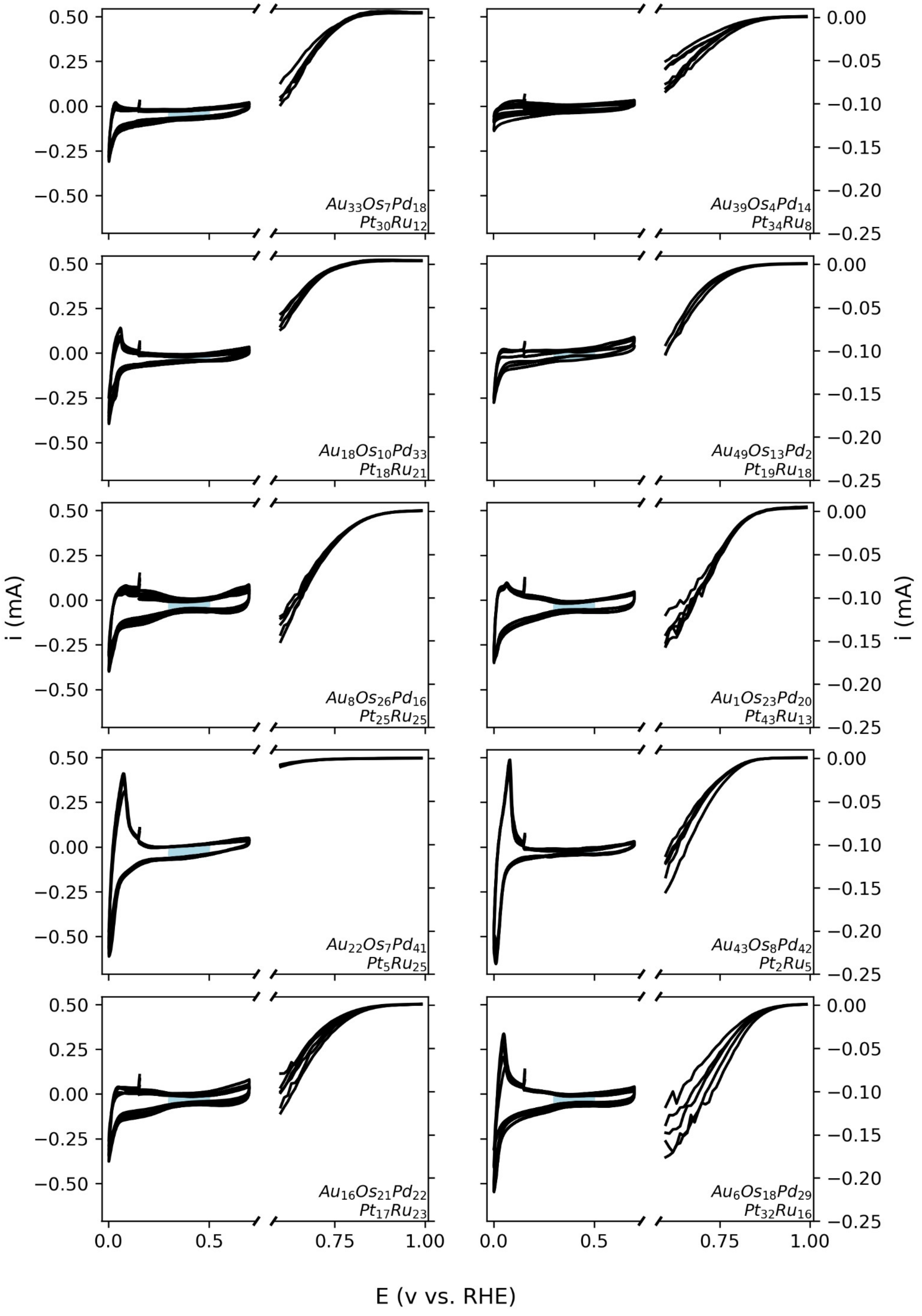

i (mA)
E (v vs. RHE)
$Au_{33}Os_7Pd_{18}Pt_{30}Ru_{12}$
$Au_{39}Os_4Pd_{14}Pt_{34}Ru_8$
$Au_{18}Os_{10}Pd_{33}Pt_{18}Ru_{21}$
$Au_{49}Os_{13}Pd_2Pt_{19}Ru_{18}$
$Au_8Os_{26}Pd_{16}Pt_{25}Ru_{25}$
$Au_1Os_{23}Pd_{20}Pt_{43}Ru_{13}$
$Au_{22}Os_7Pd_{41}Pt_5Ru_{25}$
$Au_{43}Os_8Pd_{42}Pt_2Ru_5$
$Au_{16}Os_{21}Pd_{22}Pt_{17}Ru_{23}$
$Au_6Os_{18}Pd_{29}Pt_{32}Ru_{16}$

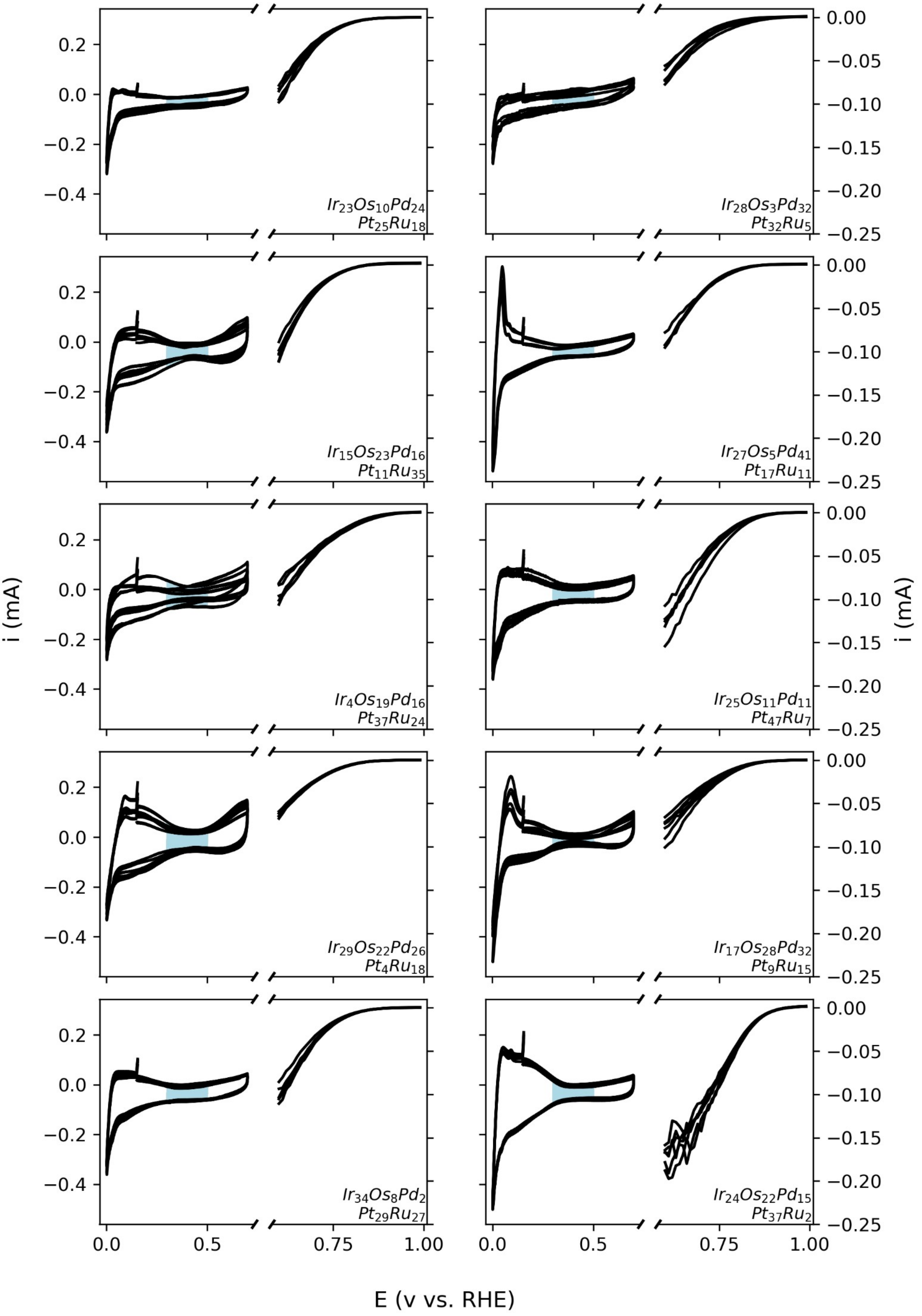

$Ir_{23}Os_{10}Pd_{24}Pt_{25}Ru_{18}$
$Ir_{28}Os_{3}Pd_{32}Pt_{32}Ru_{5}$
$Ir_{15}Os_{23}Pd_{16}Pt_{11}Ru_{35}$
$Ir_{27}Os_{5}Pd_{41}Pt_{17}Ru_{11}$
$Ir_{4}Os_{19}Pd_{16}Pt_{37}Ru_{24}$
$Ir_{25}Os_{11}Pd_{11}Pt_{47}Ru_{7}$
$Ir_{29}Os_{22}Pd_{26}Pt_{4}Ru_{18}$
$Ir_{17}Os_{28}Pd_{32}Pt_{9}Ru_{15}$
$Ir_{34}Os_{8}Pd_{2}Pt_{29}Ru_{27}$
$Ir_{24}Os_{22}Pd_{15}Pt_{37}Ru_{2}$
i (mA)
i (mA)
E (v vs. RHE)
0.2
0.0
−0.2
−0.4
0.00
−0.05
−0.10
−0.15
−0.20
−0.25
0.0
0.5
0.75
1.00

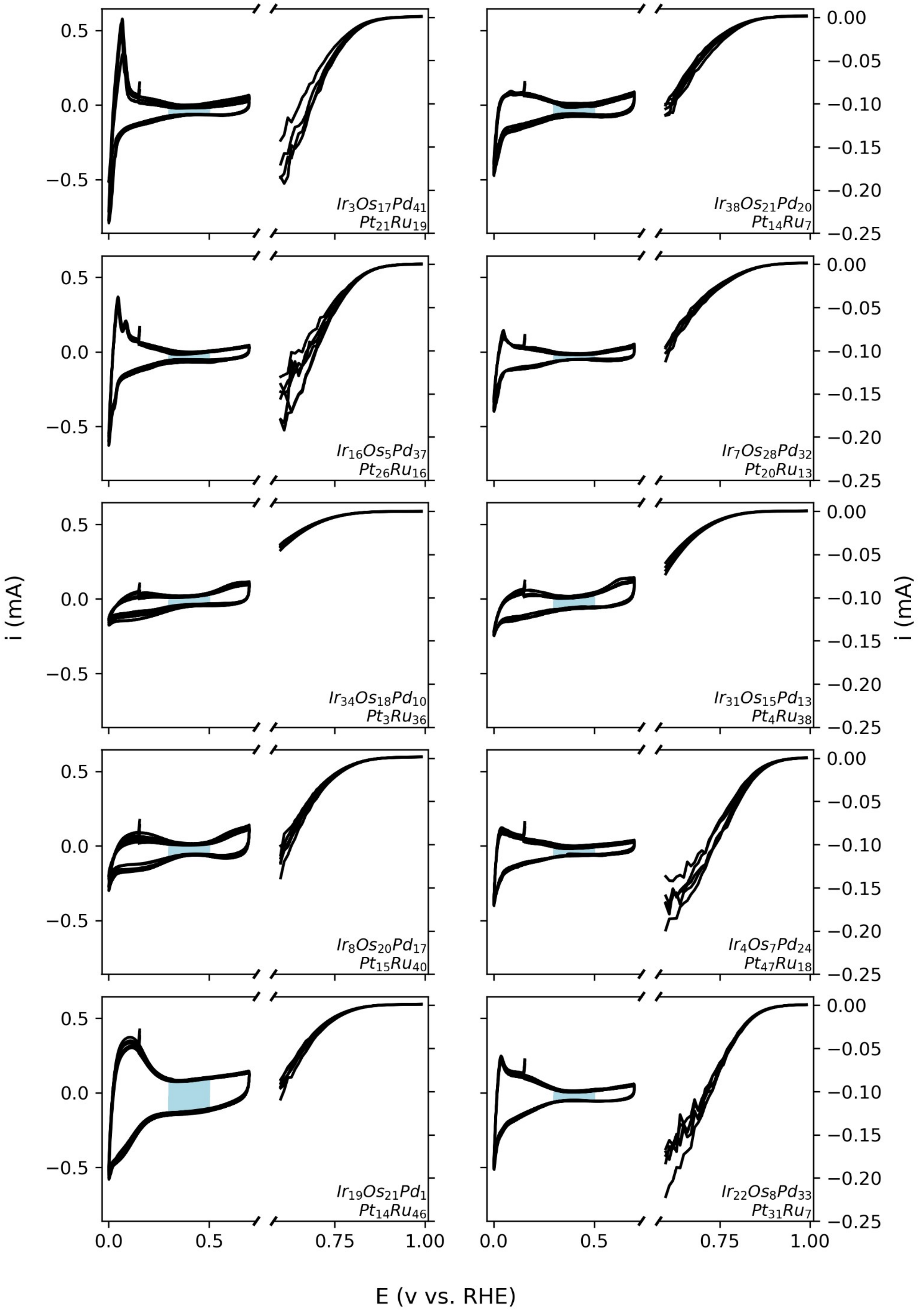

i (mA)
i (mA)
E (v vs. RHE)
$Ir_3Os_{17}Pd_{41}Pt_{21}Ru_{19}$
$Ir_{38}Os_{21}Pd_{20}Pt_{14}Ru_7$
$Ir_{16}Os_5Pd_{37}Pt_{26}Ru_{16}$
$Ir_7Os_{28}Pd_{32}Pt_{20}Ru_{13}$
$Ir_{34}Os_{18}Pd_{10}Pt_3Ru_{36}$
$Ir_{31}Os_{15}Pd_{13}Pt_4Ru_{38}$
$Ir_8Os_{20}Pd_{17}Pt_{15}Ru_{40}$
$Ir_4Os_7Pd_{24}Pt_{47}Ru_{18}$
$Ir_{19}Os_{21}Pd_1Pt_{14}Ru_{46}$
$Ir_{22}Os_8Pd_{33}Pt_{31}Ru_7$

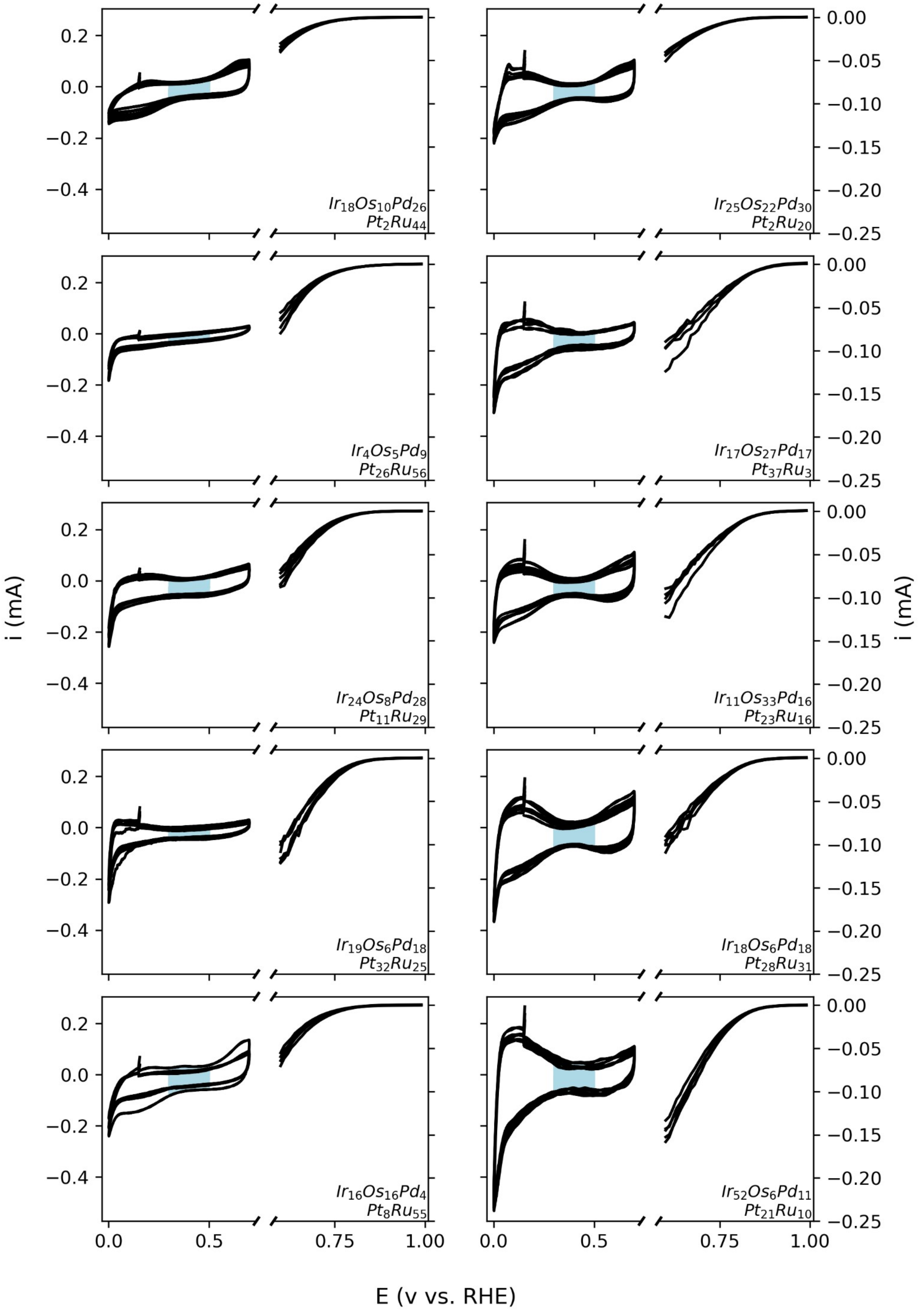

i (mA)
i (mA)
E (v vs. RHE)
$Ir_{18}Os_{10}Pd_{26}Pt_{2}Ru_{44}$
$Ir_{25}Os_{22}Pd_{30}Pt_{2}Ru_{20}$
$Ir_{4}Os_{5}Pd_{9}Pt_{26}Ru_{56}$
$Ir_{17}Os_{27}Pd_{17}Pt_{37}Ru_{3}$
$Ir_{24}Os_{8}Pd_{28}Pt_{11}Ru_{29}$
$Ir_{11}Os_{33}Pd_{16}Pt_{23}Ru_{16}$
$Ir_{19}Os_{6}Pd_{18}Pt_{32}Ru_{25}$
$Ir_{18}Os_{6}Pd_{18}Pt_{28}Ru_{31}$
$Ir_{16}Os_{16}Pd_{4}Pt_{8}Ru_{55}$
$Ir_{52}Os_{6}Pd_{11}Pt_{21}Ru_{10}$

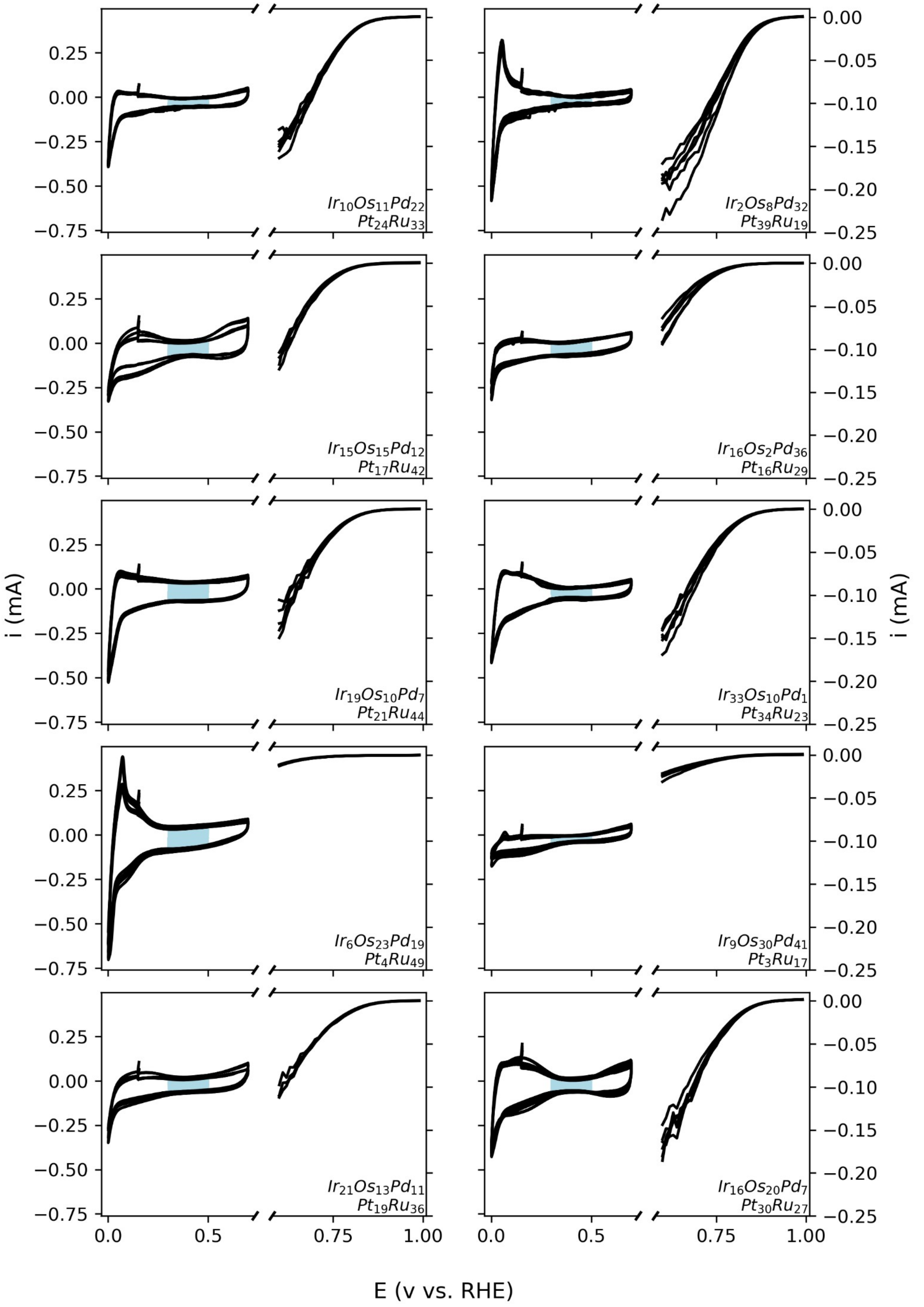

Fi
g S12. Cyclic voltammograms of the nanoparticles and the ORR measurements. The measurements were carried out

in 0.1 M H_2SO_4. **(Left side)** The CV of the nanomaterials recorded at 100 mV/s. The coloured filled area corresponds to the region where the capacitance was extracted. **(Right side)** ORR measurements of the nanomaterials with the EDX composition. The activities were measured in potential steps of 10 mV from 1 V to 0.6 V each lasting for 20 seconds. The data point shown here is the average of the last 17 seconds of each potential hold.

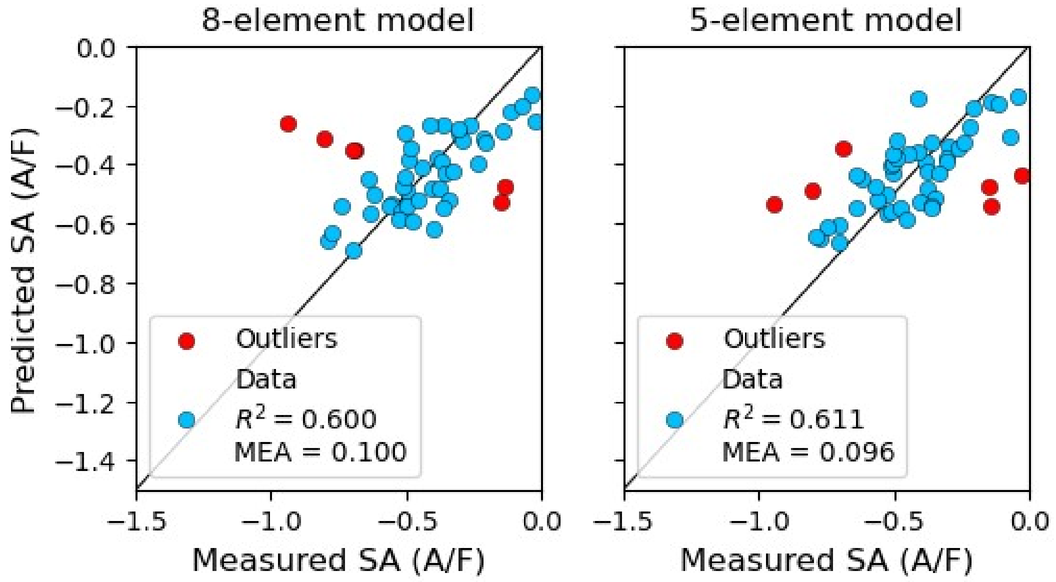

V. Model comparison and bias correction

Fig. S13. The R^2 value in Au-Os-Pd-Pt-Ru space. R^2 recalculated without outliers using the bias corrected 8-element model and the 5-element LOOCV data points. The outliers were included in the 5-element model LOOCV.

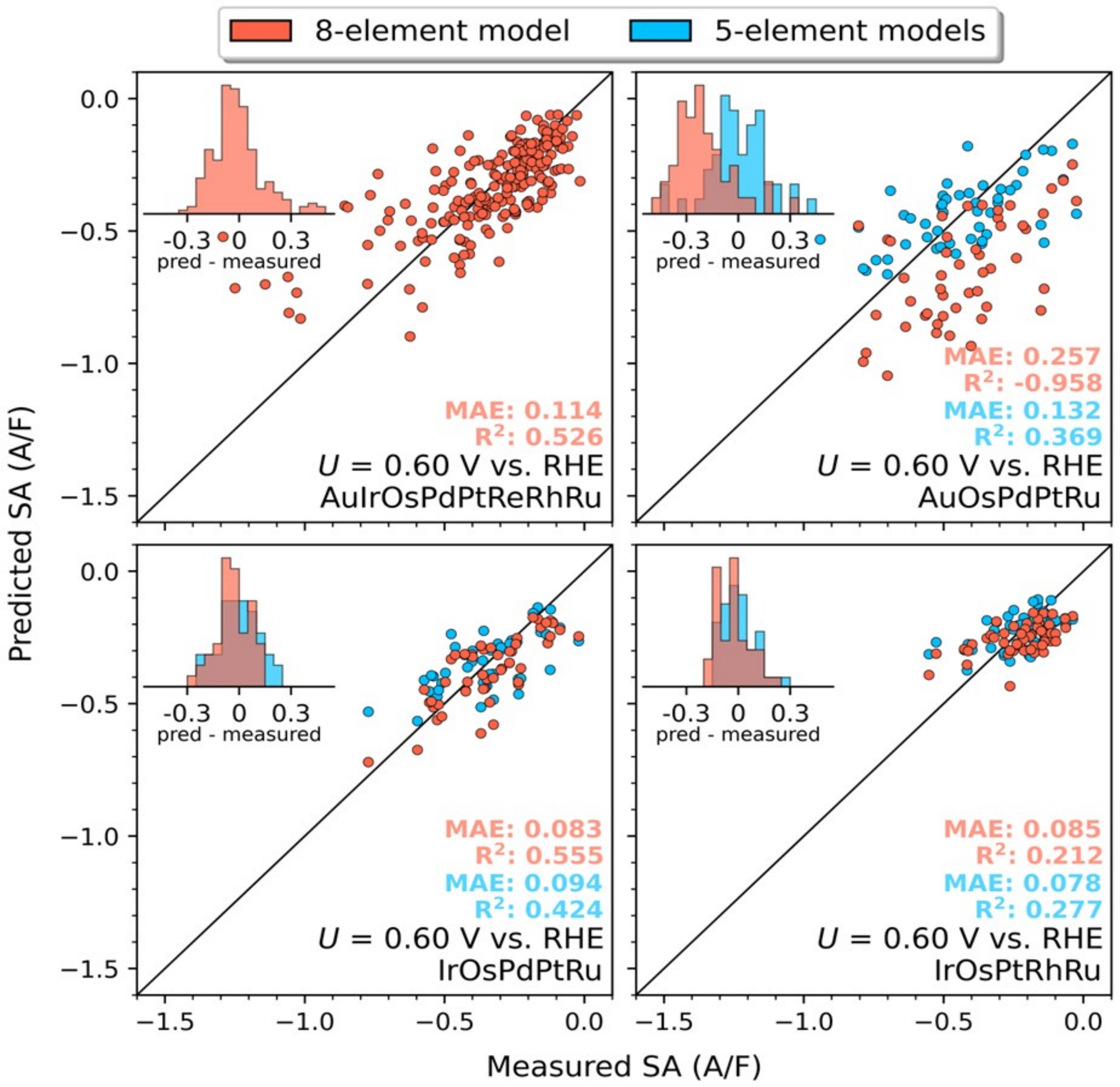

Fig. S14. Parity plots without bias correction. Predicted vs. measured current per capacitance for each of the four investigated alloy systems. The prediction was evaluated using the leave-one-out cross-validation. In addition, the subplots with the 5-element models show how the 8-element model is predicting the same data points. The 8-element model is observed to perform similarly to the 5-element model for Ir-Os-Pd-Pt-Ru and Ir-Os-Pd-Rh-Ru having approximately the same error metrics. The 8-element model, however, show a systematic bias for Au-Os-Pd-Pt-Ru, resulting in poor prediction in absolute terms, but fair in relative terms. Multiplying by a constant factor corrects this systematic bias as shown in Fig. 2B-E in the manuscript. The reason for the systematic shift in this part of the composition space is unknown, but under sampling of this region by the 8-element experiments is likely to play a role.

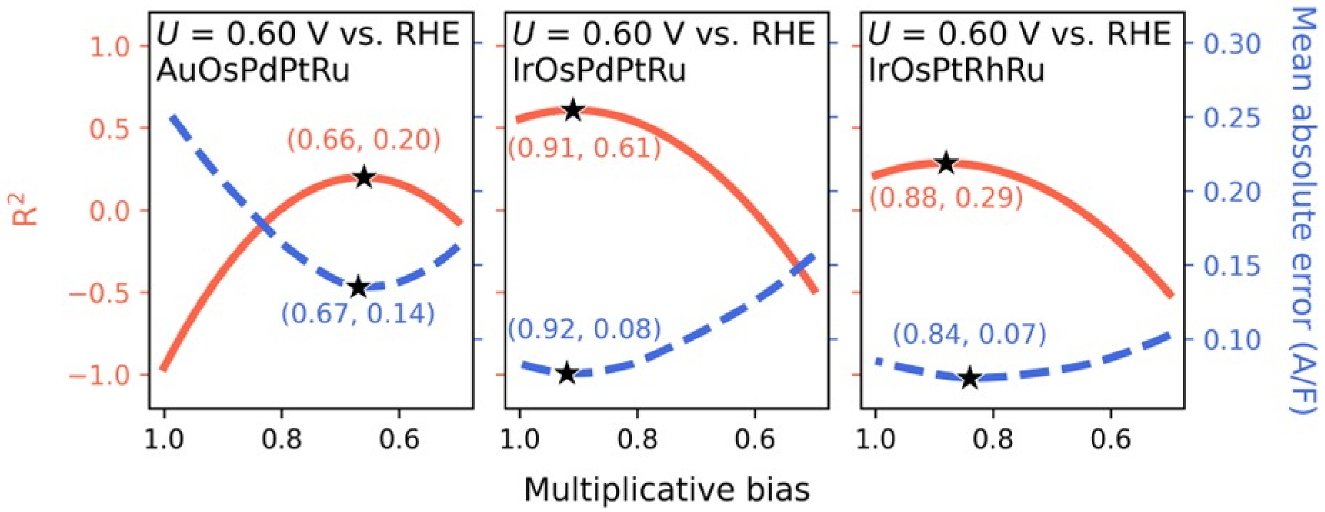

Fig. S15. Selection of optimal multiplicative bias. Multiplicative bias of the 8-element model vs. coefficent of determination (R^2, red solid lines) and mean absolute error (blue, dashed lines) for the three 5-element alloy systems. The star markers indicate the optimal values of the multiplicative bias. The R^2 value assumes its maximum value for a multiplicative bias of 0.66, 0.91, and 0.88 for Au-Os-Pd-Pt-Ru, Ir-Os-Pd-Pt-Ru, and Ir-Os-Pt-Rh-Ru respectively which has been used to produce the 8-element predictions in Fig. 2B-E in the manuscript.

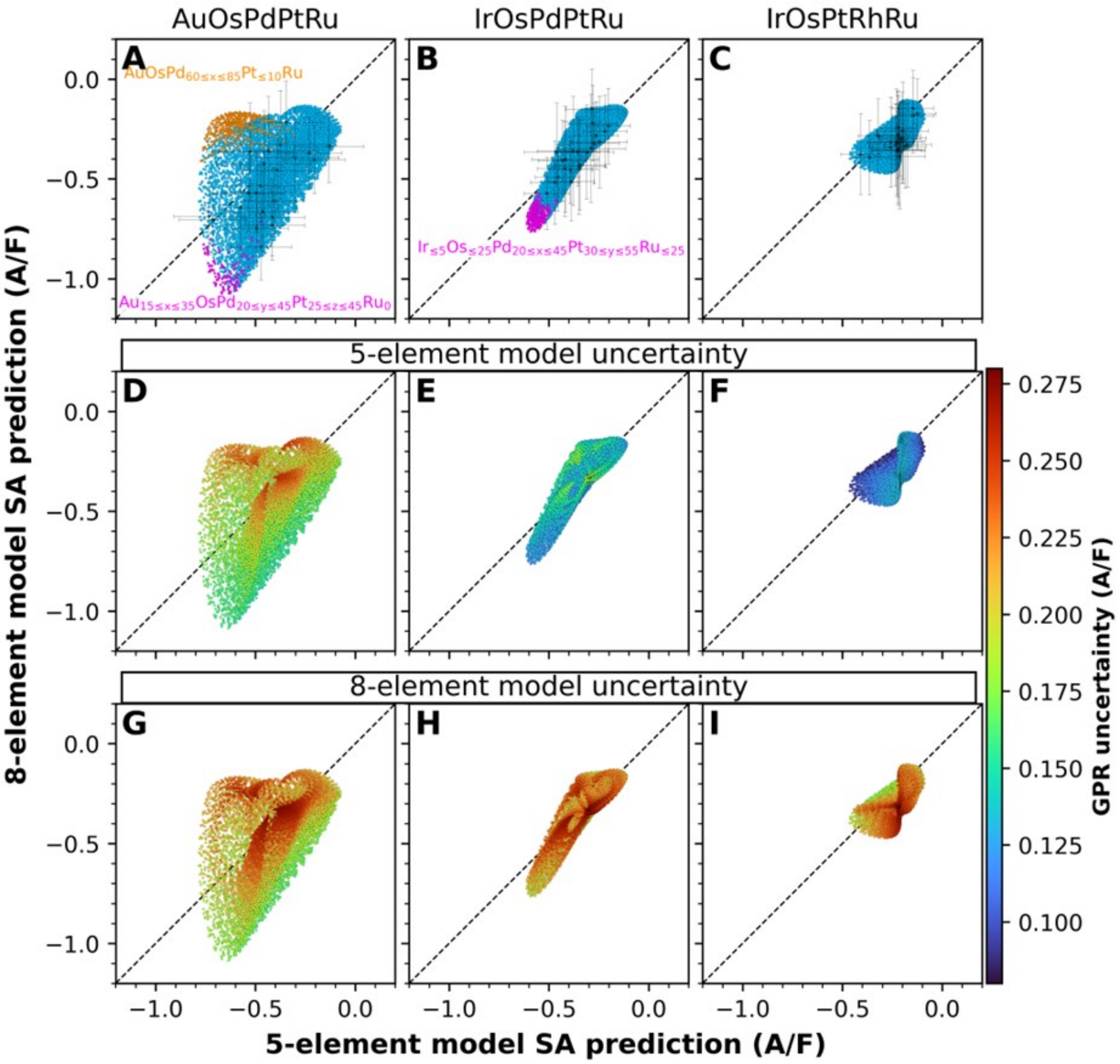

Fig. S16. Predictions in all of the quinary composition spaces without bias correction. (**A**-**I**) Parity plots comparing the prediction of the 8- and 5-element models on all compositions of the quinary composition spaces taken in steps of 5 at.%. The 8-element predictions have not been corrected for any bias. Each column corresponds to a quinary alloy system as labelled at the top. (**A**-**C**) Regions of the composition space have been highlighted where the predictions between the 8-element and 5-element models differ: The 8-element model overestimates the activity of compositions with intermediate Au, Pd, and Pt concentrations when ruthenium is absent (magenta points in A) as well as Pd-Pt rich compositions in Ir-Os-Pd-Pt-Ru (magenta points in B), and underestimates certain Pd-rich compositions in Au-Os-Pd-Pt-Ru (orange points in A). Error bars corresponding to the uncertainty of the Gaussian process regressor (GPR) have been shown for a random selection of points. (**D**-**F**) Same plots with the colour coding illustrating the GPR uncertainty of the 5-element models (the *x* direction error bars in A-C). (**G**-**I**) Same plots with the colour coding illustrating the GPR uncertainty of the 8-element model (the *y* direction error bars in A-C). The GPR uncertainties of the 8-element model are generally larger than for the 5-element models, and the uncertainties are observed to show correlation.

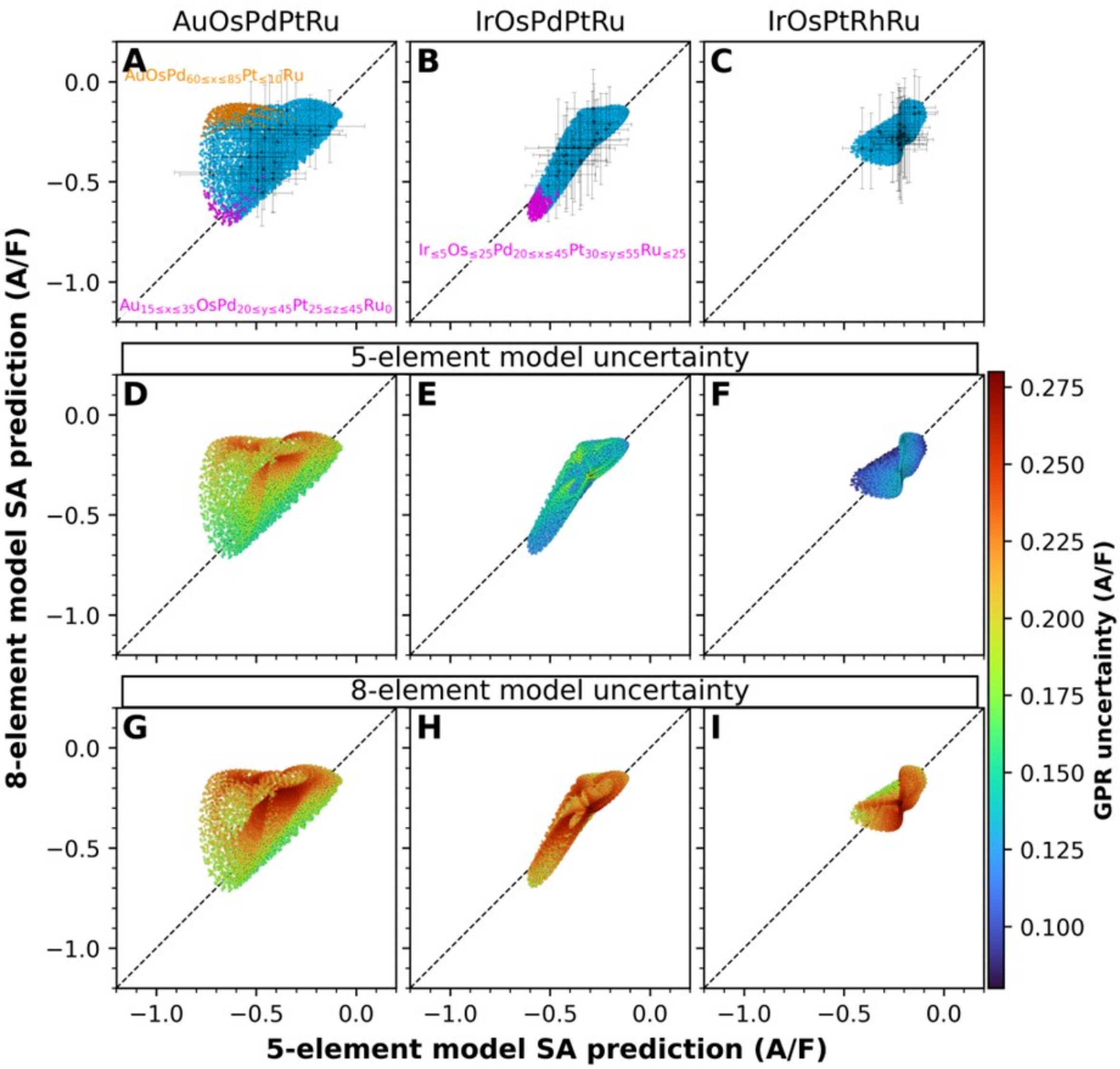

Fig. S17. Predictions in all of the quinary composition spaces with bias correction. (**A**-**I**) Parity plots comparing the prediction of the 8- and 5-element models on all compositions of the quinary composition spaces taken in steps of 5 at.%. The 8-element predictions have been corrected with a constant multiplicative factor of 0.66, 0.91, and 0.88 for Au-Os-Pd-Pt-Ru, Ir-Os-Pd-Pt-Ru, and Ir-Os-Pt-Rh-Ru respectively (from Fig. S14). Each column correspond to a quinary alloy system as labeled in the top. (**A**-**C**) Regions of composition space identical to those in Fig. S15 have been highlighted. Error bars corresponding to the uncertainty of the Gaussian process regressor (GPR) have been shown for a random selection of points. (**D**-**F**) Same plots with the color coding illustrating the GPR uncertainty of the 5-element models (the *x* direction error bars in A-C). (**G**-**I**) Same plots with the color coding illustrating the GPR uncertainty of the 8-element model (the *y* direction error bars in A-C).

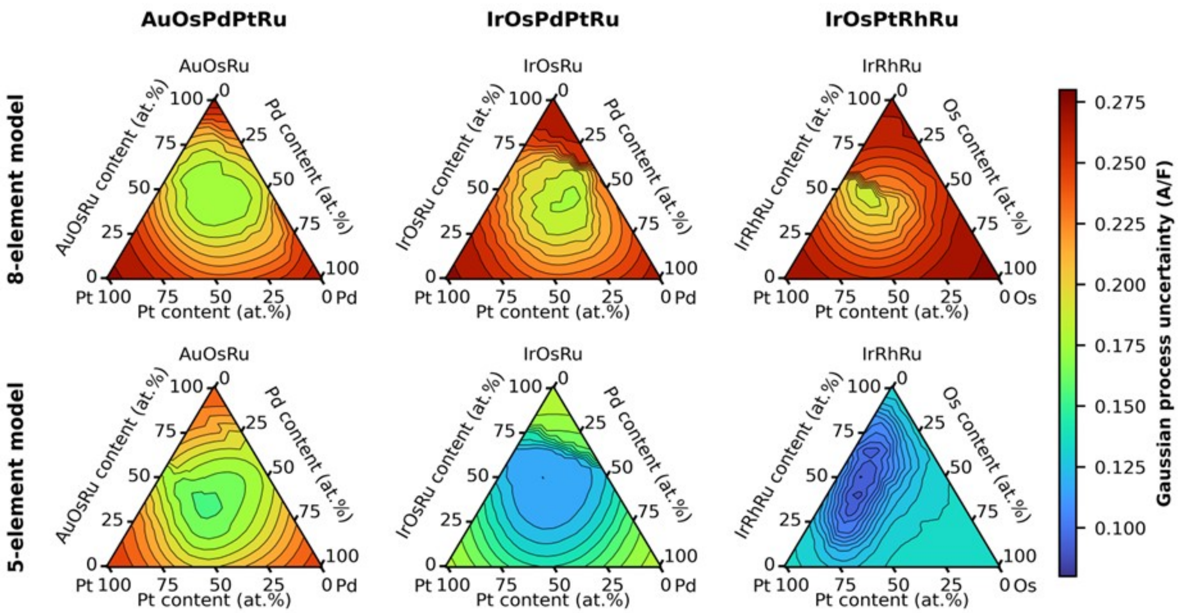

Fig S18. Model uncertainties around the optima. Gaussian process regression uncertainties around the optimal compositions in each 5-element composition space for the 5-element and 8-element models. The pseudo-ternary plots are equivalent to those in Fig. 3B-G in the manuscript in the sense that the uncertainties of the most active compositions are shown on-top of other compositions at the same position in the pseudo-ternary plots. As expected, model uncertainties are observed to increase as the composition approaches binary and unary alloys.

VI. Confirming the DFT optimum.

Experimental Details

Nanoparticle Electro Deposition

The 5-element screening identified the region of interest for ORR catalysts. In this region of interest 5 nanoparticle compositions were selected for further study. These nanoparticles were synthesized using an electrodeposition method, which provides a better control over the wet-chemical approach used for the screening. The nanoparticles were deposited on a 5mm glassy carbon rotating disk electrode, in a three-electrode setup. In this, a Ag/AgCl reference electrode and a glassy carbon counter electrode were employed. The deposition was carried out 12 mL 0.1 M NaCl (Merck, Suprapur, dissolved in MiliQ water) containing at 500 uM stoichiometric precursor solution ($HAuCl_4$, Sigma Aldrich, 99.9%; H_2PtCl_6, Sigma Aldrich, 99.9%; $(NH_4)_2PdCl_6$, Sigma Aldrich, 99.95%). The solution was degassed with Ar. To ensure a homogeneous mixture and magnetic field, the rotating disk electrode was rotated at 300 RPM while the solution was stirred with a magnetic bar.

The RDE was inserted at a controlled potential of 1 V vs. Ag/AgCl. Once inserted, a nucleation pulse of -0.5 V vs. Ag/AgCl was applied that lasted for 2 seconds. Then, the particles were grown by alternating the potential between 0.75 and -0.45 V vs. Ag/AgCl for 1 minute, with each potential hold lasting for 0.01 second. Using the same bath, this procedure was repeated 4 times to produce samples for electrochemical activity measurements. Lastly, a 5th deposition was performed to produce particles for microscopic characterization. Its deposition time was extended to 5 minutes to produce sufficient nanomaterial.

Nanoparticle Characterization

The nanoparticles were detached from the tip into EtOH (VWR, AnalaR NORMAPUR) by bath sonication. After they were transfer to a TEM Cu grid. The composition of these nanoparticles were evaluated using energy dispersive x-ray spectroscopy in a GeminiSEM450 (Zeiss) equipped with the EDS Photodetector Ultim Max 65 (Oxford instruments). The working distance was 8.5 mm, the accelerating voltage was 20 keV, and the probe current was 100 pA. High resolution images were recorded on a FEI Tecnai F20 microscope, which was equipped with LaB6 FEG gun. FEI Ceta CMOS (4000x4000 pixels) was used to collect the bright field phase contrast images from the NPs.

Nanoparticle Electrochemistry

The electrochemistry of the nanoparticles was measured following the best practices for Pt based ORR measurements[11.] All activity measurements were conducted in a three-electrode cell with a RDE working electrode, a RHE reference electrode and a Pt counter electrode. The counter electrode was separated from the working compartment by a glass frit. 0.1 M H2SO4 (Merck, Suprapur dissolved in MiliQ) was used as electrolyte to be consistent with the screening experiments. The solution resistance of the set-up was compensated to produce a residual solution resistance of 8 Ohms, which was recorded online using single frequency impedance spectroscopy at 10 kHz and 5 mV amplitude. First, the particles were electrochemically polished by recording 200 cyclic voltammograms (CVs) at 200 mV/s between 0.05 and 1.2 V vs RHE in Argon, followed by characterization CVs at 10, 50, 100, 200 mV/s in the same potential window. Also, a background CV was recorded at 10 mV/s between 0.05 and 1.0 V vs. RHE. After, the electrode was held at 0.05 V vs. RHE while being poisoned with CO. Subsequently, the system was purged for 20 minutes with Ar and the CO was removed from the surface by recording 3 CVs between 0.05 and 1.2 V vs. RHE. Then, the system was saturated with O_2 for 20 minutes, while the electrode was held at 1.0 V vs. RHE. The oxygen reduction reaction activity was recorded at 1600, 400, 900, 1600 and 2500 RPM in this listed order. At each rotation 2 CVs between 0.05 and 1.0 V vs. RHE at 10 mV/s were recorded. Between changing the rotation rate, the

electrode was reactivated by recording 10 CVs between 0.05 and 1.2 V vs. RHE at 200 mV/s. Lastly, H_2 was flown into the system while 5 CVs between -0.05 and 1.2 V vs RHE at 2500 RPM with a scan rate of 50 mV/s were recorded. The last CV was used to measure the RHE potential offset. In case of pure Pd nanoparticles the last measurement in H_2 was repeated using a Pt disk.

Pt(111) Measurements

The single crystal electrochemistry measurements were performed in a three electrode set-up with a Pt counter electrode and a RHE reference electrode in 0.1 M H2SO4 (Merck, Suprapur dissolved in MiliQ). As the measurements were performed in a hanging meniscus configuration a steering bar was added to the cell to provide mass transport. The Pt(111) crystal was prepared by butane-flame annealing and cooling in CO atmosphere. After, the surface was conditioned by measuring CVs between 0.05 and 0.85 V vs. RHE at 50 mV/s until a stable CV was obtained. After three CVs were recorded at 50 mV/s and three at 10 mV/s. Subsequently, the solution was saturated with O_2, and three CVs were recorded between 0.05 and 0.85 V vs. RHE at 50 mV/s and 10 mV/s while the bar was rotating at 1250 RPM. In addition, steady state measurements were conducted by stepping the potential down from 0.9 V vs. RHE to 0.75 V vs. RHE with steps of 10mV lasting for 20 seconds. Lastly the reference shift was measured by flowing H_2 in the system while measuring CVs between -0.05 and 0.4 V vs. RHE at 10 mV/s.

Figure 5D construction

The kinetic current for the nanoparticle and Pt(111) in Figure 5D was constructed using Eq. S8. In this Equation j_l is the diffusion limited current, j is the total current and j_k is the kinetic current. In case of the nanoparticles, j was the forward scan at 1600 RPM and 10 mV/s corrected for the blank cyclic voltammogram, solution resistance, reference shift and normalized to the CO stripping charge. j_l was assumed to be the average current in the diffusion limited plateau after the corrections and normalizations. Subsequently, the solid line reports the average j_k obtained for the multiple samples whereas the filled area corresponds to the standard deviation in the j_k.

$$j_k = \left(\frac{1}{j} - \frac{1}{jl}\right)^{-1} \quad \text{(Eq. S8)}$$

For Pt(111) a similar procedure was used. However, as the forward and backward scans show no hysteresis both scans were considered in the calculation of the average current and the error. The spikes present in the reported data result from the measurement being a hanging meniscus with a steering bar instead of a RDE.

Pt Nanoparticles Results

Electrochemistry

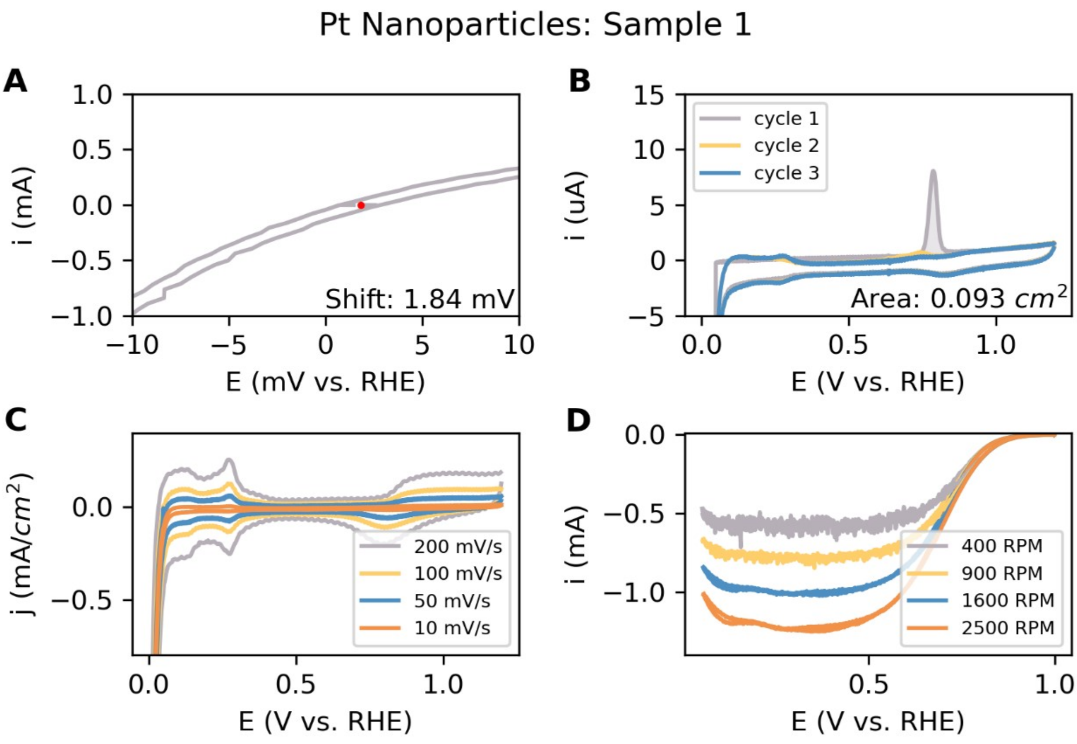

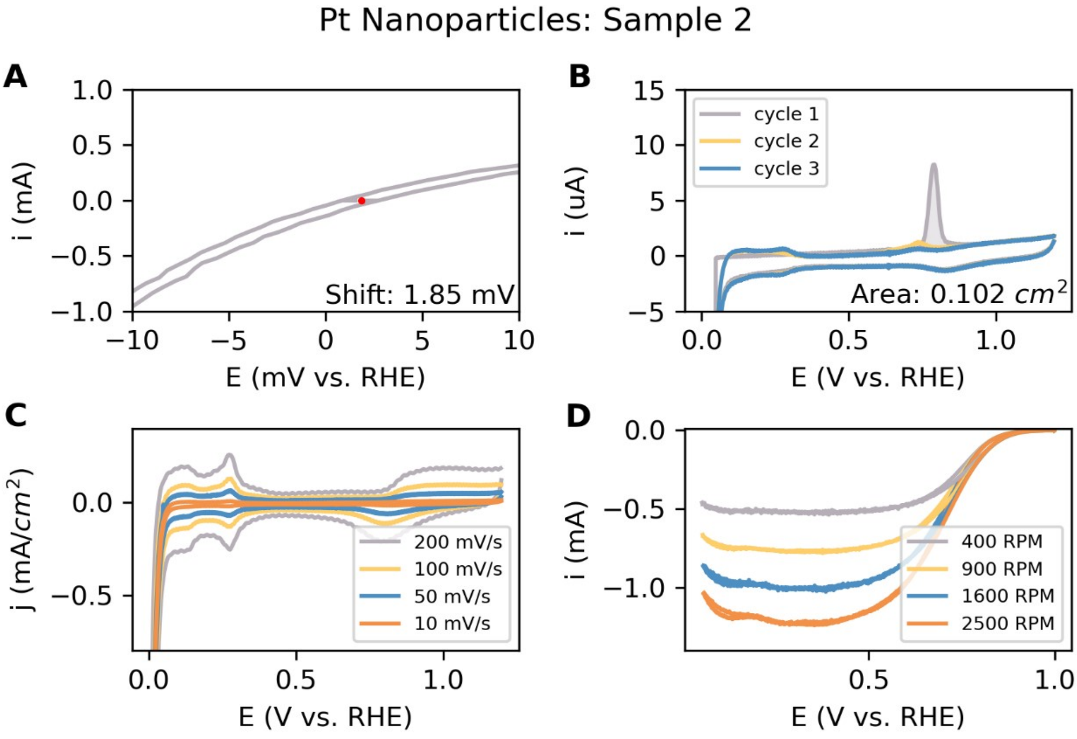

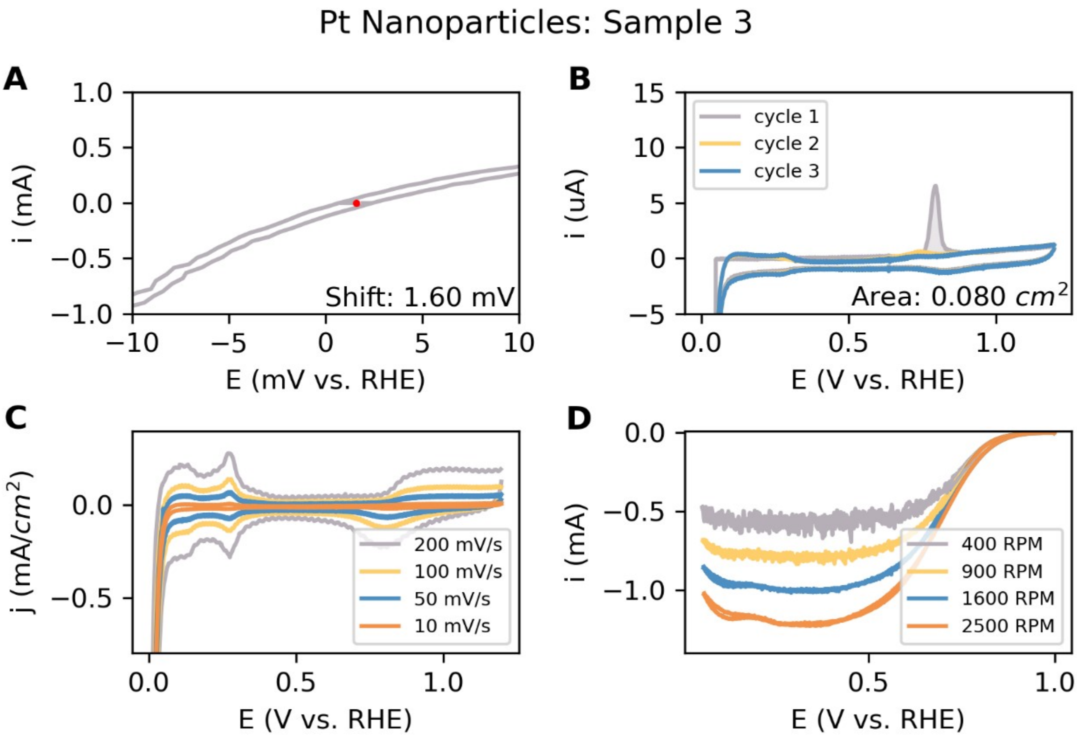

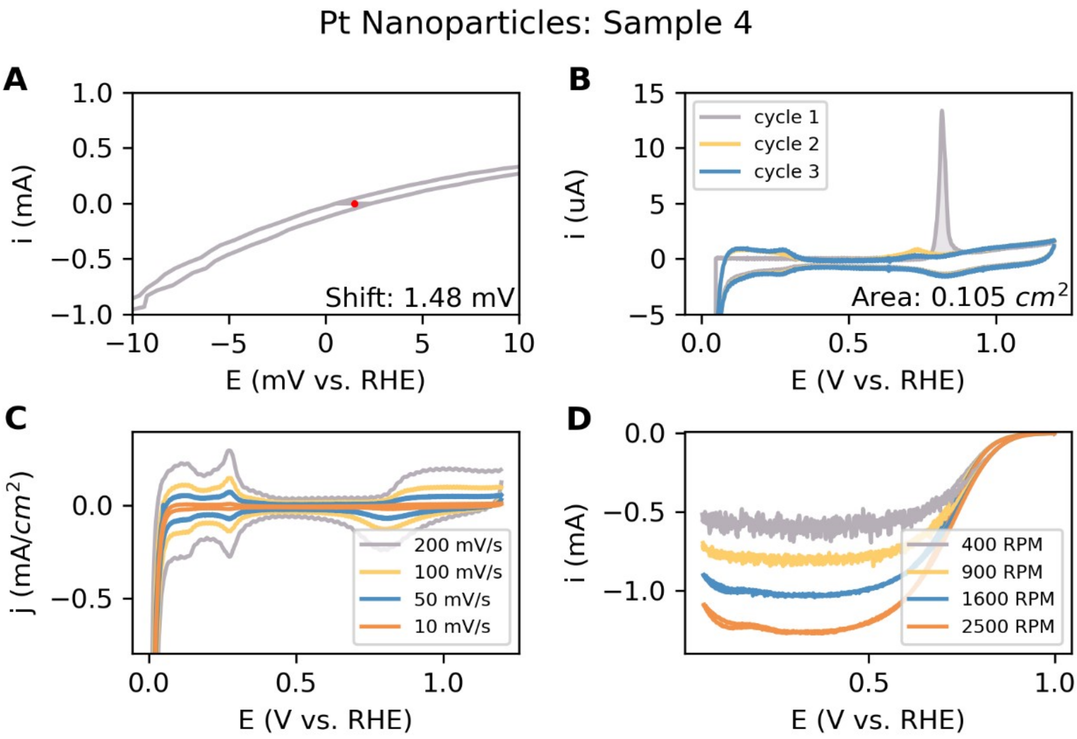

Fig S19. Pt nanoparticle electrochemistry. A) The reference shift of the RHE electrode. **B)** The CO stripping experiment to determine the surface area. The integrated area is filled. **C)** CVs at different scan speeds. **D)** The ORR activity measurements at different rotation rates at 10 mV/s. The characterization CV in Ar was subtracted from these measurements. Also, these measurements are corrected for the solution resistance and reference shift.

Characterization

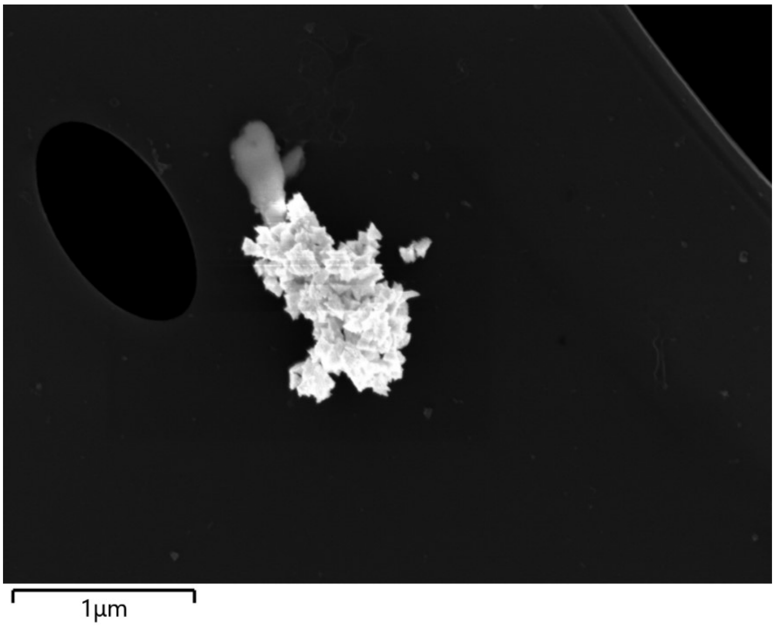

Fig S20. SEM characterization of Pt nanoparticles.

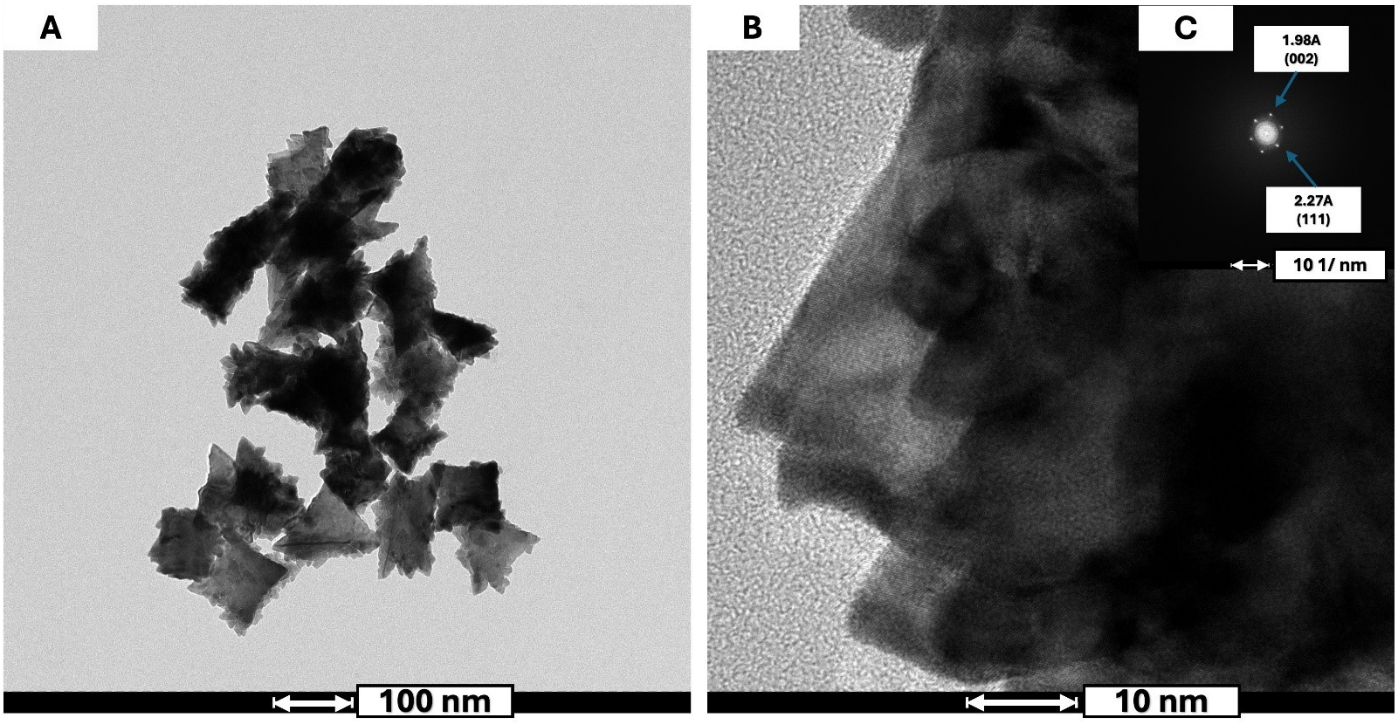

Fig S21. High resolution imaging of Pt nanoparticles. A) Bright Field Images. **B)** The high-resolution transmission electron microscopy image. **C)** shows the corresponding fast Fourier transform of B.

$Au_{10}Pt_{90}$ Nanoparticles Results

Electrochemistry

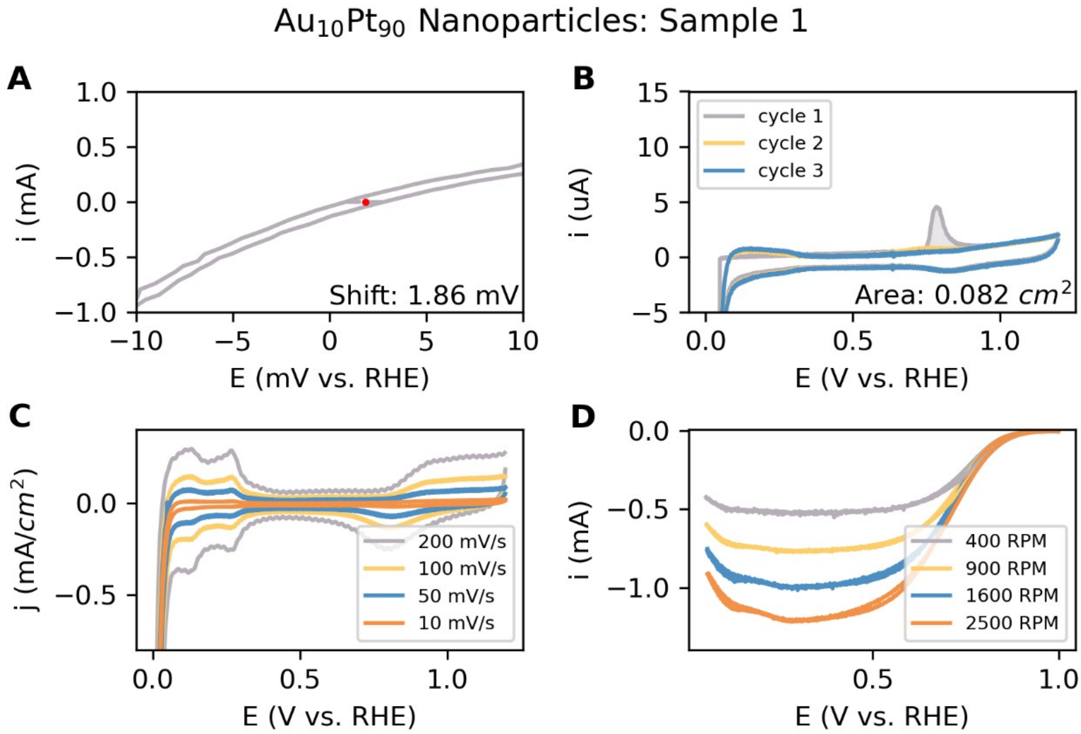

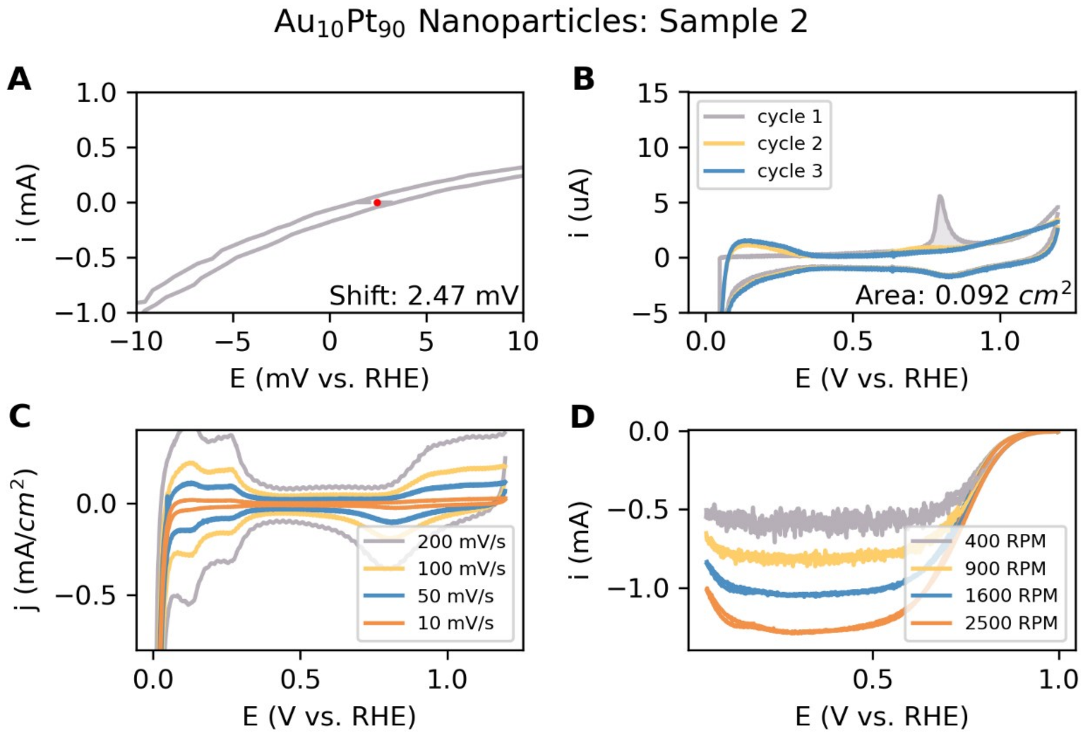

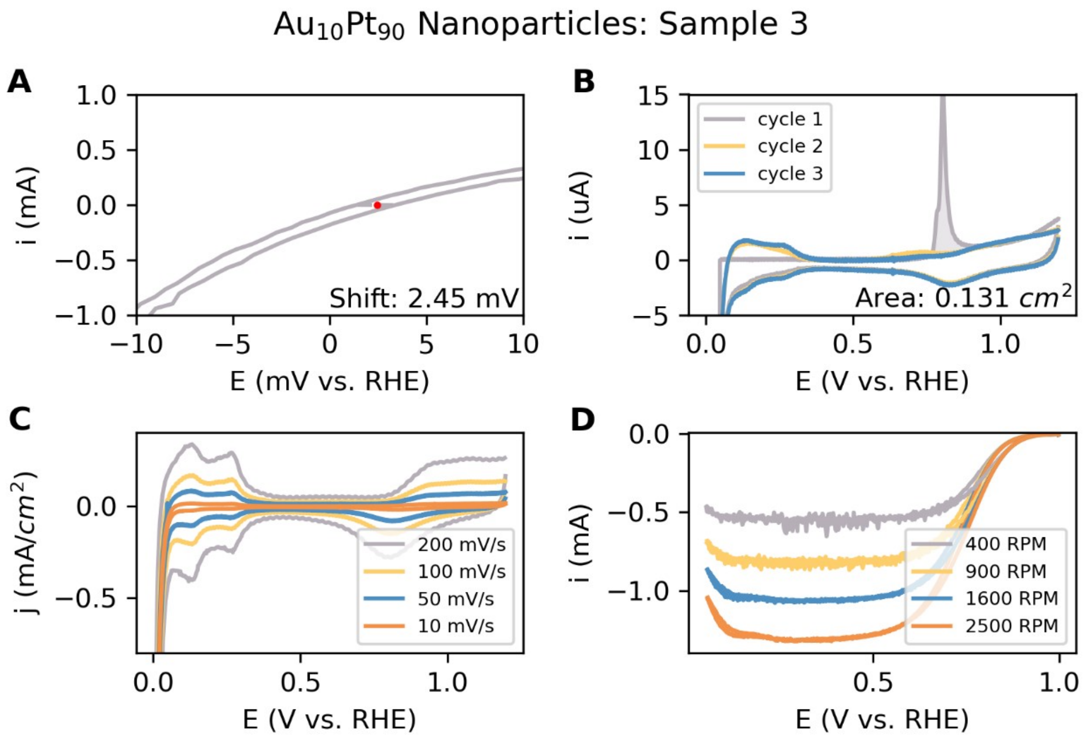

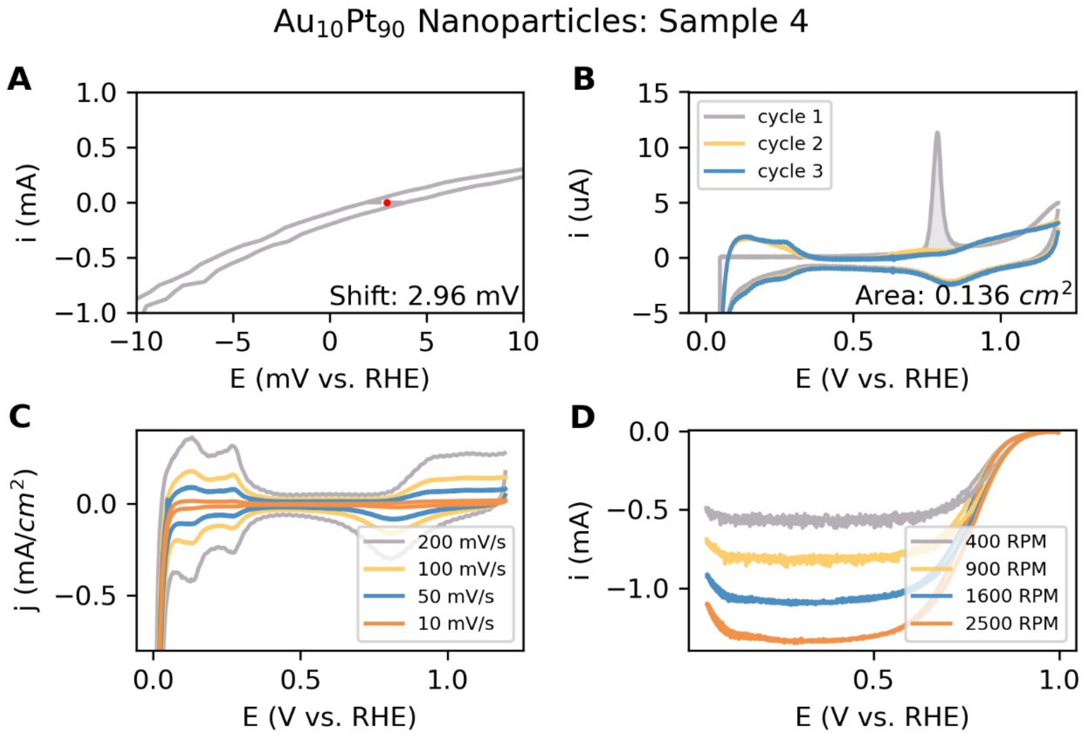

Fig S22. $Au_{10}Pt_{90}$ electrochemistry measurements. A) The reference shift of the RHE electrode. **B)** The CO stripping experiment to determine the surface area. The integrated area is filled. **C)** CVs at different scan speeds. **D)** The ORR activity measurements at different rotation rates at 10 mV/s. The characterization CV in Ar was subtracted from these measurements. Also, these measurements are corrected for the solution resistance and reference shift.

Characterization

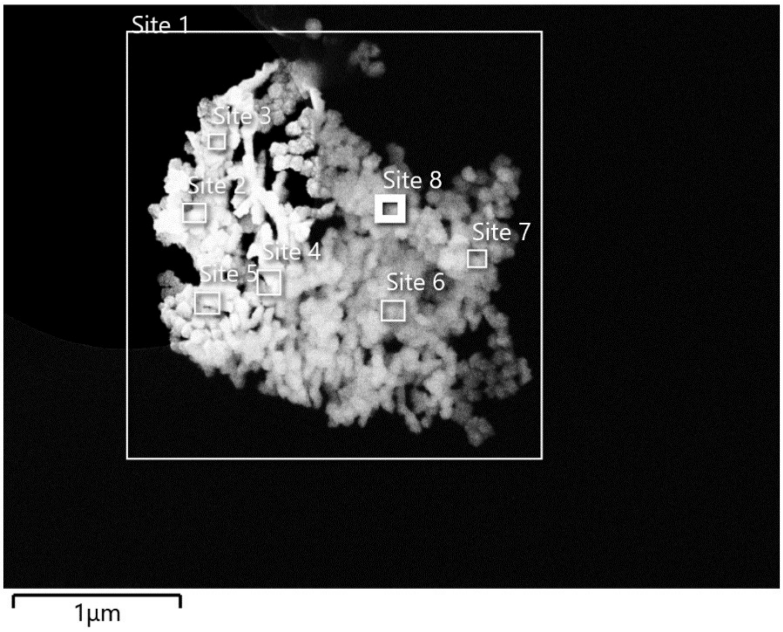

Site 1	$Au_{11.8}Pt_{88.2}$
Site 2	$Au_{13.3}Pt_{86.7}$
Site 3	$Au_{13.6}Pt_{86.4}$
Site 4	$Au_{13.4}Pt_{86.6}$
Site 5	$Au_{14.0}Pt_{86.0}$
Site 6	$Au_{13.2}Pt_{86.8}$
Site 7	$Au_{9.7}Pt_{90.3}$
Site 8	$Au_{11.4}Pt_{88.6}$

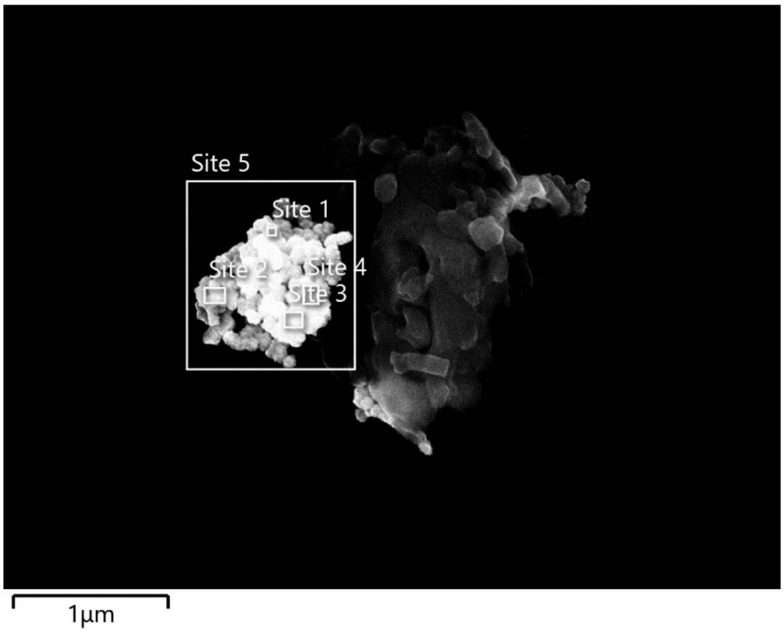

Site 1	$Au_{8.5}Pt_{91.5}$
Site 2	$Au_{9.0}Pt_{91.0}$
Site 3	$Au_{7.8}Pt_{92.2}$
Site 4	$Au_{7.8}Pt_{92.2}$
Site 5	$Au_{7.4}Pt_{92.6}$

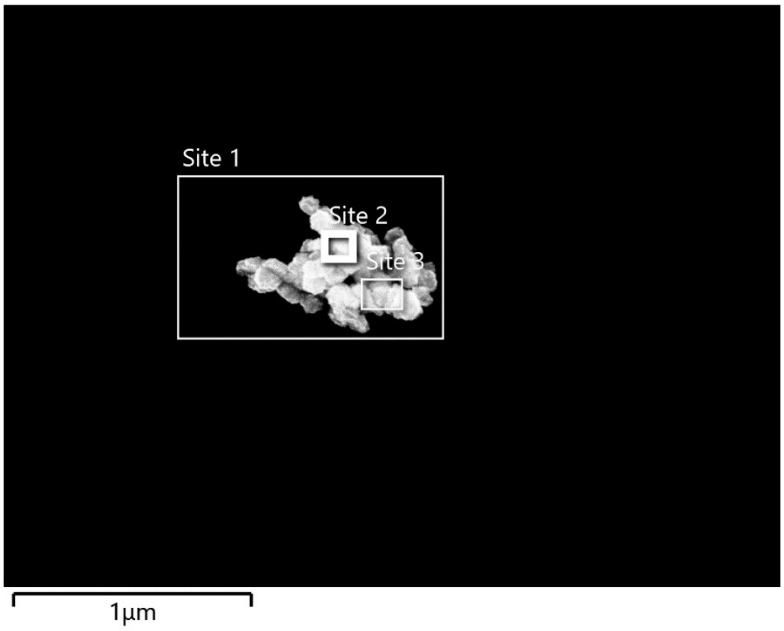

Site 1	$Au_{12.7}Pt_{87.3}$
Site 2	$Au_{13.0}Pt_{87.0}$
Site 3	$Au_{13.5}Pt_{86.5}$

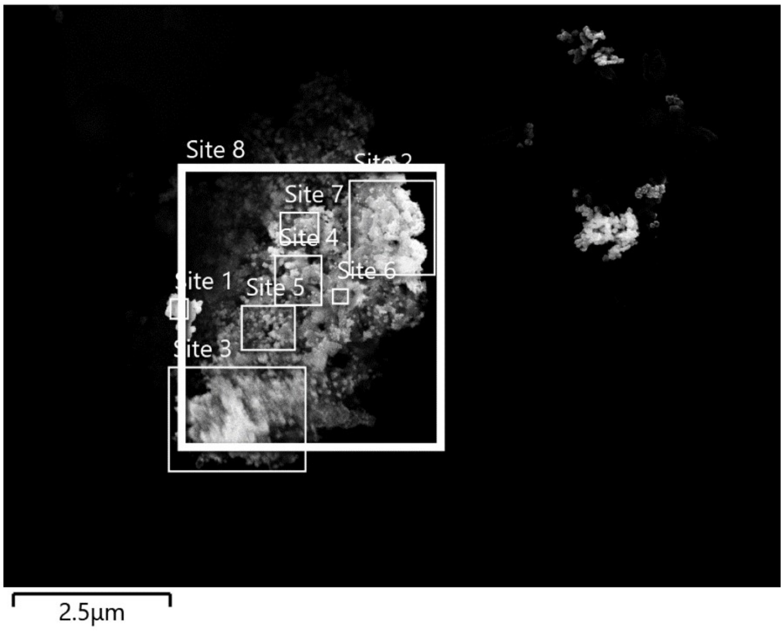

Site 1	$Au_{9.3}Pt_{90.7}$
Site 2	$Au_{1.1}Pt_{98.9}$
Site 3	$Au_{2.6}Pt_{97.4}$
Site 4	$Au_{2.4}Pt_{97.6}$
Site 5	$Au_{3.6}Pt_{96.4}$
Site 6	$Au_{2.8}Pt_{97.2}$
Site 7	$Au_{3.0}Pt_{97.0}$
Site 8	$Au_{2.9}Pt_{97.1}$

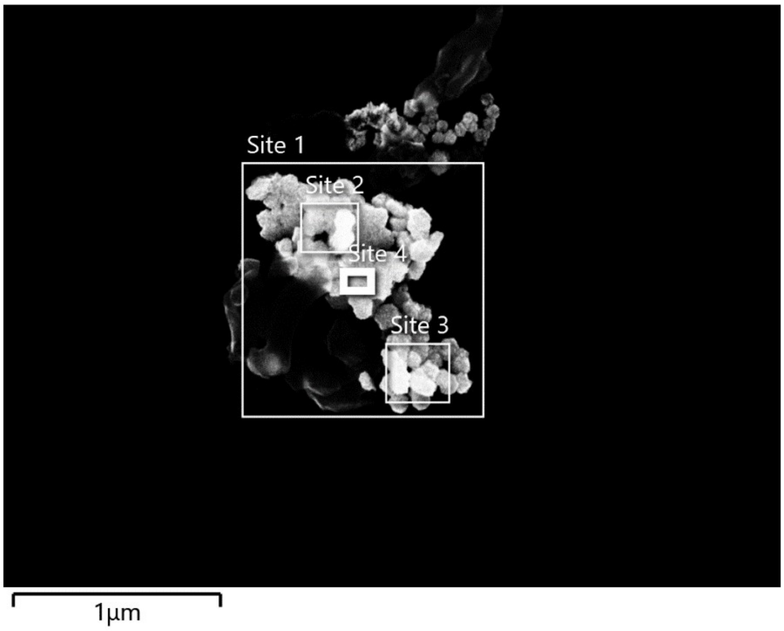

Site 1	$Au_{11.5}Pt_{88.5}$
Site 2	$Au_{11.6}Pt_{88.4}$
Site 3	$Au_{7.4}Pt_{92.6}$
Site 4	$Au_{10.3}Pt_{89.7}$

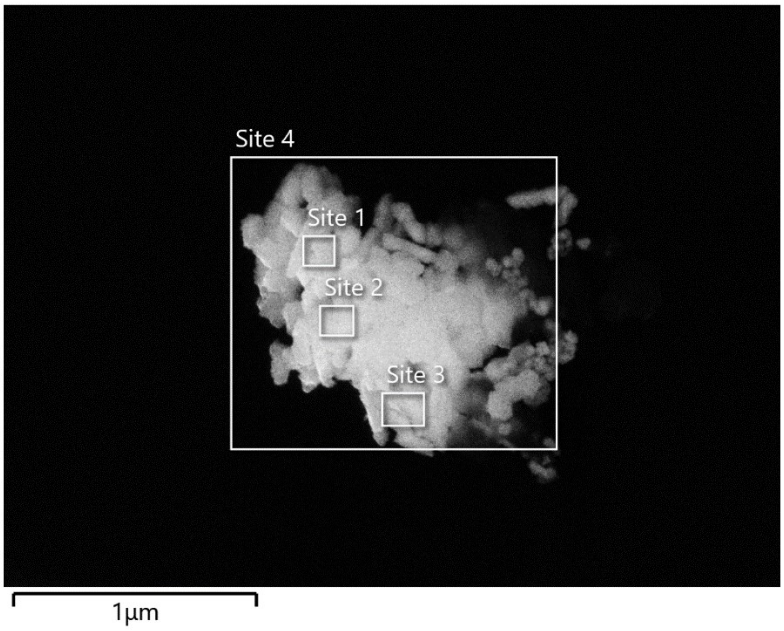

Site 1	$Au_{11.5}Pt_{88.5}$
Site 2	$Au_{11.7}Pt_{88.3}$
Site 3	$Au_{11.7}Pt_{88.3}$
Site 4	$Au_{9.6}Pt_{90.4}$

Fig S23. **SEM-EDX characterization of $Au_{10}Pt_{90}$.** The average composition across all measured particles is $Au_{9.3}Pt_{90.7}$.

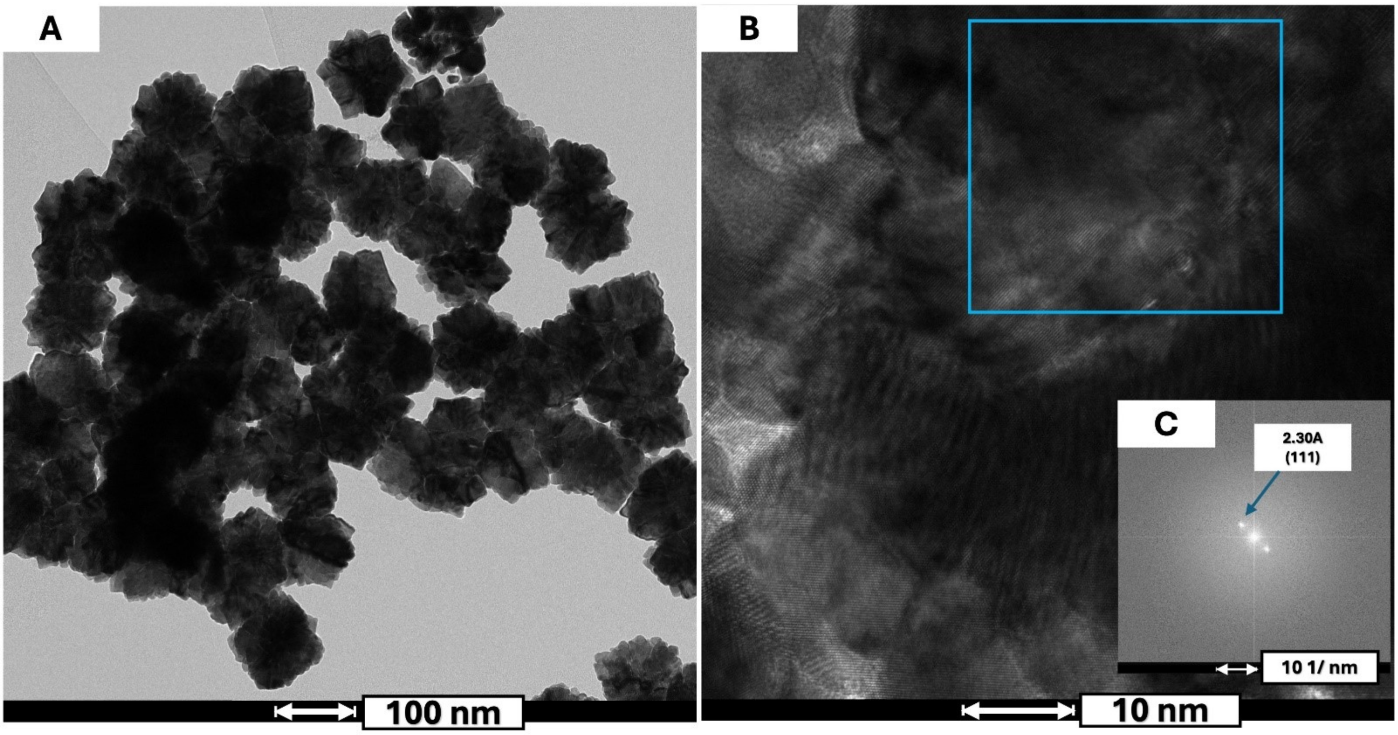

Fig S24. High resolution imaging of $Au_{10}Pt_{90}$ nanoparticles. A) Bright Field Images. **B)** The high-resolution transmission electron microscopy image. **C)** shows the corresponding fast fourier transform of B.

$Au_{15}Pd_{15}Pt_{70}$ Nanoparticle Results

Electrochemistry

$Au_{15}Pd_{15}Pt_{90}$ Nanoparticles: Sample 1

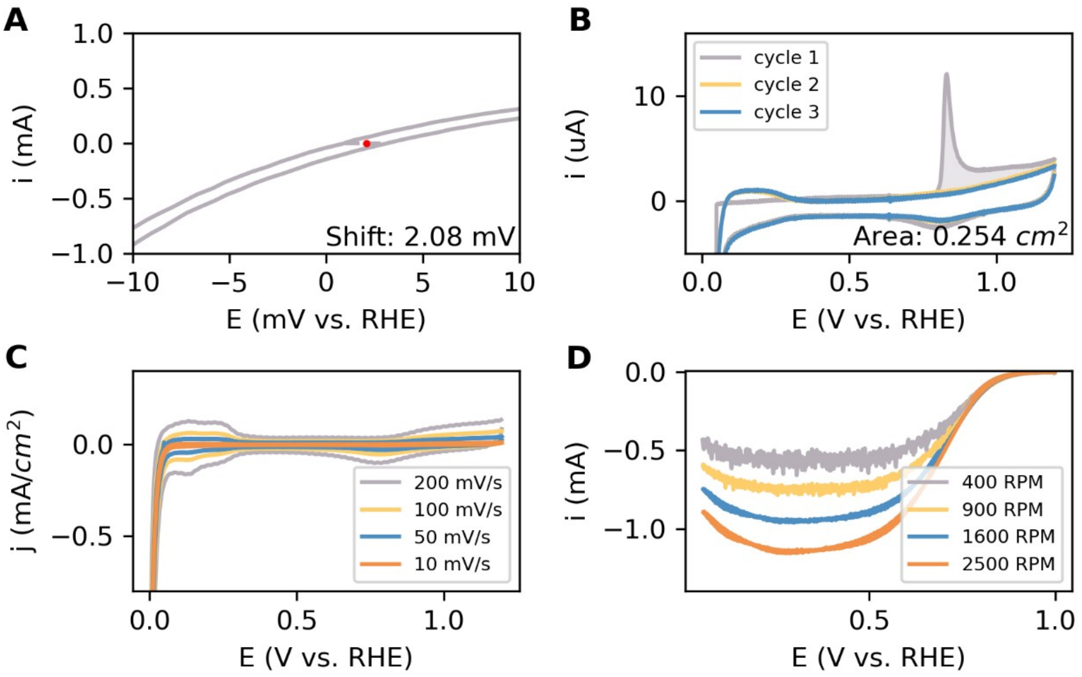

$Au_{15}Pd_{15}Pt_{90}$ Nanoparticles: Sample 2

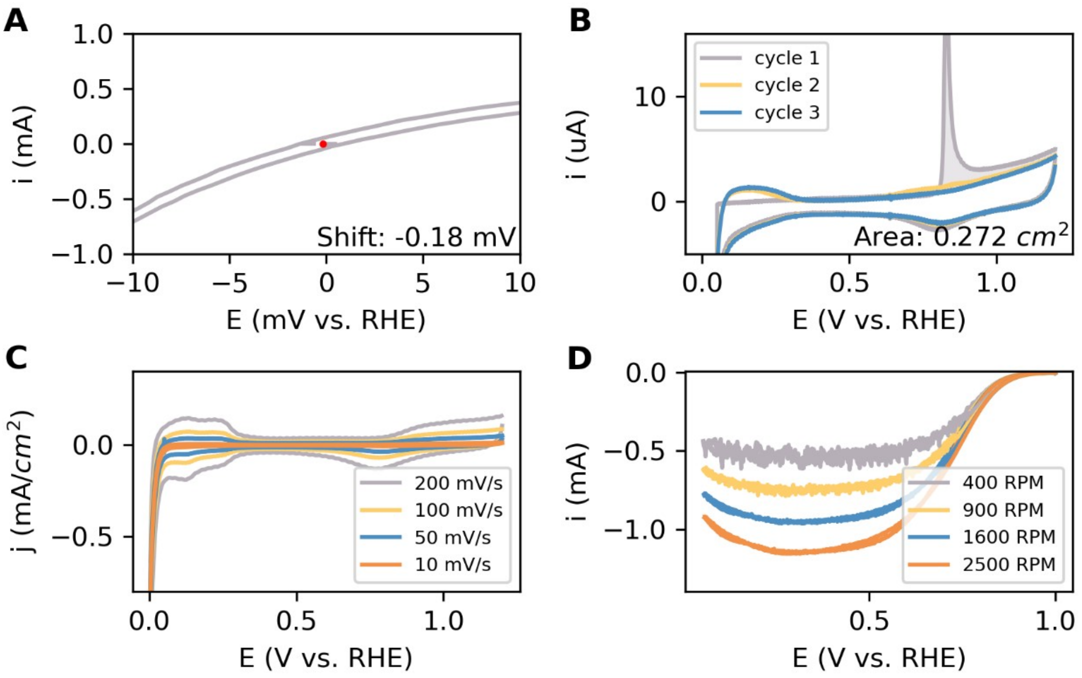

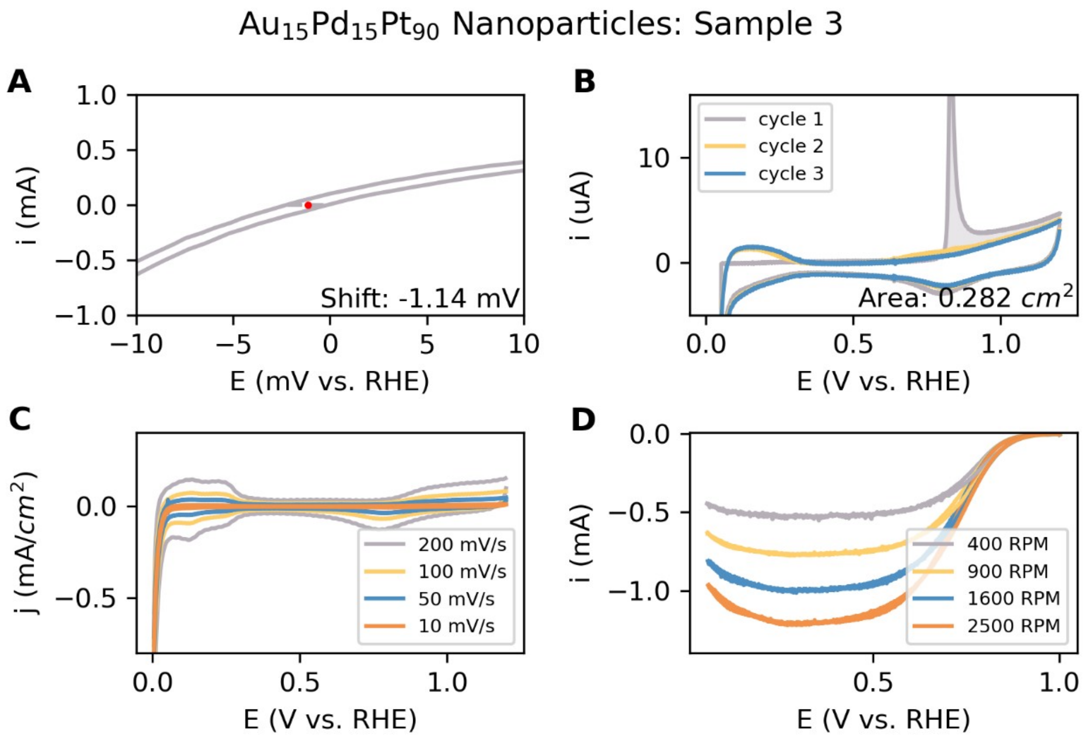

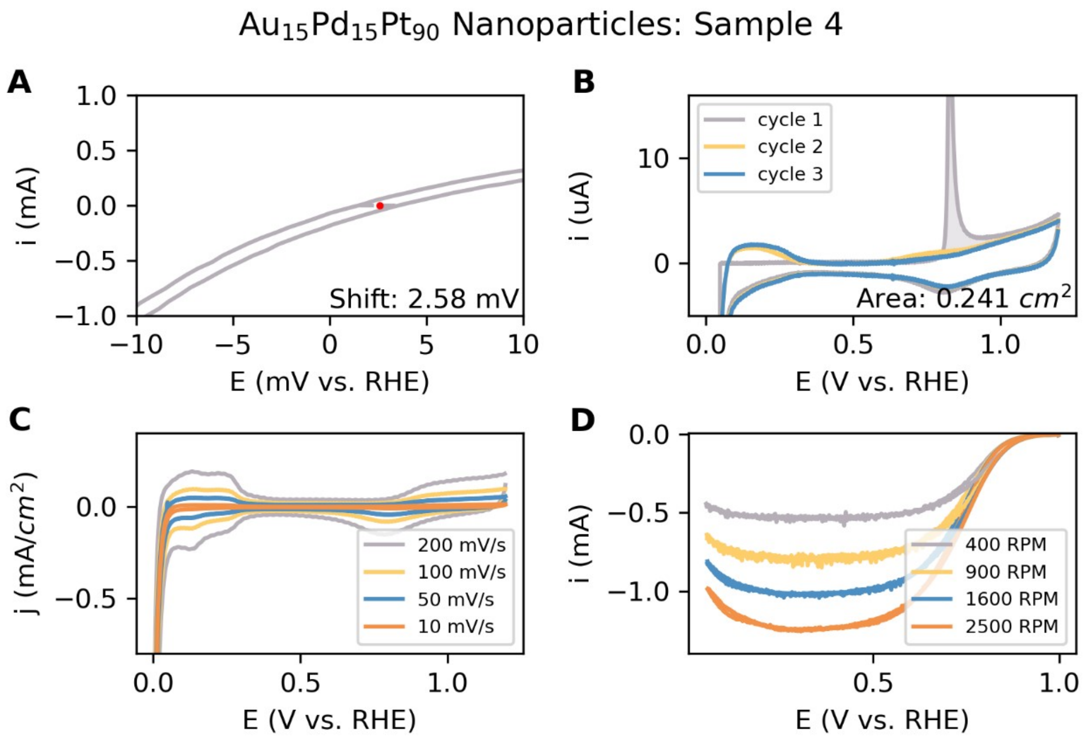

Fig S25. $Au_{15}Pd_{15}Pt_{70}$ electrochemistry measurements. A) The reference shift of the RHE electrode. **B)** The CO stripping experiment to determine the surface area. The integrated area is filled. **C)** CVs at different scan speeds. **D)** The ORR activity measurements at different rotation rates at 10 mV/s. The characterization CV in Ar was subtracted from these measurements. Also, these measurements are corrected for the solution resistance and reference shift.

Characterization

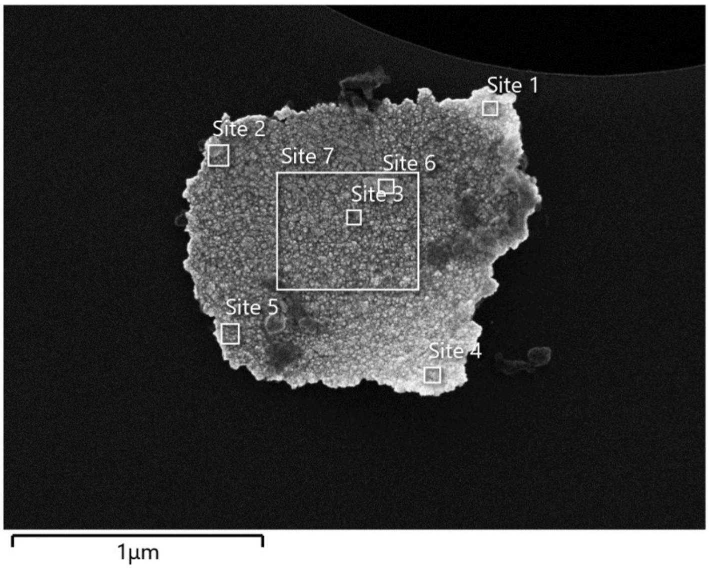

Site 1	$Au_{24.5}Pd_{12.4}Pt_{63.1}$
Site 2	$Au_{25.8}Pd_{12.3}Pt_{62.0}$
Site 3	$Au_{24.8}Pd_{13.1}Pt_{62.1}$
Site 4	$Au_{25.7}Pd_{12.6}Pt_{61.7}$
Site 5	$Au_{25.5}Pd_{12.2}Pt_{62.3}$
Site 6	$Au_{25.9}Pd_{11.3}Pt_{62.8}$
Site 7	$Au_{25.8}Pd_{11.4}Pt_{62.9}$

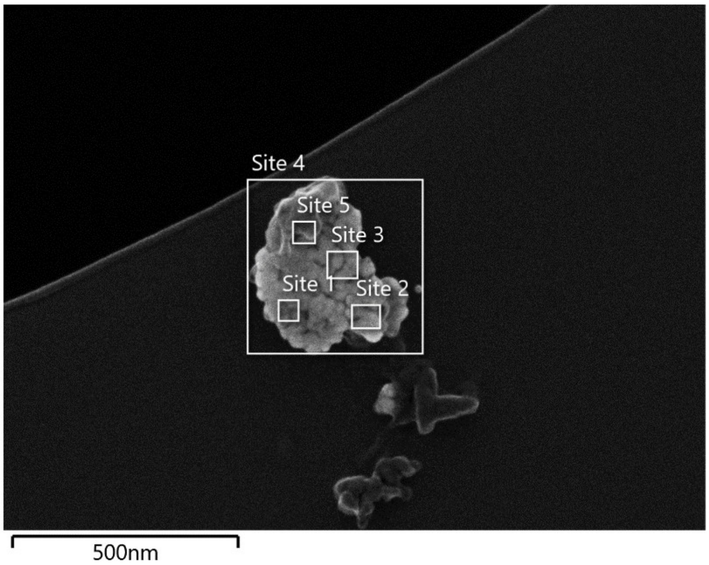

Site 1	$Au_{33.9}Pd_{2.7}Pt_{63.5}$
Site 2	$Au_{34.8}Pd_{1.9}Pt_{63.3}$
Site 3	$Au_{34.1}Pd_{3.0}Pt_{63.0}$
Site 4	$Au_{34.4}Pd_{2.8}Pt_{62.9}$
Site 5	$Au_{32.1}Pd_{3.3}Pt_{64.6}$

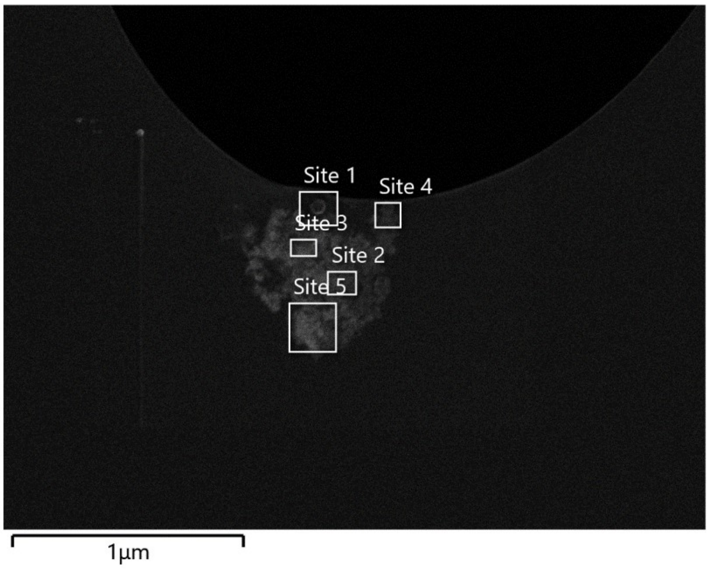

Site 1 $Au_{16.7}Pd_{9.6}Pt_{73.7}$

Site 2 $Au_{18.4}Pd_{9.1}Pt_{72.6}$

Site 3 $Au_{19.1}Pd_{9.8}Pt_{71.1}$

Site 4 $Au_{19.7}Pd_{11.8}Pt_{68.5}$

Site 5 $Au_{18.6}Pd_{10.0}Pt_{71.4}$

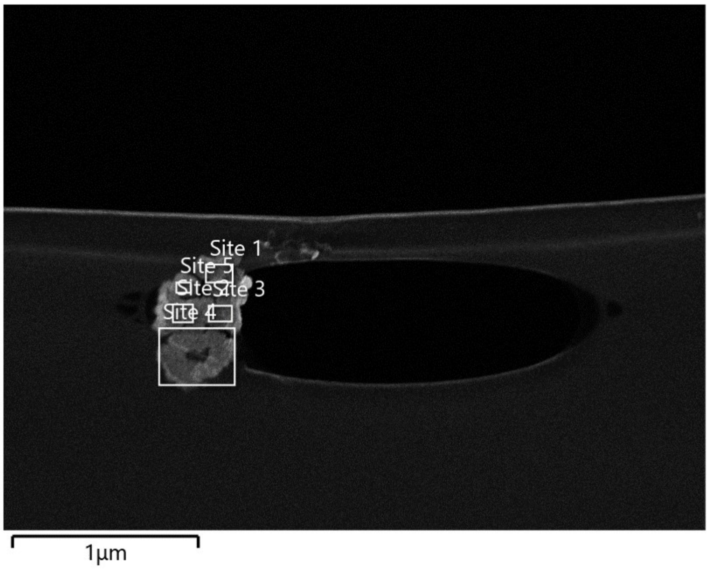

Site 1 $Au_{18.0}Pd_{17.8}Pt_{64.2}$

Site 2 $Au_{16.1}Pd_{24.7}Pt_{59.2}$

Site 3 $Au_{18.6}Pd_{23.1}Pt_{58.3}$

Site 4 $Au_{19.7}Pd_{19.6}Pt_{60.7}$

Site 5 $Au_{15.6}Pd_{28.3}Pt_{56.1}$

Fig S26. **SEM-EDX characterization of $Au_{15}Pd_{15}Pt_{70}$.** The average composition across all measured particles is $Au_{24.6}Pd_{11.0}Pt_{64.4}$.

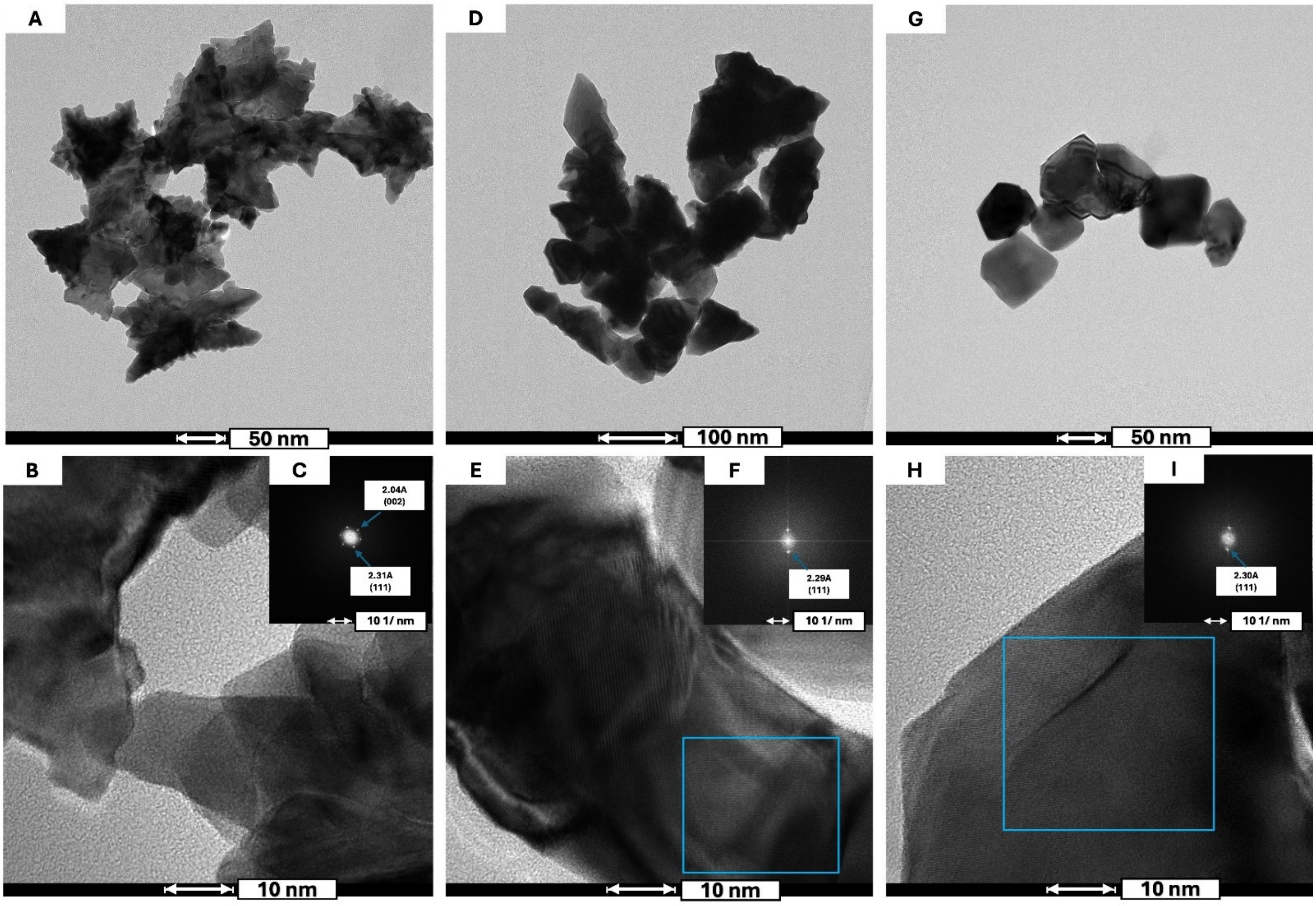

Fig S27. High resolution imaging of $Au_{15}Pd_{15}Pt_{70}$ nanoparticles. A, D, G) Bright Field Images at low magnification. **B, E, H)** the high-resolution transmission electron microscopy image of the respective areas in A, D and G. **C, F, I)** display the corresponding fast Fourier transform of B, E and H.

Nanoparticle Results

Electrochemistry

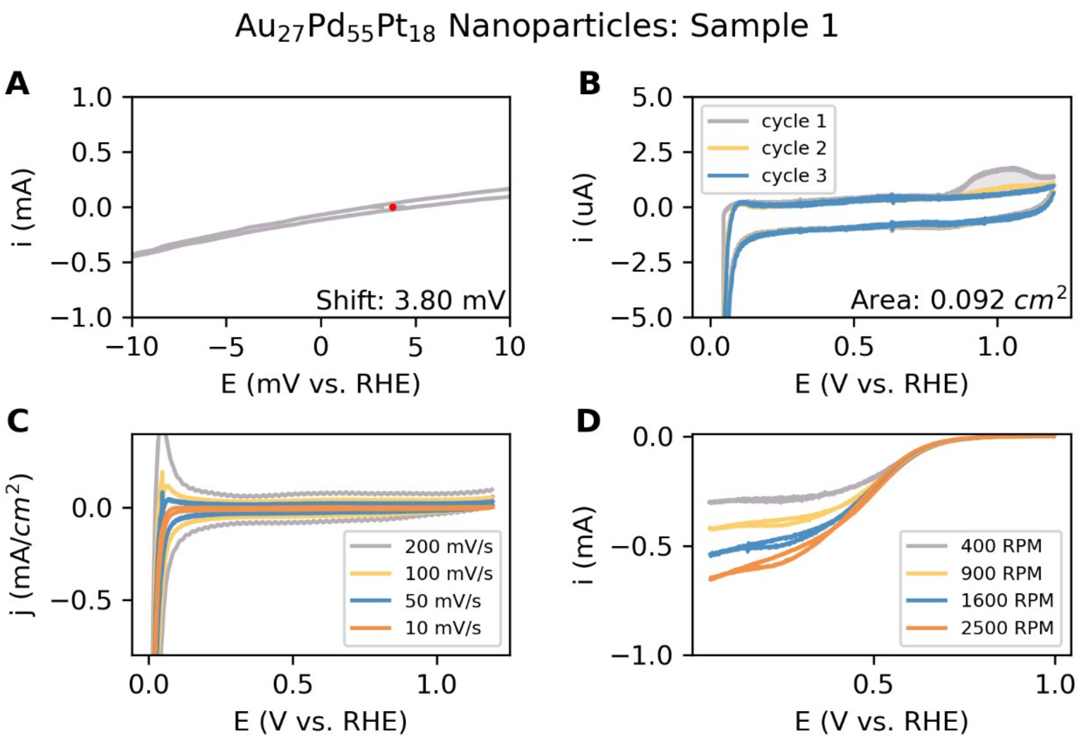

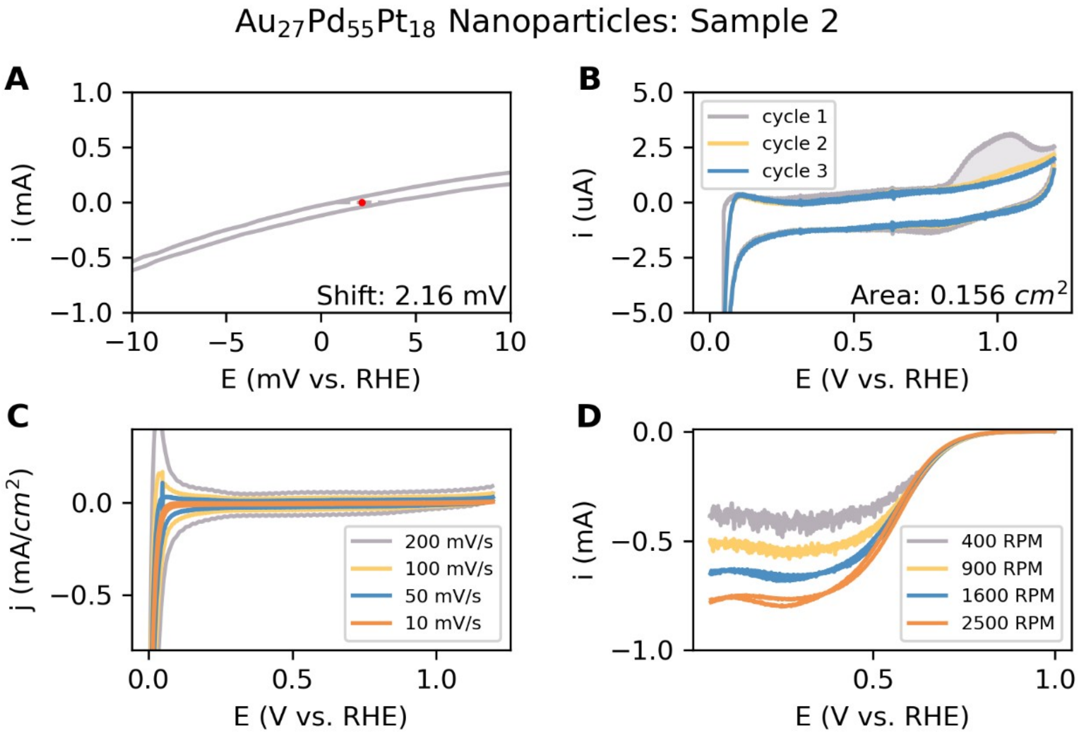

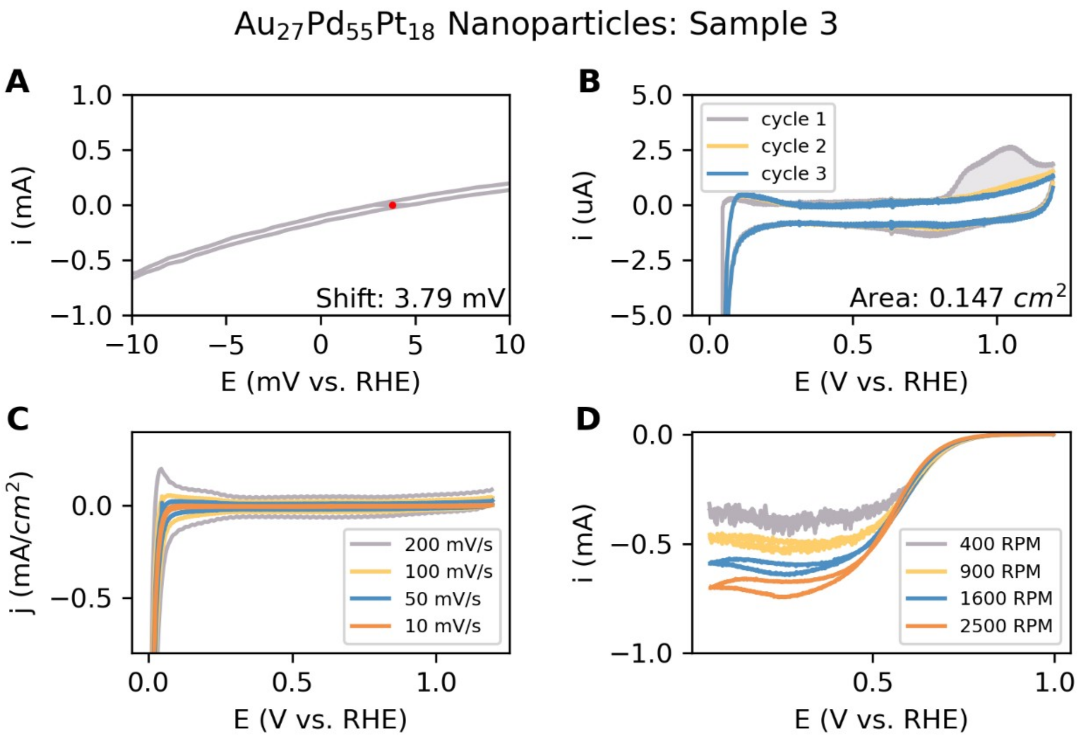

Fig S28. $Au_{27}Pd_{55}Pt_{18}$ electrochemistry measurements. A) The reference shift of the RHE electrode. **B)** The CO stripping experiment to determine the surface area. The integrated area is filled. **C)** CVs at different scan speeds. **D)** The ORR activity measurements at different rotation rates at 10 mV/s. The characterization CV in Ar was subtracted from these measurements. Also, these measurements are corrected for the solution resistance and reference shift.

Characterization

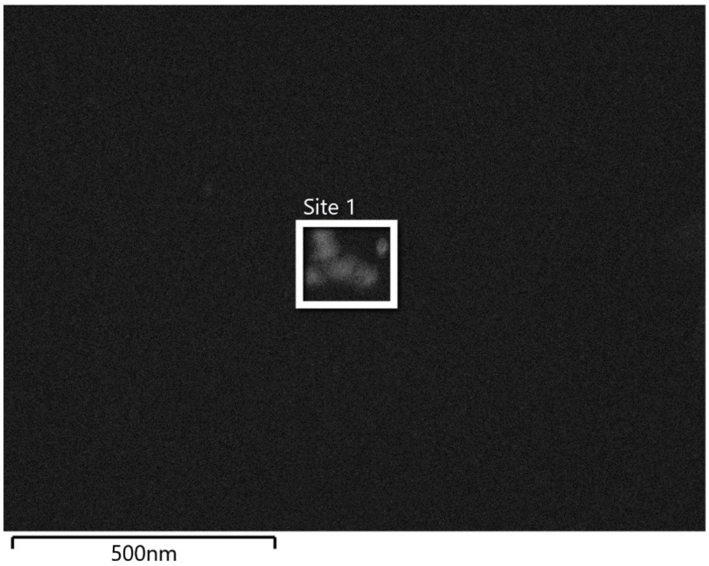

Site 1 $Au_{28.1}Pd_{62.6}Pt_{9.3}$

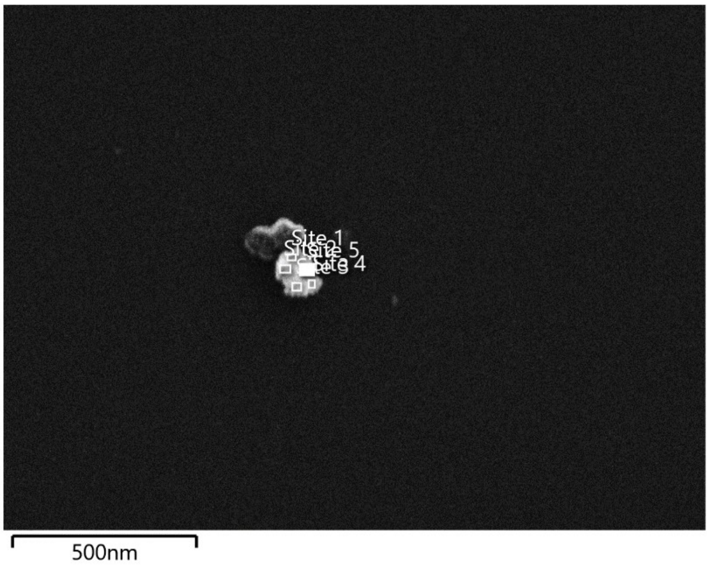

Site 1 $Au_{2.3}Pd_{69.6}Pt_{28.1}$

Site 2 $Au_{4.7}Pd_{65.5}Pt_{29.9}$

Site 3 $Au_{12.6}Pd_{72.1}Pt_{15.3}$

Site 4 $Au_{10.2}Pd_{72.0}Pt_{17.8}$

Site 5 $Au_{3.4}Pd_{72.3}Pt_{24.4}$

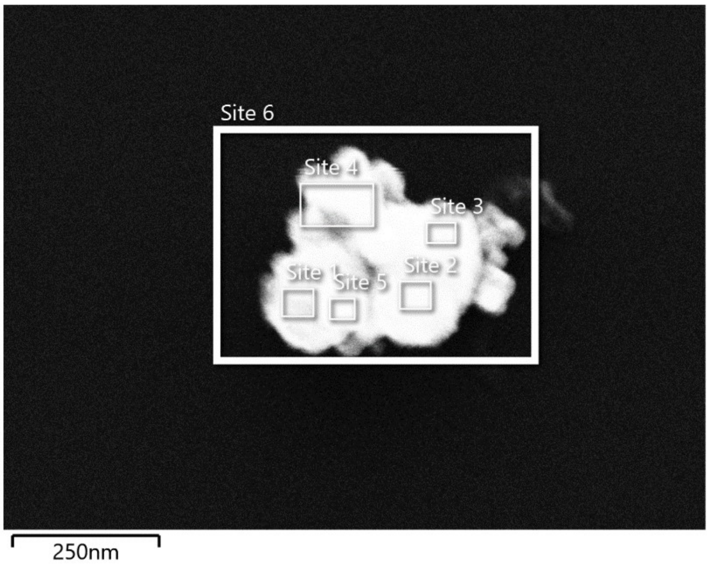

Site 1 $Au_{37.3}Pd_{55.6}Pt_{7.2}$

Site 2 $Au_{37.0}Pd_{54.0}Pt_{8.9}$

Site 3 $Au_{37.7}Pd_{53.2}Pt_{9.1}$

Site 4 $Au_{42.0}Pd_{52.4}Pt_{5.6}$

Site 5 $Au_{43.7}Pd_{50.4}Pt_{5.8}$

Site 6 $Au_{42.3}Pd_{50.3}Pt_{7.3}$

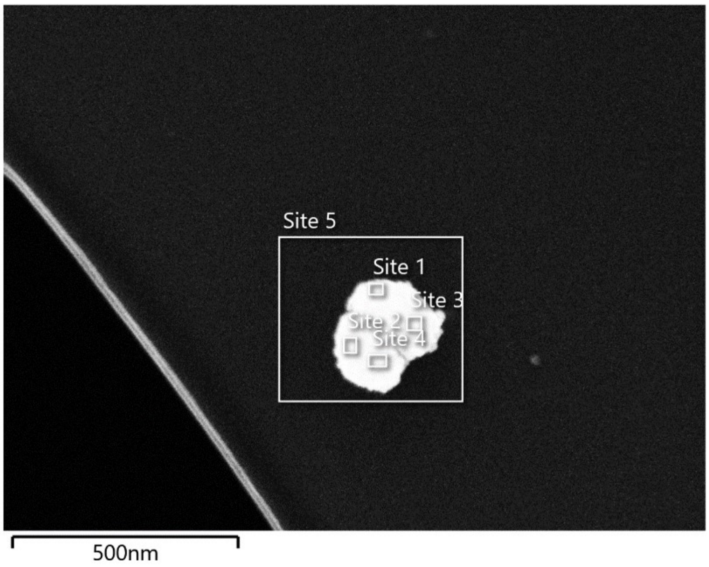

Site 1 $Au_{38.5}Pd_{40.5}Pt_{21.0}$

Site 2 $Au_{38.8}Pd_{40.8}Pt_{20.4}$

Site 3 $Au_{33.9}Pd_{43.7}Pt_{22.4}$

Site 4 $Au_{39.7}Pd_{37.9}Pt_{22.5}$

Site 5 $Au_{37.1}Pd_{40.5}Pt_{22.5}$

Fig S29. **SEM-EDX characterization of $Au_{27}Pd_{55}Pt_{18}$.** The average composition across all measured particles is $Au_{29.4}Pd_{56.4}Pt_{14.2}$.

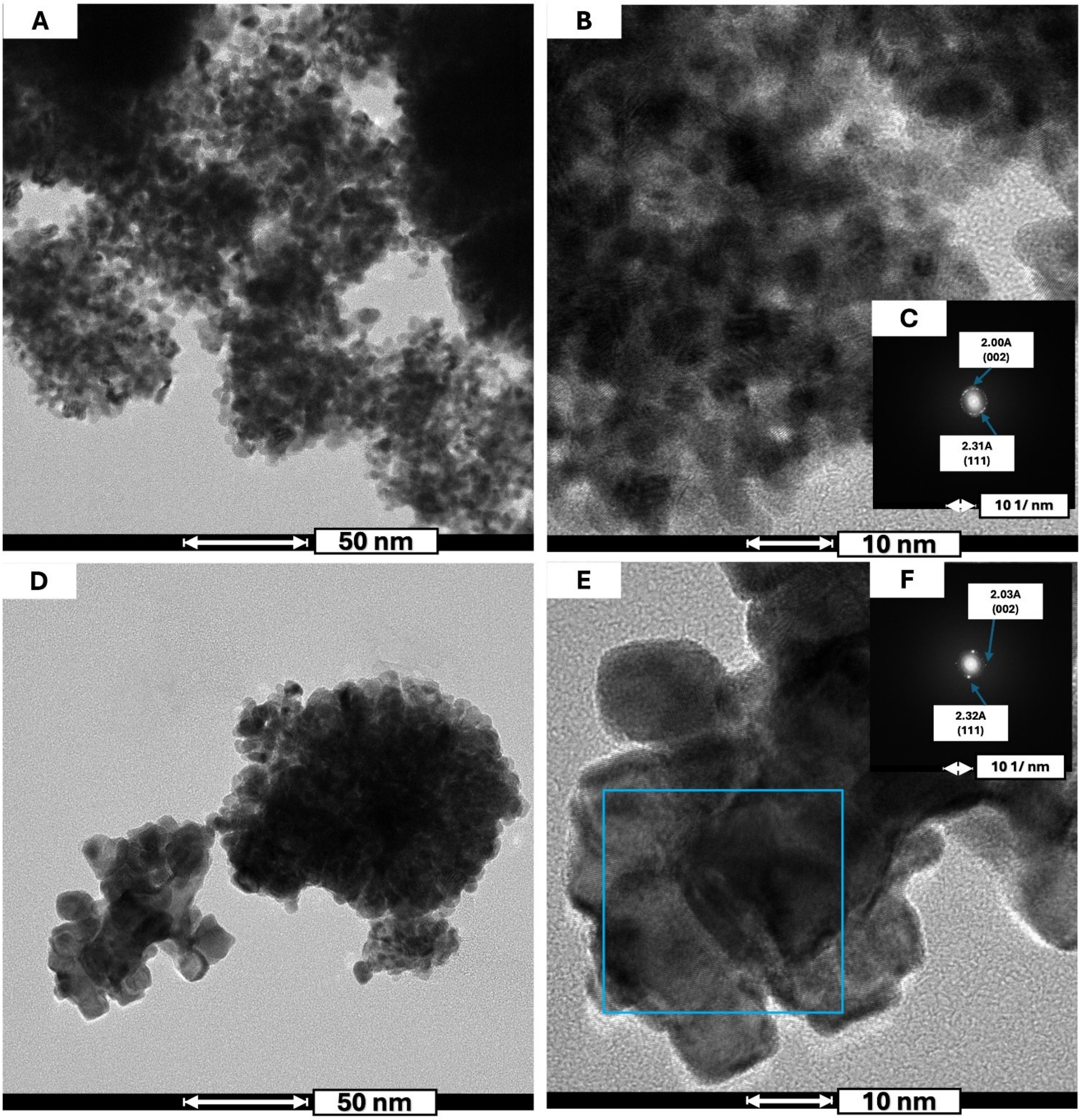

Fig S30. High resolution imaging of $Au_{27}Pd_{55}Pt_{18}$ nanoparticles. A, D) Bright Field Images at low magnification. **B, E)** shows the high-resolution transmission electron microscopy image of the respective areas in A and D. **C, F)** display the corresponding fast Fourier transform of B and E.

Pd Nanoparticles Results

Electrochemistry

Pd Nanoparticles: Sample 1

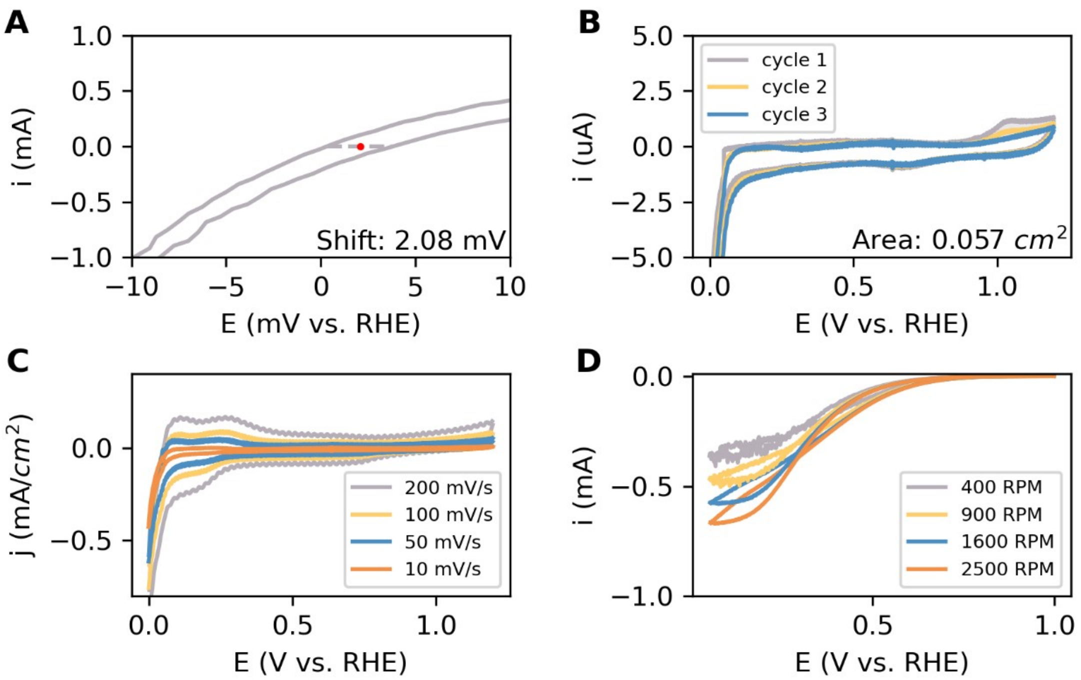

Pd Nanoparticles: Sample 2

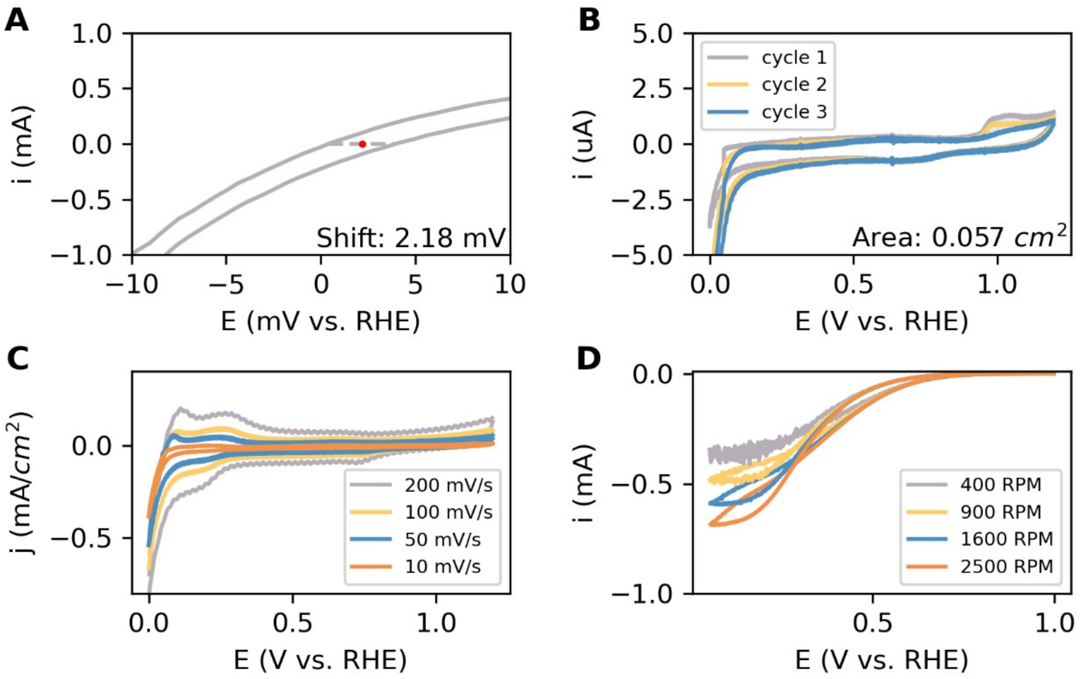

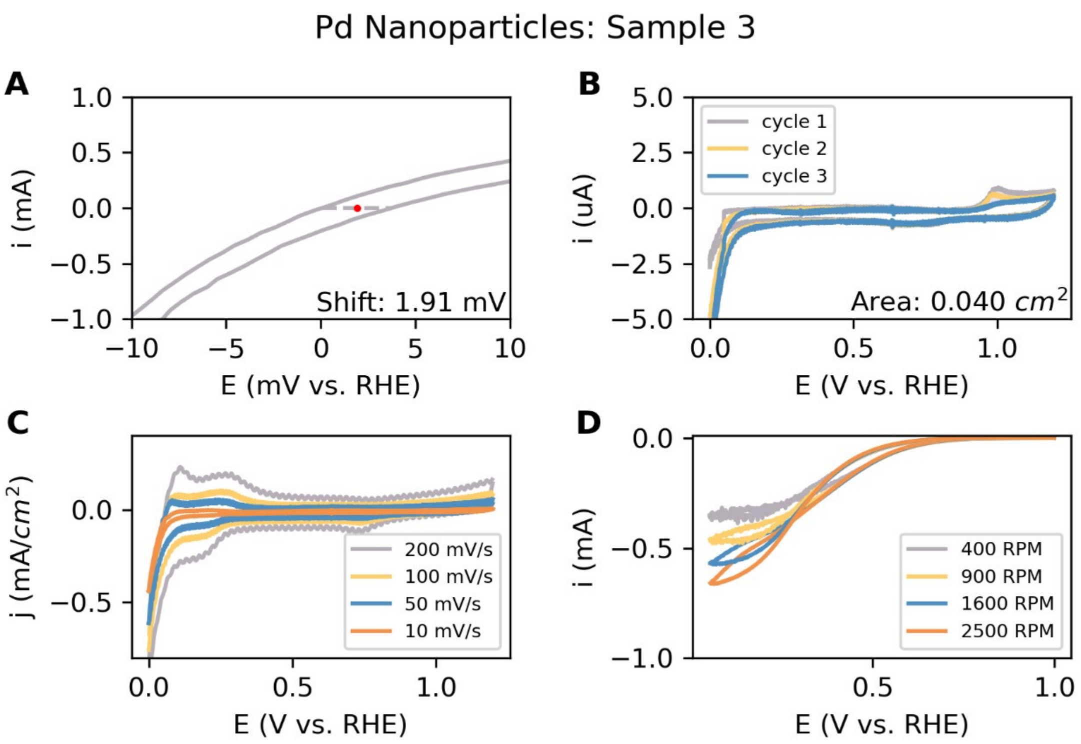

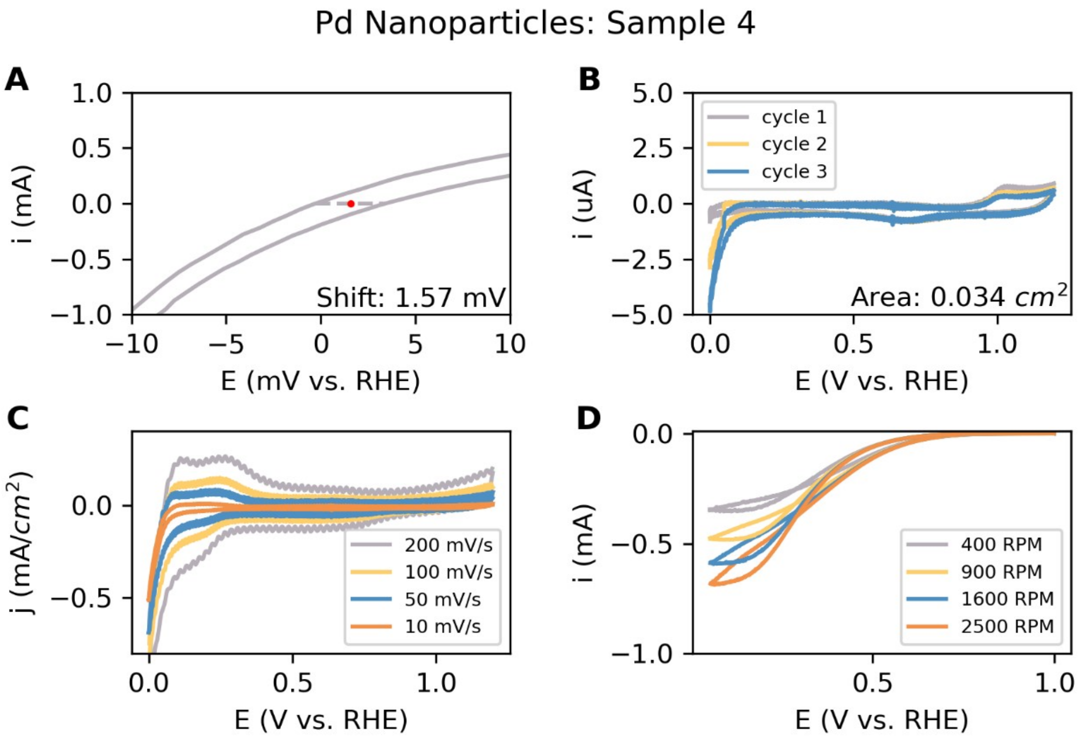

Fig S31. Pd electrochemistry measurements. A) The reference shift of the RHE electrode. **B)** The CO stripping experiment to determine the surface area. The integrated area is filled. **C)** CVs at different scan speeds. **D)** The ORR activity measurements at different rotation rates at 10 mV/s. The characterization CV in Ar was subtracted from these measurements. Also, these measurements are corrected for the solution resistance and reference shift.

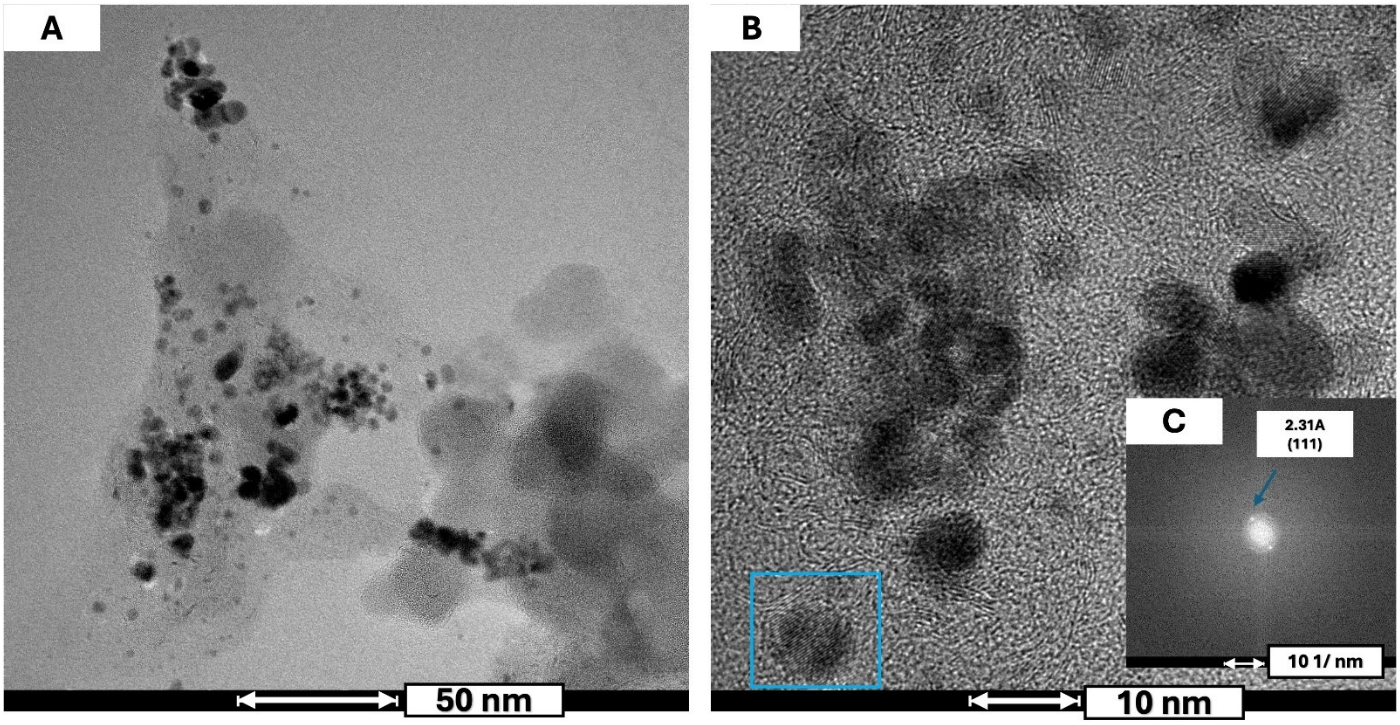

Fig S32. High resolution imaging Pd nanoparticles. A) Bright Field Image at low magnification. **B)** shows the high-resolution transmission electron microscopy image. **C)** the corresponding fast Fourier transform of B.

Pt(111) Results

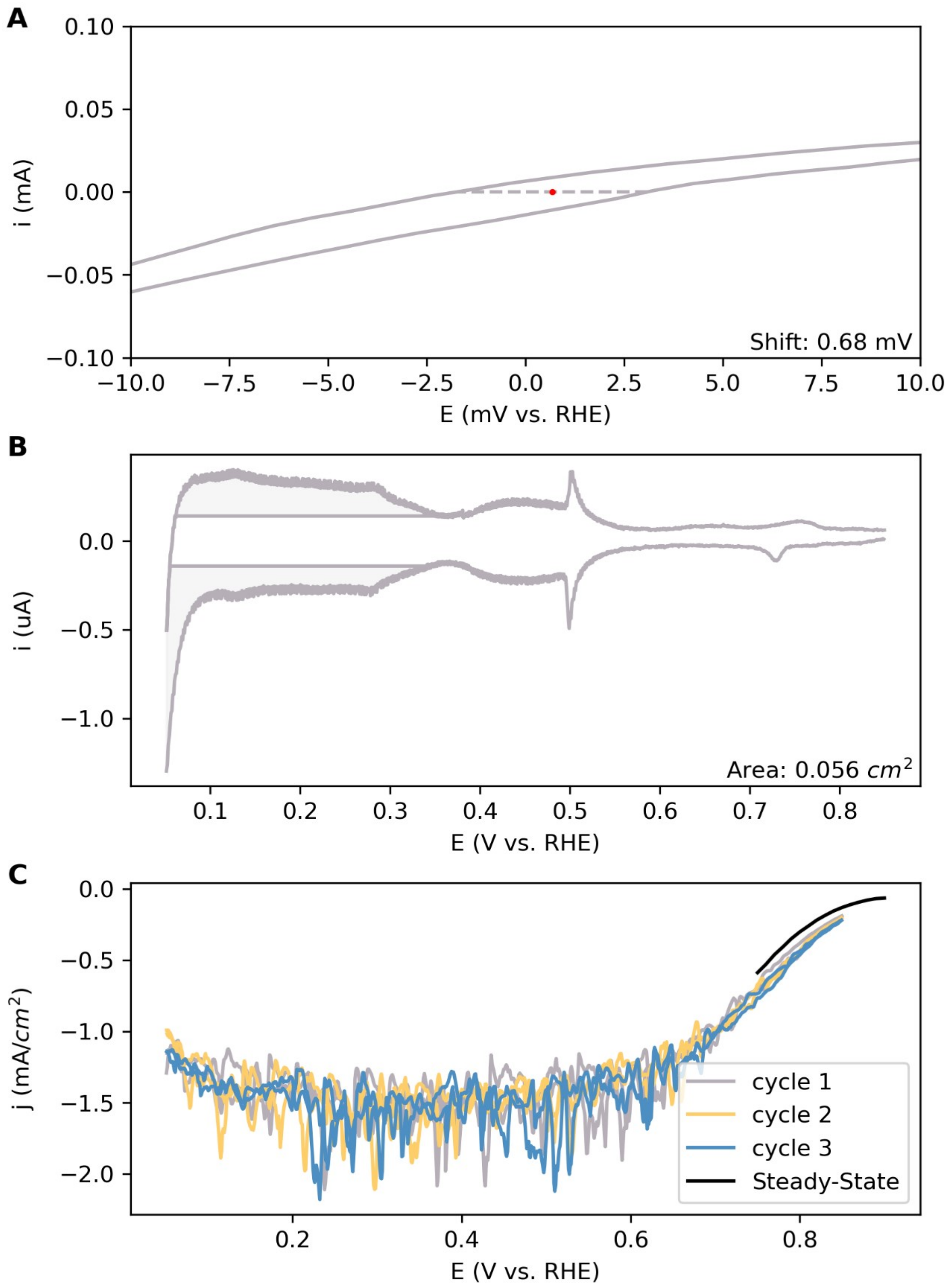

Fig S33. Pt(111) electrochemical measurements. A) The RHE reference shift during Pt(111) measurements. **B)** Pt(111) CV in 0.1 M H_2SO_4 at 10 mV/s. The surface area was determined by integrating the filled area in the hydrogen under potential region and dividing it by 320 μC cm^{-2} [12]. **C)** CVs of Pt(111) in O_2 measured at 10 mV/s. The data is corrected for the reference shift as well as for the Pt(111) CV. The Steady-State curve was obtained by averaging the current over the last 17 seconds of each potential step.

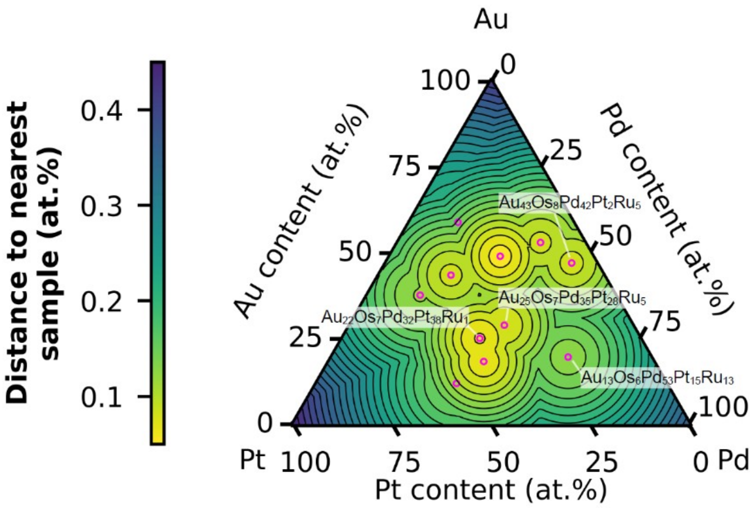

Fig S34. The closest measurement in the AuOsPdPtRu space to the coordinates in AuPtPd. The predicted optimum composition at $Au_{27}Pd_{55}Pt_{18}$ is closest to the measured sample $Au_{13}Os_{6}Pd_{53}Pt_{14}Ru_{13}$.

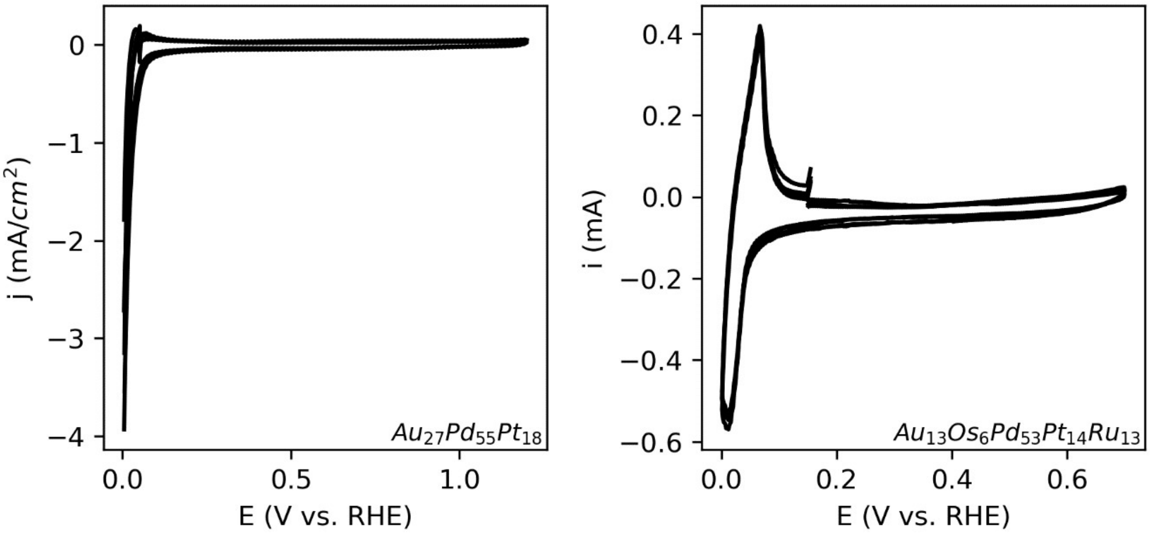

Fig S35. Comparing the characterization CV of $Au_{27}Pd_{55}Pt_{18}$ and $Au_{13}Os_{6}Pd_{53}Pt_{14}Ru_{13}$. Both CVs were recorded in 0.1 M H_2SO_4 at 100 mV/s. $Au_{13}Os_{6}Pd_{53}Pt_{14}Ru_{13}$ was synthesized using the solvothermal synthesis approach and used for the Gaussian Process fitting. It is located closes to the predicted optimum of $Au_{27}Pd_{55}Pt_{18}$ and probably affected the decision making. $Au_{27}Pd_{55}Pt_{18}$ was synthesized using the electrochemical deposition method. The difference in charge transferred to the hydrogen underpotential deposition region indicates that $Au_{13}Os_{6}Pd_{53}Pt_{14}Ru_{13}$ has a much larger fraction of Pd on the surface compared to $Au_{27}Pd_{55}Pt_{18}$. This would explain why $Au_{27}Pd_{55}Pt_{18}$ is performing bad as a catalyst.

VII. Model without capacitance normalization.

The effect of the capacitance normalization was further investigated by fitting the Gaussian process on non-normalized data. The parity plots of which are shown in Fig. S35. The 8-element model performs similarly to the normalized model with an R^2 of 0.541. Furthermore, the optima of the non-normalized model follow a very similar ranking as of the normalized model as demonstrated in Fig. S36. Similarly, Pt-Pd-Au is the most active combination followed by Pt-Au and Pt-Pd combinations. This suggests that the mathematical landscapes are relatively similar. This similarity however changes for the 5-element models. In fact, the absence of normalization leads to a significant worsening of the **Ir-Os-Pd-Pt-Ru** and Ir-Os-Pt-Rh-Ru models. This worsening affects both the prediction from the 8-element model as well as the LOOCV score of the 5-element models themselves. This suggests that these datasets have an increased noise level that the normalization improves, justifying the capacitance normalization. Additionally, it can be argued that the 8-element model is more noise resilient due to being composed of more data points. Hence the normalization has a smaller effect on the 8-element model compared to the 5-element models.

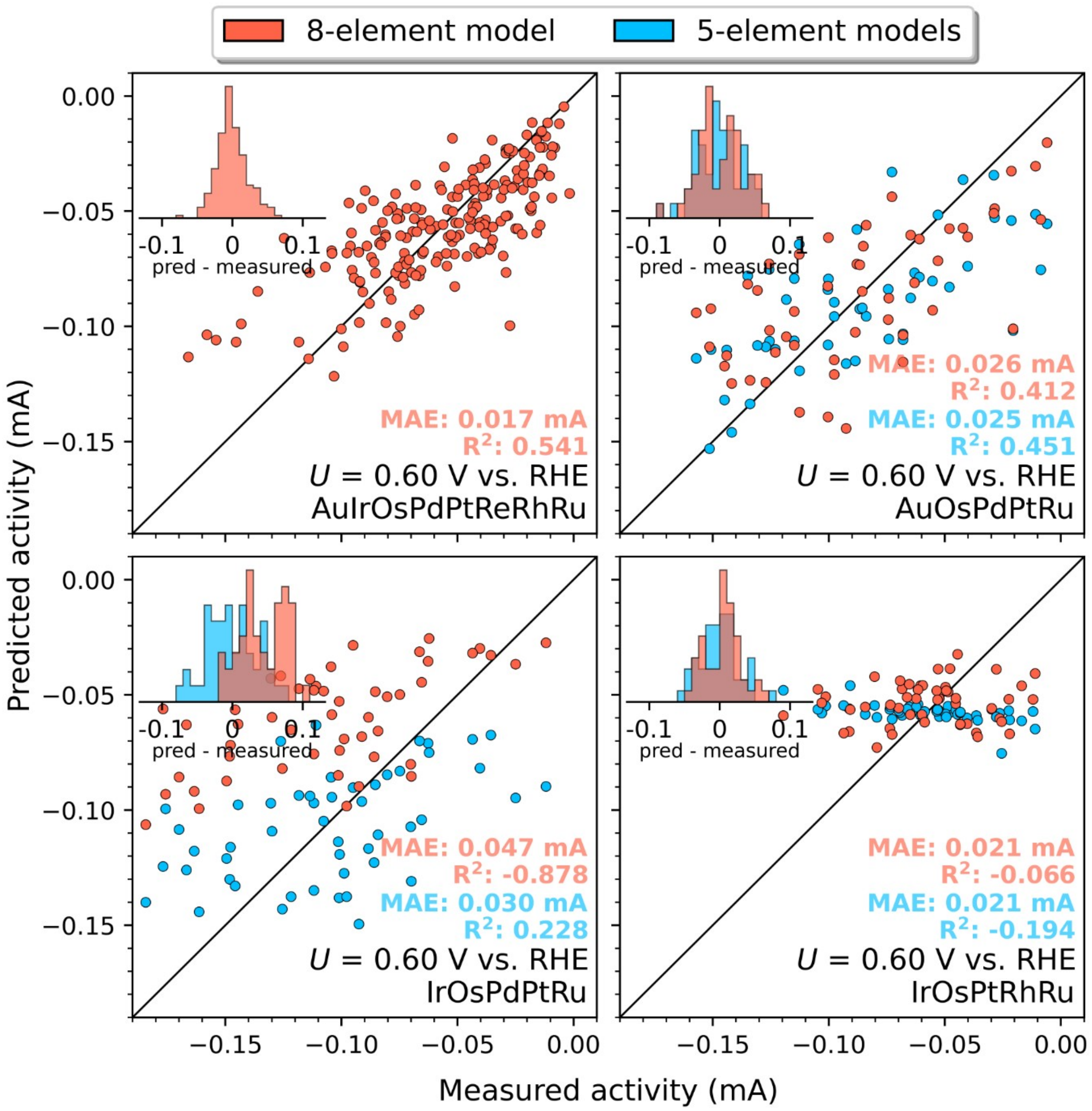

Fig S36. Parity plots of the models without performing a capacitance normalization. Predicted vs. measured current per capacitance for each of the four investigated alloy systems. The prediction was evaluated using the leave-one-out cross-validation. In addition, the subplots with the 5-element models show how the 8-element model is predicting the same data points.

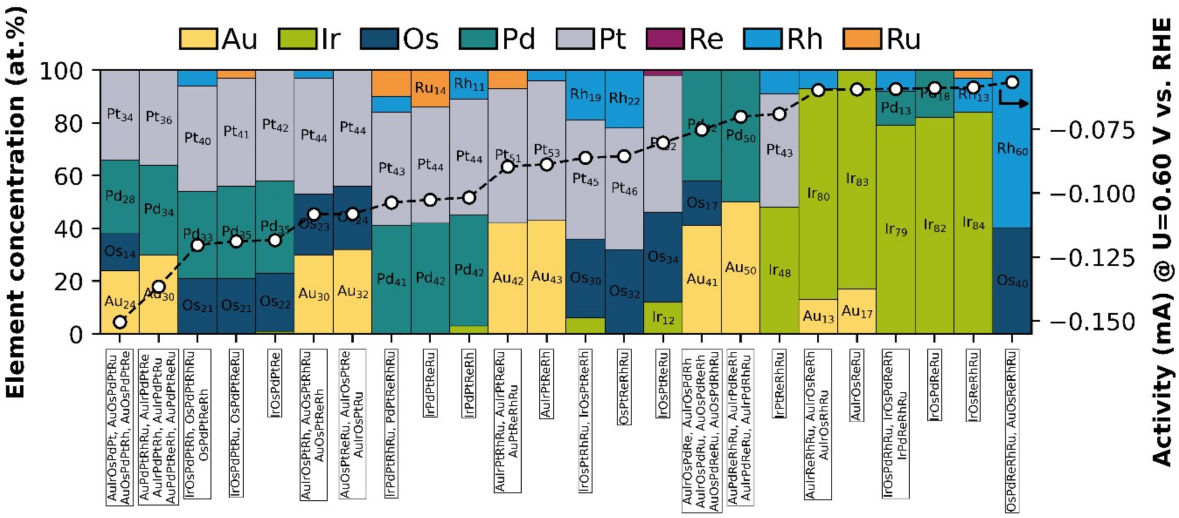

Fig S37. Optima in the composition spaces predicted by the non-normalized 8-element model.

References

1. Hastie, T., Tibshirani, R. & Friedman, J. Local Methods in High Dimensions. in *The Elements of Statistical Learning* 22–27 (Springer Science+Business Media, New York, 2017).
2. Pedersen, J. K. *et al.* Bayesian Optimization of High-Entropy Alloy Compositions for Electrocatalytic Oxygen Reduction**. *Angewandte Chemie International Edition* **60**, 24144–24152 (2021).
3. Coelho, A. A. *TOPAS* and *TOPAS-Academic* : an optimization program integrating computer algebra and crystallographic objects written in C++. *J Appl Crystallogr* **51**, 210–218 (2018).
4. Yeh, J.-W. Recent progress in high-entropy alloys. *Annales de Chimie Science des Matériaux* **31**, 633–648 (2006).
5. Wu, D. *et al.* Noble-Metal High-Entropy-Alloy Nanoparticles: Atomic-Level Insight into the Electronic Structure. *J Am Chem Soc* **144**, 3365–3369 (2022).
6. Broge, N. L. N., Bertelsen, A. D., Søndergaard-Pedersen, F. & Iversen, B. B. Facile Solvothermal Synthesis of Pt–Ir–Pd–Rh–Ru–Cu–Ni–Co High-Entropy Alloy Nanoparticles. *Chemistry of Materials* **35**, 144–153 (2023).
7. Rasmussen, C. Edward. & Williams, C. K. I. Chapter 5: Model Selection and Adaptation of Hyperparameters. in *Gaussian processes for machine learning* 105–128 (the MIT Press, 2006).
8. Bondesgaard, M., Broge, N. L. N., Mamakhel, A., Bremholm, M. & Iversen, B. B. General Solvothermal Synthesis Method for Complete Solubility Range Bimetallic and High-Entropy Alloy Nanocatalysts. *Adv Funct Mater* **29**, (2019).
9. Sobol', I. M. On the distribution of points in a cube and the approximate evaluation of integrals. *Zhurnal Vychislitel'noi Matematiki i Matematicheskoi Fiziki* **7**, 784–802 (1967).
10. Sobol', I. M. On the distribution of points in a cube and the approximate evaluation of integrals. *USSR Computational Mathematics and Mathematical Physics* **7**, 86–112 (1967).
11. Shinozaki, K., Zack, J. W., Richards, R. M., Pivovar, B. S. & Kocha, S. S. Oxygen Reduction Reaction Measurements on Platinum Electrocatalysts Utilizing Rotating Disk Electrode Technique: I. Impact of Impurities, Measurement Protocols and Applied Corrections. *J Electrochem Soc* **162**, F1144–F1158 (2015).
12. Marković, N. M., Lucas, C. A., Climent, V., Stamenković, V. & Ross, P. N. Surface electrochemistry on an epitaxial palladium film on Pt(111): surface microstructure and hydrogen electrode kinetics. *Surf Sci* **465**, 103–114 (2000).
